# The effects of different types of leisure-time physical activity on positive mental health among adolescents: a mixed-methods systematic review and meta-analysis

**DOI:** 10.1186/s12966-025-01834-4

**Published:** 2025-10-07

**Authors:** Lars Lenze, Valentin Benzing, Julia Schmid, Beatrice Minder, Rosa-Emilia Henn, Annika Frahsa

**Affiliations:** 1 Institute of Social and Preventive Medicine, University of Bern, Bern, Switzerland; 2 Institute of Sport Science, University of Bern, Bern, Switzerland; 3 Public Health & Primary Care Library, University Library of Bern, University of Bern, Bern, Switzerland

**Keywords:** Mental health, Well-being, Physical activity, Leisure-time physical activity, Sports participation, Adolescence, Youth, Systematic review, Mixed-methods, Meta-analysis

## Abstract

**Background:**

Despite the well-researched general effect of physical activity on mental health, less is known about specific effects, such as qualitative and contextual aspects of physical activity. Thus, this review aimed to systematically synthesise evidence on the effects and experiences of different types of leisure-time physical activity (LTPA; e.g., running, fitness, yoga) on distinct positive mental health (PMH) outcomes among adolescents.

**Methods:**

We searched in seven databases (MEDLINE, Embase, PsycINFO, Cochrane, SPORTDiscus, CINAHL, and Web of Science) without language restrictions for records from January 2009 to 16 June 2025. Inclusion criteria were defined according to the PICOS framework: population (adolescents from non-clinical studies); intervention (specific LTPA type); comparisons (with and without comparator); outcomes (measures or experiences of PMH); study designs (longitudinal quantitative and qualitative studies). We appraised included studies using the mixed-methods appraisal tool.

**Results:**

44 articles from initial 8,149 records were included. Following a convergent segregated approach, the meta-analyses for synthesising the quantitative studies showed different effects depending on the LTPA type. Additionally, the effects depend on the PMH outcome in focus. We identified four facilitators to promote PMH outcomes from the synthesis of qualitative studies: social facilitators for all types of LTPA and various configurations of other facilitators (mastery-related, setting-related, affective-related) for specific LTPA types. The mixed-methods synthesis shows the interplay between LTPA and PMH outcomes depending on the LTPA type.

**Conclusions:**

The effects of LTPA on PMH in adolescents depend on the LTPA type and PMH outcome in focus. Perceived facilitators highlight possible explanations for the effects found. By investigating specific LTPA types and different PMH outcomes, ecologically valid implications for specific contexts to foster adolescent mental health may be derived. The limited number of studies per LTPA type, PMH aspect, and study design emphasises the need for more research to derive more specific and robust implications for tailored mental health promotion.

**Supplementary Information:**

The online version contains supplementary material available at 10.1186/s12966-025-01834-4.

## Background

Mental health research focus has shifted from the absence of mental disorders to Positive Mental Health (PMH) [1, 2]. The World Health Organisation (WHO) defines mental health as “a state of well-being in which the individual realizes his or her own abilities, can cope with the normal stresses of life, can work productively and fruitfully, and is able to make a contribution to his or her community” [3, 4]. For PMH, various terms (e.g., subjective, emotional, or psychological well-being) from different research streams (e.g., hedonic and eudaimonic well-being) are discussed [5, 6]. Park et al. [6] distinguish between two key features of well-being: (1) *experiential* features, encompassing affective and emotional aspects of experiences; and (2) *reflective* features including cognitive evaluations about life satisfaction, sense of meaning, and perceived coping abilities. In addition to these features, PMH spans multiple domains, including emotional, psychological, and social well-being [5]; in physical activity research, physical well-being is also recognised as a relevant well-being domain [7, 8].

Fostering PMH also acts as a protective factor for mental illnesses [9], and is highly important amid the current mental health burden [10], especially in young people [11], with at least one in five adolescents facing mental health problems [12, 13].

One promising way of preventing mental disorders and fostering PMH is Physical Activity (PA) [14]. Caspersen et al. [15] defined PA as “any bodily movement produced by skeletal muscles that results in energy expenditure” (p. 126). It is widely acknowledged that PA has beneficial effects on adolescents’ mental health [16–20]. Traditionally, research – based on a predominantly biomedical perspective – has focused on quantitative characteristics of PA, such as frequency, duration, and intensity [20]. In recent years, however, this perspective has been expanded to include contextual factors (e.g., the social and environmental setting) and individuals’ subjective experience (e.g., enjoyment, autonomy, perceived competence) related to PA in general [21] and studies focusing on PA and mental health outcomes specifically [20]. Similarly, current literature on the mechanisms linking PA and mental health highlights not only (neuro-)biological processes but increasingly also considers psychological, social, behavioural, and environmental factors [22–24]. Overall, there is a shift in PA research toward a more comprehensive and integrative understanding of its effects on mental health.

To structure contextual factors of PA on mental health outcomes, Vella and colleagues [20] differentiate between (1) type, (2) domain, (3) physical environment, (4) social environment, and (5) delivery of PA. With regard to domain, leisure has been identified as the life context in which PA yields the most promising effects on mental health [25, 26]. Biddle and Mutrie [27] advocate focusing on leisure-based activity to promote health, highlighting in particular the relevance of psychological and social mechanisms. Accordingly, this review concentrates on Leisure-Time Physical Activity (LTPA).

The type (e.g., yoga, football) is another characteristic of PA, categorised as a contextual factor too [20] or as a qualitative characteristic [28]. Considering the LTPA type and its effects on mental health outcomes, previous reviews investigated (1) individual and team sports [19, 29–32] or team sports only [33]; (2) aerobic exercise compared to sports [32] or to anaerobic exercise [29]; (3) aesthetic activities [19, 31]; (4) so-called ‘informal and lifestyle sports’ (e.g., climbing, parkour, surfing [34]); (5) resistance training [35]; (6) dancing [36–38]. A recent umbrella review showed benefits from different types of PA on various aspects of well-being and ill-being in general and clinical populations [20]. This umbrella review covered all age groups, but it was based on a low number of adolescent studies, mainly carried out in a clinical setting. In summary, there are reviews about selected differentiating aspects of the LTPA type, such as team and individual sports, and for some specific LTPA types (i.e. resistance training and dancing) on (positive) mental health outcomes. However, a comprehensive and nuanced overview including various LTPA types is missing, which is required to better understand this phenomenon [8, 16]. By focusing on LTPA and its specific types, an interplay of various contextual factors is evident, which is more than its single aspects: For instance, football typically involves outdoor settings (physical environment) and social interaction with team members (social environment). Additionally, quantitative factors are related when practising a given LTPA type (e.g., playing football is related to a certain intensity). This interplay of contextual and also quantitative factors shapes the experience when practising a certain LTPA type, influences psychosocial processes, and potentially affects mental health and PMH outcomes. Consequently, the LTPA type encompasses multiple PA characteristics and mirrors a rather holistic understanding of PA as proposed by Piggin [21] than focusing on separate aspects (see also Hsu et al. [39]).

In conclusion, the interplay of PA characteristics in different LTPA types may trigger distinct psychosocial processes, leading to varying PMH outcomes. It remains unclear to what extent specific LTPA types are associated with subjective experiences and PMH outcomes in adolescents, or whether these associations are consistent across LTPA types. Given the relevance of this knowledge to inform targeted interventions and programmes promoting adolescent PMH, several key research gaps must be addressed: (1) To date, no systematic review has comprehensively examined and differentiated the effects and experiences of various LTPA types on adolescent PMH. (2) Existing reviews often focus exclusively on ill-being or take a more general approach by not considering different facets of PMH. (3) Most reviews draw exclusively on quantitative studies, overlooking qualitative research that could deepen understanding of subjective experiences. (4) Existing reviews often include mixed populations from both clinical and non-clinical studies, making it difficult to generalise findings to the general population.

Consequently, we aim to synthesise existing evidence on how specific LTPA types are linked to distinct aspects of PMH in adolescents. For providing a comprehensive and nuanced overview, we include both longitudinal quantitative and qualitative studies, also known as a mixed-methods review [40, 41]. By only considering longitudinal studies, we minimise the potential reverse effect of our research aim (i.e. mental health promotes PA). In doing so, evidence on effectiveness from quantitative intervention and observation studies (what-knowledge according to Sandelowski et al. [42]) is combined with insights about subjective experiences from qualitative studies (why-knowledge according to Sandelowski et al. [42]).

## Methods

### Design, protocol and registration

We conducted a mixed-methods systematic review [40, 41], following the Preferred Reporting Items for Systematic Reviews and Meta-Analyses (PRISMA) 2020 statement [43] (see Supplementary Table S1). Additionally, we also applied the ENTREQ guideline (ENhancing Transparency in REporting the synthesis of Qualitative research [44]) for the qualitative part (see Supplementary Table S2). The study protocol was registered a priori in PROSPERO (CRD42024550490) on June 22, 2024. Deviations from the study protocol are reported in Supplementary Section S3.

### Eligibility criteria

Following the PICOS framework, we included studies if they met the following criteria:

Population: Adolescents aged 10–19 years (following the WHO definition of adolescence) from non-clinical studies. Excluded were adolescents with a diagnosed illness/disorder.

Interventions/exposures: LTPA such as football, yoga, running or resistance training. Excluded were cool-down exercises, muscle stretching exercises, post-exercise recovery, pre-exercise and warm-up exercises.

Comparisons: In quantitative studies, mean differences between pre-post-tests and, in studies with control groups, mean differences between intervention and control group; no comparator in qualitative studies.

Outcomes: Measures or experiences of positive mental health and well-being, including psychological, emotional, social, and physical well-being, as well as constructs such as self-concept and its subcategories (e.g., physical self-concept); excluding studies that focused on mental disorders or illnesses (e.g., depression, anxiety disorders).

Study design: Original, peer-reviewed studies with quantitative, qualitative, or mixed-methods designs, including qualitative methods (e.g., interviews, focus groups), observational studies, and quantitative studies (e.g., cohort studies, longitudinal studies, (non-)randomised controlled trials, and pre-post intervention designs), while excluding cross-sectional studies to avoid potential reverse effect confounding.

We included studies published between 2009 and 2025 to minimise the impact of social and time-historical changes and ensure ongoing relevance of findings [45]. A detailed decision tree outlining the inclusion and exclusion criteria is available in the Supplementary Table S4.

### Information sources and search strategy

We systematically searched seven databases - MEDLINE, Embase, PsycINFO, Cochrane, SPORTDiscus, CINAHL, and Web of Science - without language restrictions, for records from January 2009 to 16 June 2025. Detailed search strategies for each database are provided in the Supplementary Table S5.

We used the automated deduplication software Deduklick to remove duplicate records [46]. We performed a forward citation search using Citationchaser [47] and hand-searched the included studies’ reference lists.

### Screening process

For title-abstract-screening, we analysed records using Automated Systematic Review (ASReview) - an open-source machine learning tool that ranks titles and abstracts, based on their relevance, using prior information provided by the reviewers [48]. Following the SAFE procedure [49], two authors (LL, RH) independently assessed a part of all records in a four-step procedure (for details see Supplementary Section S6). After reaching consensus on eligible records from the title-abstract-screening, two assessors (LL and AF, JS or VB) independently performed the full-text-screening in Covidence. We discussed discrepancies to reach consensus. For the updated search, we conducted title-abstract-screening and full-text-screening in Covidence only.

### Quality assessment

In accordance with the PRISMA protocol and amid the heterogeneity of the study designs, we used the Mixed-Methods Appraisal Tool (MMAT) [50] to assess the quality of the studies. After two general screening questions, five study design-specific criteria were rated and provided an overview of the studies’ quality. As suggested, the authors discussed the criteria and, to reduce a high variability in the interpretation of the criteria, created a consolidated version of appropriate criteria for the scope of the review (see Supplementary Table S7). Two authors (LL, RH) independently assessed the studies and achieved an agreement of κ = 0.80. Authors discussed and resolved discrepancies.

### Data extraction

Two investigators (LL, RH) independently extracted data from 20% of the included articles. After achieving an agreement rate of at least 80%, a single investigator (LL) completed data extraction [51] on authors, publication year, country, title, research aim, study design, sample information, operationalisation of LTPA, information about the intervention/exposure, operationalisation of LTPA, analysis approach, and main findings. From the qualitative studies, we extracted main findings related to the themes reported by the studies’ authors (second-order constructs) including relevant quotes from the participants (first-order constructs) [52].

### Data synthesis

We applied a ‘convergent segregated approach’ [41] and conducted a separate quantitative and qualitative synthesis first, followed by a mixed-methods meta-synthesis [40, 41] to “contextualize quantitative data and generate reasons behind the success and/or failure of a programme” (Pearson et al. [40] p. 123).

We categorised various LTPA types based on Sudeck et al. [53]. There are multiple options to structure LTPA types rather than one single consensual categorisation in this field of research. We opted for this categorisation as it allows (1) reflecting a broad understanding of LTPA, (2) grouping LTPA types based on qualitative and contextual characteristics of the activity, and (3) considering a categorisation from the intersection of exercise psychology and health promotion. Consequently, the following categories for LTPA types are distinguished: (1) walking and endurance activities (e.g., running, swimming); (2) fitness (e.g., strength training); (3) compositional-creative activities (e.g., dancing, artistic gymnastics); (4) relaxation-oriented activities (e.g., yoga, pilates); (5) outdoor and mountain activities (e.g., surfing, hiking); (6) sports games (e.g., football, tennis); (7) martial arts (e.g., boxing, karate).

For PMH outcomes, we differentiated three domains for the in-depth synthesis: (1) psychological and emotional well-being; (2) physical well-being; and (3) social well-being. We have chosen this categorisation based on general conceptualisations about PMH [5], conceptualisations about PMH in the context of PA (e.g., the relevance of the body) [7, 8], and the fit of contextual aspects of LTPA (e.g., social environment) to PMH domains (e.g., social well-being). Additionally, based on a current overview [6] and as introduced earlier in this paper, PMH outcome could be divided into experiential or reflective features (see Supplementary Section S8 for more information).

#### Synthesis of quantitative studies

In the first step, we conducted a multilevel meta-analysis considering multiple outcomes per study (supported by R-package metafor [54]) to display the effect sizes per outcome and quantify the mean effect sizes of each LTPA type. Due to the nascent and real-world nature of this field of research, we considered not only RCT’s to include the “best available evidence” [55]. We combined calculations for RCT’s and nRCT’s, and the experimental group’s pre-post mean differences compared to the control group’s pre-post mean difference was used. Additionally, we calculated single group studies separately by comparing pre-post mean group difference of the one group. We used Hedges’ g as the effect size measure, a precise measure for standardized mean differences (SMD), especially in small sample sizes [56]. Cohen’s convention provides an orientation for interpreting the magnitude of the effects (0.2 small, 0.5 medium, 0.8 large) [57]. Two similar studies are sufficient to calculate a meta-analysis [58]; however, in this case of very low numbers of studies, heterogeneity metrics of the meta-analysis are unreliable and provide no useful information about true heterogeneity [59]. When only one study per LTPA type, or only one study within a LTPA type with or without control group, was available, the SMD per study, respectively outcome, was calculated and included in the forest plot too, but no pooled effect sizes were calculated. Second, the effects found per LTPA type are further narratively synthesised. In doing so, PMH is differentiated into their three domains (psychological and emotional, physical, and social well-being). Within each PMH domain, experiential features (affective aspects) and reflective features (cognitive and evaluative aspects) are further differentiated [6]. The PMH outcomes of the included studies were classified independently into the PMH domains and features by two authors (LL, JS). Discrepancies were discussed and resolved.

#### Synthesis of qualitative studies

In addition to the quantitatively measured effects of LTPA on PMH, two authors (LL, AF) mapped findings from qualitative studies through a thematic network analysis based on Attride-Stirling [60], an approach also used in systematic reviews (e.g., John et al. [61]). This approach combines thematic analysis and thematic networks by organising and structuring the themes identified in a coherent representation. In doing so, three types of themes are differentiated, which we applied as follows: (1) Basic themes from the original studies, (2) organizing themes, comprising of clustered basic themes, and (3) global themes, comprising of multiple organizing themes [60].

#### Mixed-methods synthesis

In the last step, we combined the separate syntheses by complementing insights from both methodological streams [40–42]. As we focus on LTPA types, the combination of both syntheses was conducted per LTPA type. This integration of quantitative and qualitative evidence allows to “explore, contextualize, or explain the findings of the other type of evidence” and “to produce an overall configured analysis” (Stern et al. [41], p. 125). Consequently, for our review, the effects found from quantitative studies were enriched by insights from qualitative studies per LTPA type to better understand how and why (i.e. mechanisms) respective LTPA types are related to PMH outcomes.

## Results

### Study selection

**Fig. 1:**
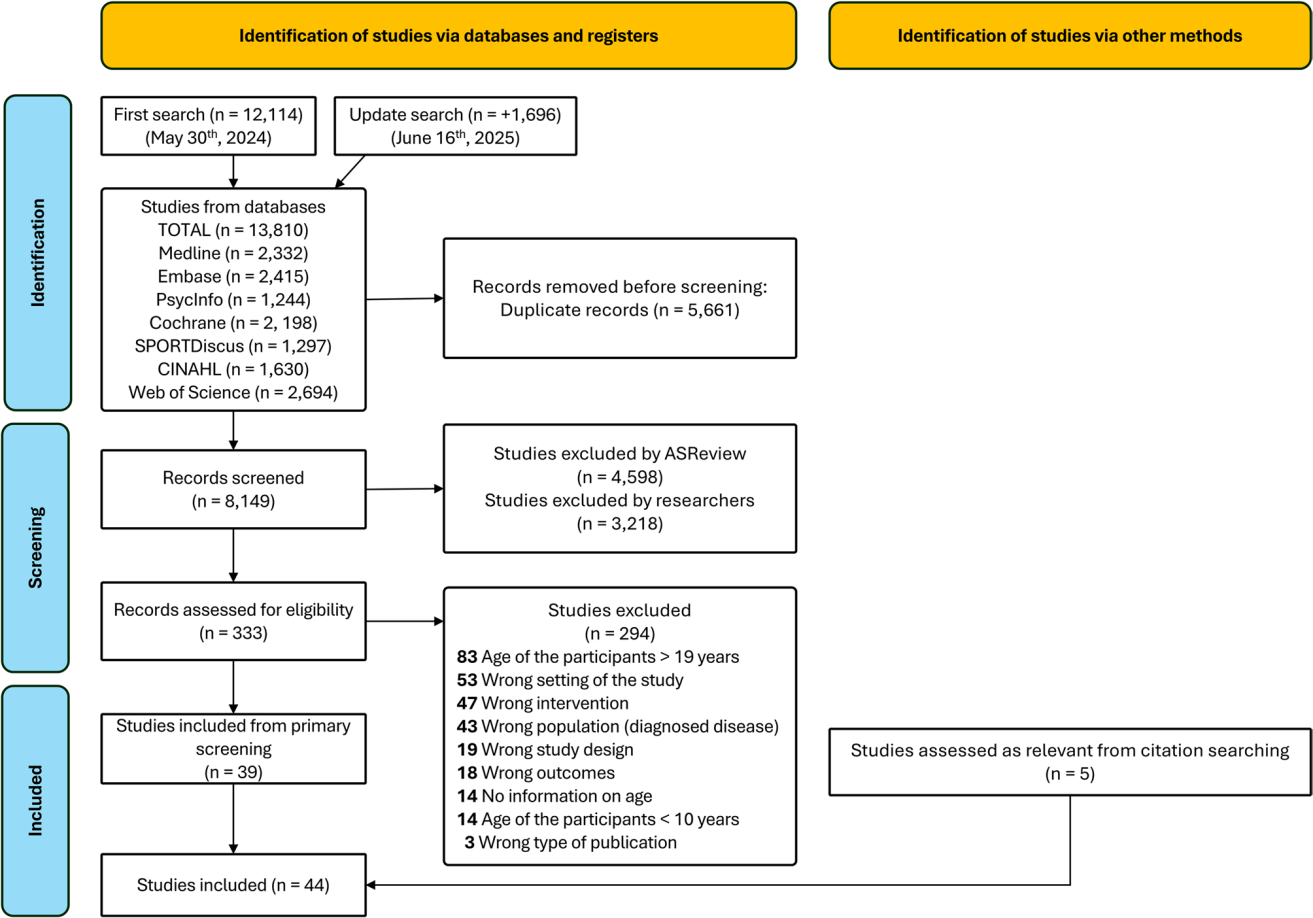
PRISMA flow diagram

From the initial 8,149 studies identified, 39 were included in the review. Five additional studies were included through citation searching, 44 in total (cf. PRISMA flow chart in Fig. 1). Most prominent reasons for exclusion were related to age (e.g., older than 19 years), the setting of the intervention (e.g., during mandatory school), intervention set-up (e.g., multiple types of LTPAs or intervention with LTPA and nutrition), and population (e.g., adolescents with obesity or diagnosed mental illness).

### Study characteristics

The 44 studies consisted of total *n* = 5,983 participants (female *n* = 3,669, male *n* = 2,130, information on sex not available *n* = 184) from 18 countries. 22 studies each reported from the general population and from at-risk populations as defined by the included studies (e.g., low socioeconomic status only, living in residential care centres, identified as having low mental health but not diagnosed as mentally ill). We included 24 quantitative studies, 11 qualitative studies, and 9 mixed-methods studies. From the 9 mixed-methods studies, 3 studies were integrated in both the qualitative and quantitative synthesis, 3 studies in the qualitative synthesis only, and 3 in quantitative synthesis only. We identified 18 different LTPA types in the 44 included studies (see Supplementary Table S9 for details on studies).

### Study quality

Qualitative studies, quantitative randomised studies (i.e. RCTs), and quantitative non-randomised studies (longitudinal studies without and with a non-randomised control group) were assessed via MMAT [50]: For mixed-methods studies, we separately performed a qualitative and quantitative assessment. A summative score of the criteria is discouraged; rather a description of the criteria is suggested to assess an overall estimation of the studies’ quality [50].

Studies’ quality was mixed (see Supplementary Table S10): While some qualitative studies were graded high quality, low quality tended to be observed in the qualitative part of mixed-methods studies. No quality assessment was possible for the qualitative part of one mixed-methods study [62]. Most common issues were related to the blinding process (for randomized quantitative studies) and accounting for potential confounders (for non-randomized quantitative studies).

### Study exclusion

Four studies were excluded from the synthesis: One study [63] was highly contradictory in reporting their findings, and no clarification was possible because the authors did not answer our inquiry. In one mixed-methods study [64], the qualitative part did not provide a clear description of the sample, and the quantitative part did not fulfil any of the relevant quality criteria, indicating a substantial risk of bias. The qualitative part of another mixed-methods study [62] was excluded because the qualitative data were unrelated to the research question. In one quantitative study [65], the descriptive data were not accessible, also not after the authors were contacted.

### Synthesis of the quantitative studies

**Fig. 2:**
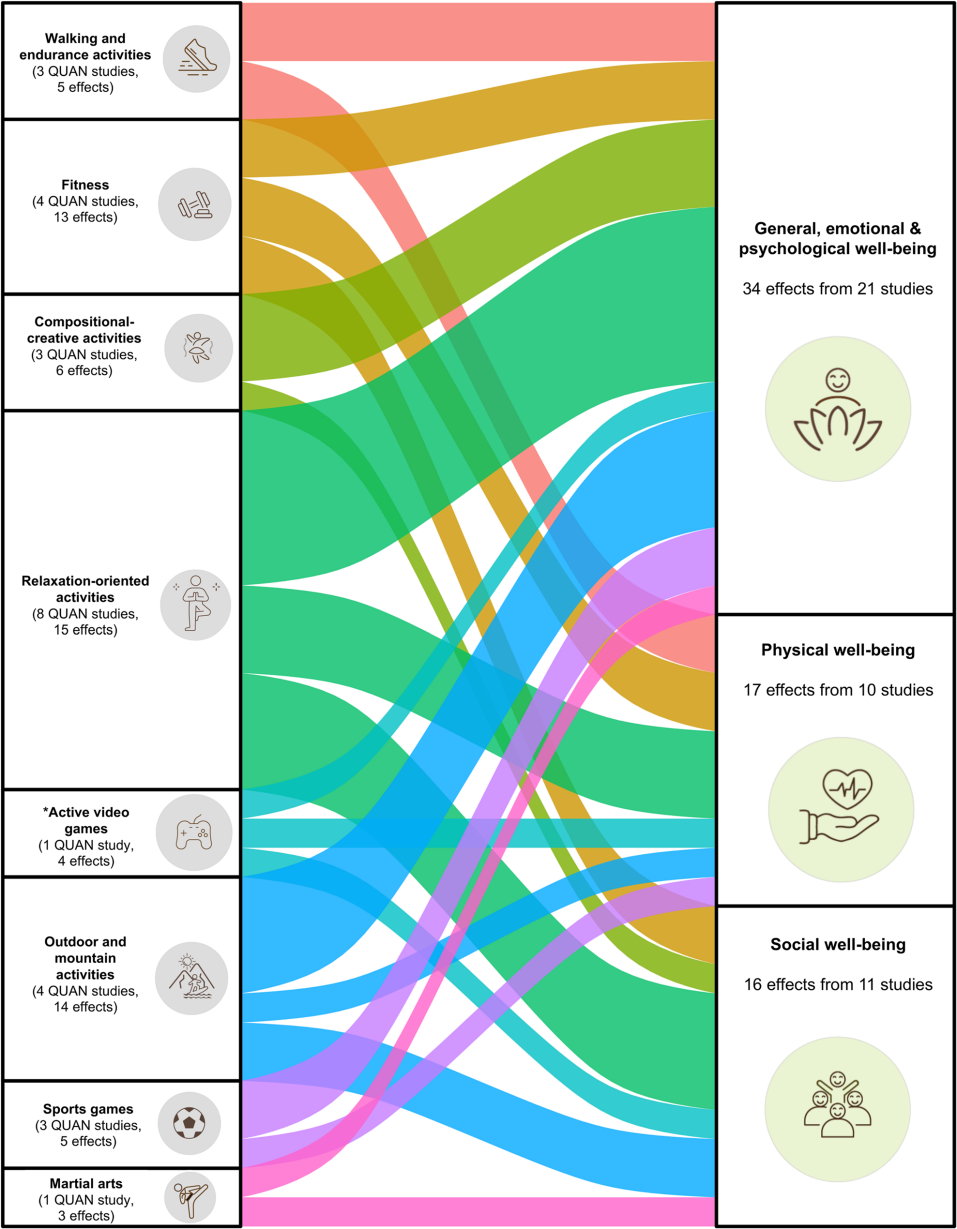
Sankey chart to display the number of studies and effects between LTPA types and domains of well-being. The thickness of the coloured bars indicates the number of studies between the respective LTPA type and well-being domain *The LTPA type of active video games was built additionally because the framework from Sudeck et al. (2013) does not include active video games

Figure 2 presents quantitative studies examining the relationship between different LTPA types and various PMH outcomes. There are between one and eight studies per LTPA type included, and they display effects on different domains of PMH: General, emotional and psychological aspects of well-being were most in focus (in 21 studies with 34 effects), while physical and social aspects of well-being occurred in a similar number of studies (physical well-being: 10 studies with 17 effects; social well-being: 11 studies with 17 effects). In sum, a differentiated picture was evident with studies from various LTPA types on – at least – two different domains of well-being.

**Fig. 3:**
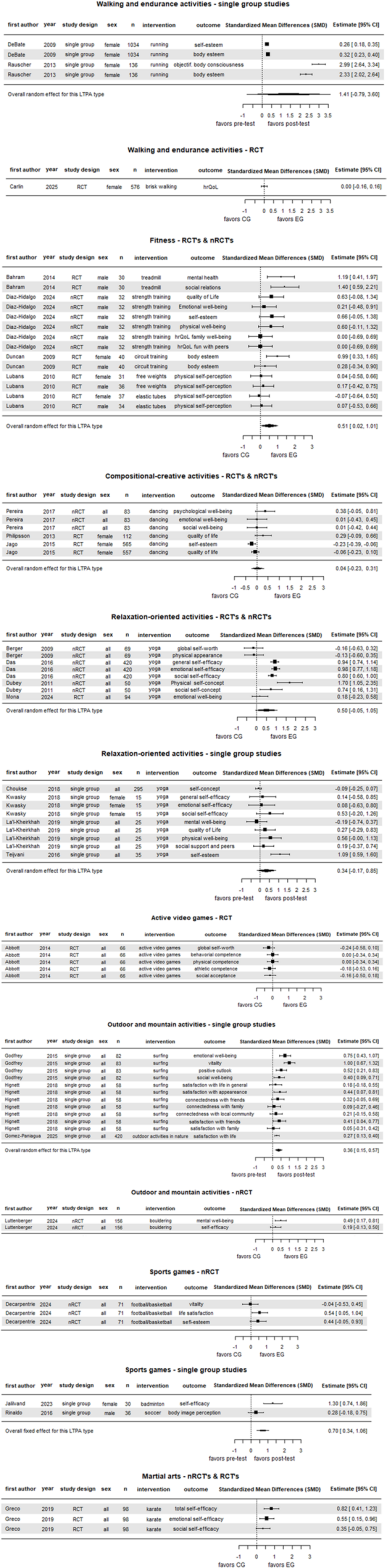
Forest plots of the meta-analyses, differentiated by LTPA types

Forest plots (Fig. 3) contain single effect sizes and meta-analytical mean effect size (Hedges’ g) per LTPA type (for more details about the meta-analysis, see Supplementary Section S11). Due to the relatively small number of studies per LTPA type, the confidence intervals (CI) of the mean effect sizes are relatively wide. Together with often small numbers of participants and, consequently, wide CIs in single effect sizes, all the values should be understood as an orientation and indicate a high uncertainty. Additionally, the different study designs (RCTs, nRCTs, single group studies) must be kept in mind when interpreting the findings.

**Fig. 4:**
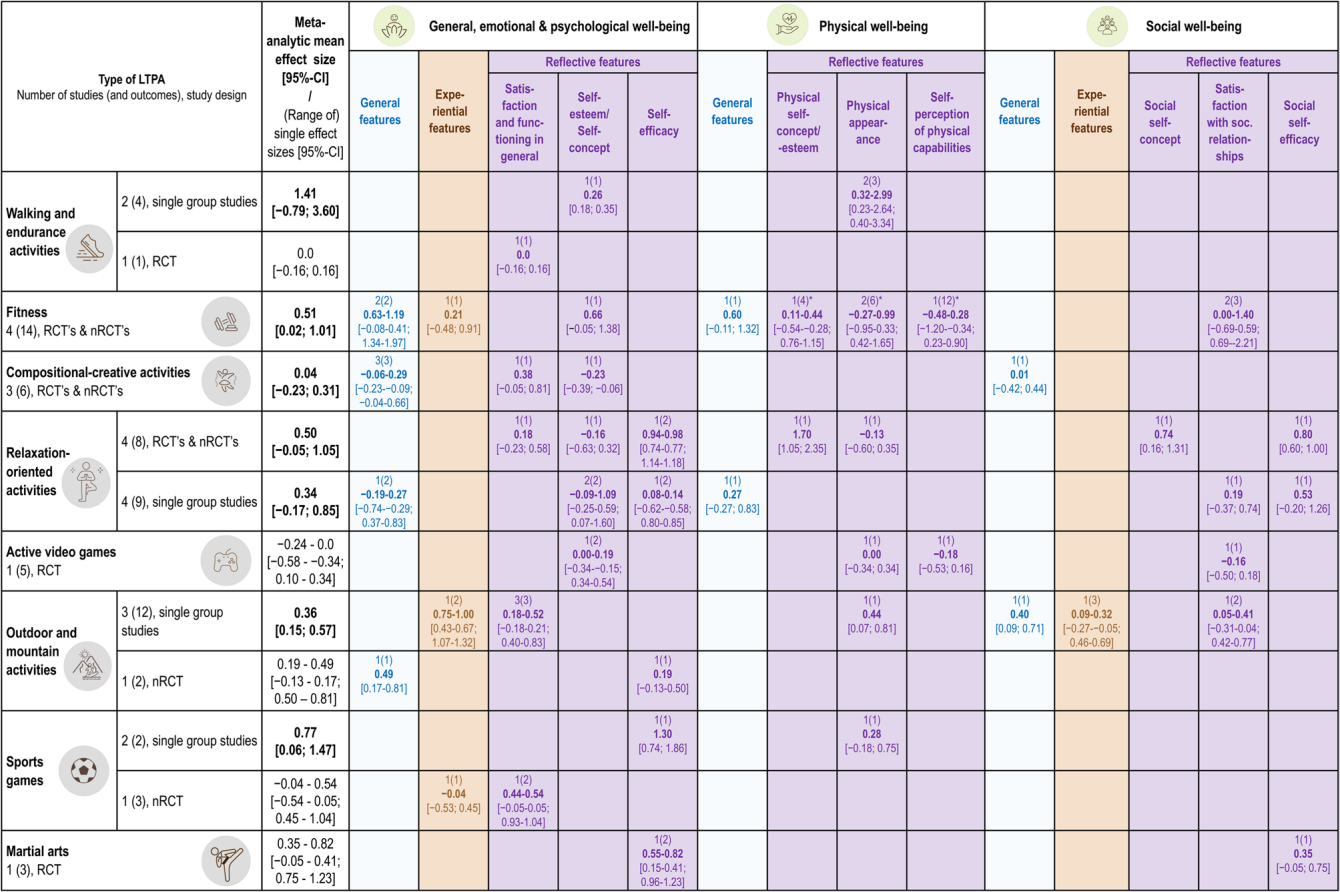
Meta-analytic effects and effects per mental health aspect Caption: In the coloured cells, the first number indicates the number of studies, in brackets the number of effects sizes and then the effect size with the 95%-CI. In the case of multiple effect sizes, the range of the effect sizes is displayed

Overall, the mean effect sizes from the meta-analyses are all above zero, ranging from 0.04 to 1.41. The 95%-CI remains positive in three analyses (fitness: RCT’s & nRCT’s; outdoor and mountain activities: single group studies; sports games: single group studies), indicating a robust positive effect, while the 95%-CI crosses zero in four analyses (walking and endurance activities: single group studies; compositional-creative activities: RCT’s & nRCT’s; relaxation-oriented activities: RCT’s & nRCT’s and single group studies), indicating no robust positive effects. No meta-analyses were possible due to only one study per LTPA type or per study design for the RCT in walking and endurance activities, the RCT in active video games, the RCT in outdoor and mountain activities, the nRCT in sports games, and the RCT in martial arts. Thus, the single effect sizes with 95%-CI are reported. Figure 4 combines the meta-analytical effect sizes, or the single effect sizes with their range, per LTPA with the further differentiation of the PMH domains and features (see Supplementary Section S8 for further information about the PMH classification).

For walking and endurance activities, the overall effect size of the single group studies (1.41 [−0.79; 3.60]) implies a generally positive effect with high uncertainty. Both single group studies [66, 67] reveal a positive effect on a reflective feature of physical well-being: physical appearance (0.32–2.99 [0.23–2.64; 0.40–3.34]). Furthermore, another reflective feature, within the general, emotional & psychological well-being domain, seems to be promoted by this LTPA type: self-esteem (0.26 [0.18; 0.35]) [66]. However, the RCT shows no effect (0.0 [− 0.16; 0.16]) for satisfaction and functioning in general [68].

For fitness, results from total two nRCTs [69, 70] and two RCTs [71, 72] indicate an overall medium positive effect on PMH (0.51 [0.02; 1.01]). Looking within the domains and features of PMH, there is no consistent positive effect considering the CIs, but positive small to large effects in every PMH subcategory except some reflective features in the physical well-being. General features are most likely to benefit from fitness activities (for general, emotional & psychological well-being: 0.63–1.19 [−0.08-0.41; 1.34-1.97] [69, 71]; for physical well-being: 0.60 [0.11; 1.32] [69]).

In compositional-creative activities, the two RCTs [73, 74] and the nRCT [75] suggest no effect on PMH in general (0.04 [0.23; 0.31]. Considering the CIs, no single effect size demonstrated a consistently positive effect, but one showed a small negative effect on a reflective feature in the general, emotional & psychological well-being domain (i.e. self-esteem −0.23 [−0.39; −0.06]) [74].

For relaxation-oriented activities, two separate meta-analyses were calculated due to the heterogeneous study designs (i.e. with and without control groups). For the RCT [76] and nRCTs [77–79], there is an overall medium effect size with a high uncertainty (0.50 [−0.05; 1.05]). For single group studies [80–83], the mean effect sizes are a little bit smaller with a wide CI too (0.34[−0.17; 0.85]). For some reflective features, especially for certain aspects of the self, this LTPA type seems beneficial.

For active video games, the only RCT for this LTPA type [84] shows no substantial effects in any of the outcomes measured, ranging from − 0.24 to 0.0 [0.5- −0.34; 0.10-0.34].

For outdoor and mountain activities, the mean effect size from the three single group studies [62, 85, 86] indicates a positive small to medium value (0.36 [0.15; 0.57]). There are only outcomes from one study for a respective PMH feature within a PMH domain, except for the reflective feature within the general, emotional and psychological well-being domain: small to medium effect sizes with high uncertainty (0.18-0.52 [−0.18-0.21; 0.55-0.83]). Large effect sizes occur in experiential features within the general, emotional and psychological well-being domain (0.75-1.00 [0.43-0.67; 1.07-1.32]). The nRCT [87] demonstrate a robust positive effect on general features (0.49 [0.17-0.81]) but not on reflective features (0.19 [−0.13-0.50]) within the general, emotional and psychological well-being domain.

For sports games, two outcomes of two single group studies [88, 89] indicate an overall large positive effect (0.77 [0.06; 1.47]). The two outcomes relate to reflective features of PMH: a large effect is demonstrated for self-efficacy (1.30 [0.74; 1.86]) [89], while a small effect with high uncertainty is shown for physical appearance (0.28 [−0.18; 0.75]) [88]. The nRCT [90] displays in two of three outcomes medium positive effect sizes for reflective features, but only in one outcome (life satisfaction) in a consistent manner (95%-CI > 0).

For martial arts, the three outcomes from the only RCT [91] imply small to large positive effect sizes, with consistent positive CI’s for reflective features (i.e. self-efficacy) in the general, emotional, and psychological well-being domain (0.55-0.82 [0.15-0.41; 0.96-1.23]), but not consistently in the social well-being domain (0.35 [−0.05; 0.75]).

Looking independently of the LTPA type and from a PMH perspective, especially self-efficacy as a reflective feature seems to be well fostered in general. All the other categories within the PMH domains and features display no consistent positive effects. Consequently, the effects highly depend on the LTPA type and on the PMH outcome in focus. The findings are based on a low number of studies and the various study designs have to keep in mind.

### Synthesis of the qualitative studies

**Fig. 5:**
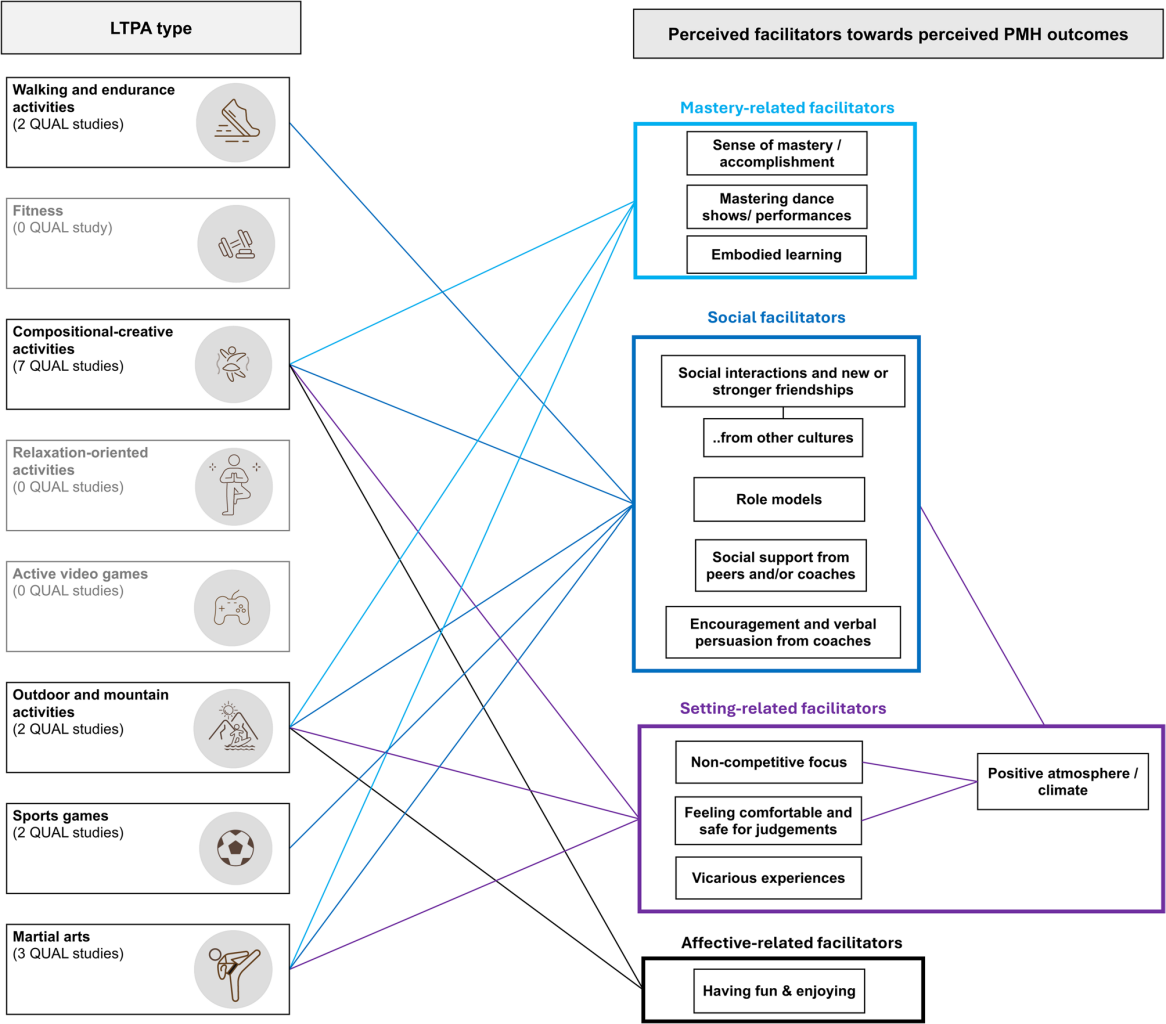
Synthesis of the qualitative synthesis Caption: lines between boxes indicate an association

We herein present participants’ subjective experiences in two global themes (see Fig. 5): (1) differentiation of the LTPA types and (2) facilitators towards perceived PMH outcomes. The LTPA types differentiation of Sudeck et al. [53] acted as organising themes, encompassing the specific LTPA types of the single studies, which act as the basic themes (left side of Fig. 5). We inductively mapped the perceived facilitators in four organising themes (right side of Fig. 5): (1) mastery-related facilitators, (2) social facilitators, (3) setting-related facilitators, and (4) affective-related facilitators. Mastery-related facilitators refer to intrapersonal aspects to master a specific task or exercise; social facilitators are located at the interpersonal level and refer to directly perceived support from other person(s). Setting-related facilitators refer to interpersonal aspects that are not directly linked to a person but rather to the perceived atmosphere and climate, and affective-related facilitators are linked to perceived emotions during the activity. Within these organising themes, there are specific facilitators, or so-called basic themes.

The 16 qualitative studies are divided into 5 LTPA types: two studies in walking and endurance activities [68, 92]; seven studies in compositional-creative activities [93–99]; two studies in outdoor and mountain activities [85, 100]; two studies in sports games [101, 102]; and three studies in martial arts [103–105]. We did not identify qualitative studies for the other LTPA types (see left side of Fig. 5).

Overall, direct perceived PMH outcomes from LTPA are reported, as well as perceived outcomes via perceived facilitators (see Supplementary Figure S12 for perceived outcomes and single studies outcomes). Thus, we can conclude that the practice of LTPA was directly related to perceived PMH outcomes and certain facilitators were perceived as supportive for a PMH outcome. We focus on the perceived facilitators to gain more insights from the unique qualitative data on *why* certain LTPA could affect PMH [41]: Social and mastery-related facilitators were the two most prominent ones reported in various LTPA types. Within there, *sense of mastery/accomplishment*, *social interactions and new or stronger friendships* and *social support from peers and/or coaches* were mentioned the most and across various LTPA types (see upper part in Supplementary Figure S12). Additionally, *feeling comfortable and safe for judgements* within setting-related facilitators indicates another important facilitator mentioned across different LTPA types. The group of setting-related facilitators has additionally another characteristic: *Positive atmosphere/climate* is a perceived consequence from other facilitators within supportive climate-related facilitators (i.e. *non-competitive focus*, *feeling comfortable and safe for judgements*) and also from social facilitators (i.e. *social interactions and new or stronger friendships*, *social support from peers and/or coaches*; see Supplementary Figure S12). Thus, a positive atmosphere/climate is related to other facilitators from the same domain as well as from another.

There were also patterns of facilitators depending on the LTPA type, such as those shared by compositional-creative activities and outdoor and mountain activities. For these two LTPA types, all groups of facilitators (mastery-related, social, setting-related, and affective-related) were involved. This interplay of facilitators was reported for these two LTPA types only. In contrast, only social facilitators were reported in walking and endurance activities and sports games. In every LTPA type, social facilitators were mentioned. In sum, there are facilitators and configurations depending on the LTPA type besides some rather general perceived facilitators (e.g., social support, sense of mastery).

### Mixed-methods meta-synthesis

**Fig. 6:**
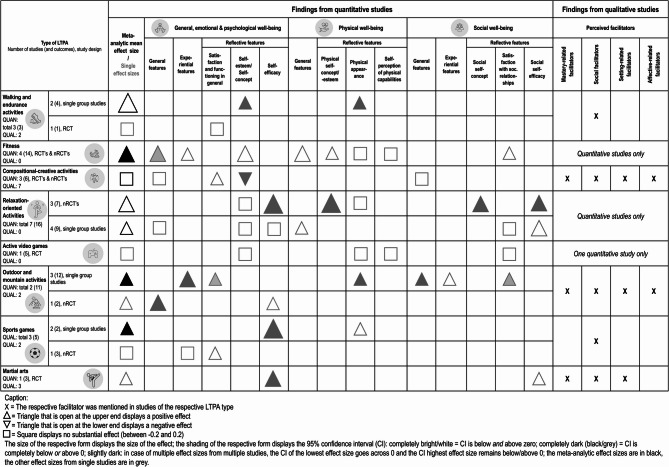
Mixed-methods synthesis

The effects found in quantitative studies (“that-knowledge” according to Sandelowski et al. [42]) were contextualised by perceived facilitators reported in the qualitative studies (“why-knowledge” according to Sandelowski et al. [42]). These complementary findings are shown and summarised in Fig. 6.

For the LTPA types of fitness, relaxation-oriented activities, and active video games are only quantitative studies available, and thus, a mixed-methods meta-synthesis is not possible. For walking and endurance activities, the large but uncertain meta-analytical effect from single group studies on PMH, based on effects on physical appearance and self-esteem/concept, appears to be facilitated through social factors (i.e. social support from peers; see supplementary figure S12) that helped participants to keep running. However, this effect was not found in the RCT, and this facilitator may not have been effective there. For compositional-creative activities, quantitative studies revealed no consistent positive effects. However, the seven qualitative studies reported a variety and configuration of facilitators for perceived mental health benefits, with mastery-related and social facilitators mentioned in all the studies. Consequently, it remains unclear whether the facilitators cannot contribute to quantitatively measured positive effects, or whether study-related features differed between the quantitative and qualitative investigations. For outdoor and mountain activities, the meta-analytical positive medium effect size in single group studies and small to moderate effect size from the nRCT seem to be mediated particularly by social and setting-related facilitators (see supplementary figure S12). For sports games, the meta-analytical medium to large positive effect in single group studies appeared to be facilitated through social aspects (role models and social support, see supplementary figure S12). However, the nRCT study indicate that this facilitator might work for reflective features of PMH, but not for experiential features. For martial arts, the small to medium positive effects for self-efficacy aspects is complemented by mastery-related (i.e. sense of mastery), social (i.e. encouragement and verbal persuasion from coaches), and setting-related (i.e. vicarious experiences) facilitators.

In sum, complementing quantitative and qualitative findings offered new insights on what could work and why. The greater number of perceived facilitators did not indicate greater quantitative effects. The important role of social facilitators also becomes evident by contextualising positive quantitative effects with only this facilitator (for walking and endurance activities and sports games). Configurations of different facilitators, depending on the LTPA type, provide possible explanations for the mechanisms and effectiveness of an activity.

## Discussion

This systematic review and meta-analysis synthesised literature of specific LTPA types on PMH among adolescents from multiple study designs. Overall, the findings indicate that there are rather effects on PMH depending on the LTPA type than general effects, supported by a configuration of different facilitators with social facilitators as the most dominant one. According to the findings of the quantitative studies, we found consistent positive effects from the meta-analyses for fitness (RCT’s & nRCT’s), outdoor and mountain activities (single group studies), and sports games (single group studies). Across the various LTPA types, there is a high heterogeneity and thus, a high uncertainty evident. From a PMH perspective, no specific outcome appears to be fostered by all LTPA types. Self-efficacy as a reflective feature displayed as the only PMH outcome with consistent positive effects across multiple LTPA types. However, the results must be interpreted cautiously due to the low numbers of studies per LTPA type and the different study designs. According to the findings of the qualitative studies, mastery-related, social, setting-related, and affective-related facilitators acted as perceived mediators between LTPA types and perceived PMH outcomes. While social facilitators play a role in all LTPA types, configurations of facilitators depending on the LTPA type become evident. Compositional-creative activities and outdoor and mountain activities show a similar combination of facilitators, indicating similar characteristics of these two LTPA types. According to the mixed-methods synthesis, the number of facilitators does not relate to the size of the quantitative effect; rather, the unique constellation of facilitators offers insights on why a given LTPA activity could contribute to PMH. By looking at PA from a perspective considering qualitative and contextual characteristics [21], such as the LTPA type, and its effects on mental health [20], this review offers new insights on how different LTPA types are experienced, facilitated and related to PMH outcomes.

### Relating the findings to existing literature

Our findings complement existing reviews that often focus on negative aspects of mental health (e.g., depression) and do not differentiate the type of LTPA but, for instance, only between team and individual sports or aerobic and anaerobic activities [19, 29–32]. Comparing team sports with sports games, findings from our review show positive outcomes for reflective features, especially in the general, emotional, and psychological well-being domain. This fits to general mental health benefits from team sports reported in other reviews (e.g., Rodriguez-Ayllon et al. [19]). The review by Säfvenbom et al. [34] similarly shows positive mental and social effects of lifestyle sports, such as surfing, which represent outdoor and mountain activities in our review. However, they do not further differentiate the LTPA types and PMH domains. The findings from another review about resistance training on aspects of the self in youth [35] are in line with our findings for fitness regarding improvement in self-esteem/-worth and no consistently positive changes in physical appearance. However, other findings from Collins et al. [35] are not congruent with our findings (e.g., about physical self-worth/esteem). For compositional-creative activities (e.g., dancing), positive effects reported in other reviews about dancing, especially for body perception and self-esteem [36–38], cannot be confirmed for quantitative studies in our review, but also due to fact that body perception was not investigated in quantitative studies of our review.

Looking at the perceived facilitators, our findings align with other studies, concepts and theories as follows: Given the differentiation of (neuro-)biological, psychological, social, behavioural, and environmental processes to explain the relationship between an activity and mental health outcomes [22–24], in our review the facilitators are identified via interviews and observations and, thus, relate to psychological and social, or psychosocial processes. Within psychosocial facilitators, the most prominent theory reported in existing literature is the Self-Determination Theory (SDT) [106], with its basic psychological needs of competence, autonomy, and relatedness for enhanced motivation and well-being. Mastery-related and social facilitators from our review are closely related to competence and relatedness from the SDT, while autonomy was not reported in our included studies. Similar to our review, the mediation of these basic psychological needs between PA and PMH has been confirmed at the general level of LTPA and PMH both quantitatively [107] and qualitatively [108–110]. The findings of the qualitative studies in our review suggest that mastery-related and social facilitators do not work for every LTPA type in the same manner. In addition, general or task-related (e.g., exercise) self-efficacy is often used as a mediator between PA and mental health outcomes [24, 111], and mastery-related facilitators can be seen close to aspects of self-efficacy. However, included quantitative studies also used self-efficacy as an outcome. Our findings on setting-related facilitators align with existing literature on perceived climate [112] or the coach’s role in creating a climate for PMH experiences [113]. The importance of feeling comfortable and safe for judgements as a setting-related facilitator, found in our review, is also discussed in a larger context about safe environments for practising LTPA [114], addressed in SAAFE teaching principles (Supportive, Active, Autonomous, Fair, Enjoyable) for organised PA [115]. Affective-related facilitators indicate that LTPA experiences should not only be safe but also enjoyable for positive outcomes, a finding also reported elsewhere [110, 113, 116] that indicates, ultimately, the interplay of facilitators.

### Strengths and limitations

A key strength of this review lies in its effort to integrate emerging theoretical perspectives by incorporating qualitative and contextual aspects of PA [20, 28], drawing on categorisations from exercise psychology and health promotion [53] to address the still-evolving classification of LTPA types. And we applied a broad, theory-informed understanding of positive mental health (PMH) [1, 6, 8] and acknowledged physical and social aspects of well-being as central dimensions in the context of PA besides psychological and emotional well-being. In the meta-analysis, we synthesised diverse longitudinal study designs to reflect the best available evidence in this developing field while adhering to established methodological standards [55].

The differentiated approach chosen in this mixed-methods review has advantages by segmenting the LTPA types as well as the PMH outcomes, but also has some limitations: (1) Findings of this review are dependent on the existing original studies and represent the published evidence of what is already known, with a currently limited number of studies for certain LTPA types and PMH outcomes. This limits the statistical generalizability of the findings. (2) By focusing on the LTPA type, other contextual factors of PA related to PMH outcomes are not considered (e.g., social or physical environment) [20]. However, an overlap of contextual factors might also be possible [20], as introduced earlier in this review (the LTPA type football is linked to specific social and physical environments). Additionally, inter-individual differences in the preference for LTPA types are also known [117]. Thus, the same LTPA type does not automatically evoke the same effects on PMH in every individual. (3) Different study designs make direct comparisons between studies challenging. For example, Vella et al. [20] describe that the effects found in their umbrella review can depend on the type of the control group. This phenomenon is observed for certain outcomes in two studies of our review [69, 75]: the experimental group reached non-significant effects for certain outcomes because of an increase in the active control group. However, mixed-methods reviews aim to synthesize evidence from different methodologies and study designs. This is why this approach was consistently followed, and multiple checklists (PRISMA, ENTREQ) are included to guide the synthesis process and to consider carefully the different study designs. (4) The populations included are general and at-risk adolescents. We found no systematic differences in the findings between these two groups. Thus, we did not report separate syntheses based on population. Similarly, we did not separate analyses per sex or gender because of the already highly differentiated analyses with LTPA types and PMH outcomes.

## Conclusions

This mixed-methods systematic review and meta-analysis highlights the effects of various LTPA types on different aspects of positive mental health (PMH) in adolescents. Findings from longitudinal quantitative and qualitative studies indicate that the impact of LTPA depends on the type of activity and the specific PMH aspect considered. While no single LTPA universally enhances PMH, self-efficacy appears to be consistently promoted, with social facilitators representing the most prominent psychosocial mechanism. This review and meta-analysis advance the understanding of the LTPA-PMH interplay and provide ecologically valid insights into which type of LTPA could be implemented in specific contexts to promote targeted PMH outcomes. Our findings extend the existing knowledge by indicating that no LTPA type generally fosters mental health, but rather indicate the necessity of considering a differentiated perspective on both - PA with its activity types and mental health with its various dimensions. Future research can integrate our differentiated findings when investigating the relationship between PA and mental health by considering the activity type and mental health outcome in focus. For practical implications, these insights can serve as a basis both at an individual level, e.g. in tailoring PA counselling (matching an LTPA type and its psychosocial processes to the individual’s conditions) [118], and at a policy level, e.g. in promoting evidence-informed health promotion and policy-making (matching an LTPA type and its psychosocial processes to local and cultural conditions). However, the limited number of studies per LTPA type, PMH aspect, and study design highlights the need for further research to strengthen these findings regarding the effects and possible mechanisms.

## Supplementary Information

Supplementary Material 1.

## Data Availability

Not applicable, all data can be found in the original articles.

## Abbreviations

MMAT: Mixed methods appraisal tool
nRCT: Non-randomised controlled trial
LTPA: Leisure-time physical activity
PA: Physical activity
PMH: Positive mental health
RCT: Randomised controlled trial
SDT: Self-Determination Theory
WHO: World Health Organisation
