# Supplementary material for: The effects of different types of leisure-time physical activity on positive mental health among adolescents: a mixed-methods systematic review and meta-analysis
Source: Int J Behav Nutr Phys Act. 2025 Oct 7;22:123. doi: 10.1186/s12966-025-01834-4 (PMC12506210; doi:10.1186/s12966-025-01834-4)
Supplement: Supplementary file 1 — Supplementary Material 1. [file 12966_2025_1834_MOESM1_ESM.docx]

**Supplementary File**

**Table of contents**

[Supplementary Table S1: PRISMA 2020 Checklist 2](#_Toc204095387)

[Supplementary Table S2: ENTREQ Checklist 6](#_Toc204095388)

[Supplementary Section S3: Deviation of the protocol 8](#_Toc204095389)

[Supplementary Section S4: Decision tree for including and excluding relevant studies 9](#_Toc204095390)

[Supplementary Table S5: Overview of databases & search results 12](#_Toc204095391)

[Supplementary Section S6: Detailed information about title-abstract-screening with ASReview 23](#_Toc204095392)

[Supplementary Table S7: Extended Mixed-Methods Appraisal Tool (MMAT) 24](#_Toc204095393)

[Supplementary Section S8: Information about the classification of the PMH outcomes 28](#_Toc204095394)

[Supplementary Table S9: Detailed information per study 29](#_Toc204095395)

[Supplementary Table S10: Quality Assessment of included studies 51](#_Toc204095396)

[Supplementary Section S11: Detailed information about the meta-analysis 57](#_Toc204095397)

[Supplementary Figure S12: Detailed synthesis of the qualitative studies with all themes 58](#_Toc204095398)

# Supplementary Table S1: PRISMA 2020 Checklist

| **Section and Topic** | **Item #** | **Checklist item** | **Location where item is reported** |
| --- | --- | --- | --- |
| **TITLE** | | |  |
| Title | 1 | Identify the report as a systematic review. | Title |
| **ABSTRACT** | | |  |
| Abstract | 2 | See the PRISMA 2020 for Abstracts checklist. | Abstract written based on this checklist |
| **INTRODUCTION** | | |  |
| Rationale | 3 | Describe the rationale for the review in the context of existing knowledge. | Start of the background |
| Objectives | 4 | Provide an explicit statement of the objective(s) or question(s) the review addresses. | Last section of the background |
| **METHODS** | | |  |
| Eligibility criteria | 5 | Specify the inclusion and exclusion criteria for the review and how studies were grouped for the syntheses. | Subchapter Eligibility criteria; Suppl. Table S4 |
| Information sources | 6 | Specify all databases, registers, websites, organisations, reference lists and other sources searched or consulted to identify studies. Specify the date when each source was last searched or consulted. | Subchapter Information sources and search strategy |
| Search strategy | 7 | Present the full search strategies for all databases, registers and websites, including any filters and limits used. | Suppl. Table S5 |
| Selection process | 8 | Specify the methods used to decide whether a study met the inclusion criteria of the review, including how many reviewers screened each record and each report retrieved, whether they worked independently, and if applicable, details of automation tools used in the process. | Subchapter Screening process, Supplementary Section S6 |
| Data collection process | 9 | Specify the methods used to collect data from reports, including how many reviewers collected data from each report, whether they worked independently, any processes for obtaining or confirming data from study investigators, and if applicable, details of automation tools used in the process. | Subchapter Data extraction |
| Data items | 10a | List and define all outcomes for which data were sought. Specify whether all results that were compatible with each outcome domain in each study were sought (e.g. for all measures, time points, analyses), and if not, the methods used to decide which results to collect. | Subchapter Data extraction, Suppl. Table S4 |
|  | 10b | List and define all other variables for which data were sought (e.g. participant and intervention characteristics, funding sources). Describe any assumptions made about any missing or unclear information. | Subchapter Data extraction, Suppl. Table S4 |
| Study risk of bias assessment | 11 | Specify the methods used to assess risk of bias in the included studies, including details of the tool(s) used, how many reviewers assessed each study and whether they worked independently, and if applicable, details of automation tools used in the process. | Subchapter Quality Assessment |
| Effect measures | 12 | Specify for each outcome the effect measure(s) (e.g. risk ratio, mean difference) used in the synthesis or presentation of results. | First part of the subchapter Synthesis of quantitative studies, subchapter Synthesis of qualitative studies |
| Synthesis methods | 13a | Describe the processes used to decide which studies were eligible for each synthesis (e.g. tabulating the study intervention characteristics and comparing against the planned groups for each synthesis (item #5)). | Whole subchapter Data synthesis, Suppl. Table S4 |
|  | 13b | Describe any methods required to prepare the data for presentation or synthesis, such as handling of missing summary statistics, or data conversions. | Subchapters Synthesis of quantitative studies, Synthesis of qualitative studies, Mixed-methods synthesis |
|  | 13c | Describe any methods used to tabulate or visually display results of individual studies and syntheses. | Subchapters Synthesis of qualitative studies, beginning of the results’ subchapter Synthesis of the quantitative studies |
|  | 13d | Describe any methods used to synthesize results and provide a rationale for the choice(s). If meta-analysis was performed, describe the model(s), method(s) to identify the presence and extent of statistical heterogeneity, and software package(s) used. | Subchapters Synthesis of quantitative studies, Synthesis of qualitative studies, Mixed-methods synthesis |
|  | 13e | Describe any methods used to explore possible causes of heterogeneity among study results (e.g. subgroup analysis, meta-regression). | Subchapter Synthesis of quantitative studies, Suppl. Section S9 |
|  | 13f | Describe any sensitivity analyses conducted to assess robustness of the synthesized results. | Suppl. Section S11 |
| Reporting bias assessment | 14 | Describe any methods used to assess risk of bias due to missing results in a synthesis (arising from reporting biases). | Line 255-261 |
| Certainty assessment | 15 | Describe any methods used to assess certainty (or confidence) in the body of evidence for an outcome. | Suppl. Section S11 |
| **RESULTS** | | |  |
| Study selection | 16a | Describe the results of the search and selection process, from the number of records identified in the search to the number of studies included in the review, ideally using a flow diagram. | Figure 1 |
|  | 16b | Cite studies that might appear to meet the inclusion criteria, but which were excluded, and explain why they were excluded. | Subchapter Study exclusion |
| Study characteristics | 17 | Cite each included study and present its characteristics. | Suppl. Table S9 |
| Risk of bias in studies | 18 | Present assessments of risk of bias for each included study. | Suppl. Table S10 |
| Results of individual studies | 19 | For all outcomes, present, for each study: (a) summary statistics for each group (where appropriate) and (b) an effect estimate and its precision (e.g. confidence/credible interval), ideally using structured tables or plots. | Figure 2, Suppl. Figure S12 |
| Results of syntheses | 20a | For each synthesis, briefly summarise the characteristics and risk of bias among contributing studies. | Results’ subchapters Synthesis of quantitative the studies,Synthesis of qualitative the studies, Mixed-methods meta-synthesis |
|  | 20b | Present results of all statistical syntheses conducted. If meta-analysis was done, present for each the summary estimate and its precision (e.g. confidence/credible interval) and measures of statistical heterogeneity. If comparing groups, describe the direction of the effect. | Figure 2 & subchapters Synthesis of quantitative the studies |
|  | 20c | Present results of all investigations of possible causes of heterogeneity among study results. | Suppl. Section S11 |
|  | 20d | Present results of all sensitivity analyses conducted to assess the robustness of the synthesized results. | Suppl. Section S11 |
| Reporting biases | 21 | Present assessments of risk of bias due to missing results (arising from reporting biases) for each synthesis assessed. | NA |
| Certainty of evidence | 22 | Present assessments of certainty (or confidence) in the body of evidence for each outcome assessed. | Suppl. Section S11 |
| **DISCUSSION** | | |  |
| Discussion | 23a | Provide a general interpretation of the results in the context of other evidence. | Subchapter Relating the findings to existing literature |
|  | 23b | Discuss any limitations of the evidence included in the review. | Second part in Subchapter Strengths and limitations |
|  | 23c | Discuss any limitations of the review processes used. | Second part in Subchapter Strengths and limitations |
|  | 23d | Discuss implications of the results for practice, policy, and future research. | Subchapter Conclusions |
| **OTHER INFORMATION** | | |  |
| Registration and protocol | 24a | Provide registration information for the review, including register name and registration number, or state that the review was not registered. | Subchapter Design, protocol and registration |
|  | 24b | Indicate where the review protocol can be accessed, or state that a protocol was not prepared. | Subchapter Design, protocol and registration |
|  | 24c | Describe and explain any amendments to information provided at registration or in the protocol. | Subchapter Design, protocol and registration& Suppl. Section S3 |
| Support | 25 | Describe sources of financial or non-financial support for the review, and the role of the funders or sponsors in the review. | See in Declarations |
| Competing interests | 26 | Declare any competing interests of review authors. | See in Declarations |
| Availability of data, code and other materials | 27 | Report which of the following are publicly available and where they can be found: template data collection forms; data extracted from included studies; data used for all analyses; analytic code; any other materials used in the review. | See supplementary material |

*From:* Page MJ, McKenzie JE, Bossuyt PM, Boutron I, Hoffmann TC, Mulrow CD, et al. The PRISMA 2020 statement: an updated guideline for reporting systematic reviews. BMJ 2021;372:n71. doi: 10.1136/bmj.n71

# Supplementary Table S2: ENTREQ Checklist

| **No** | **Item** | **Guide and Description** | **yes/no** | **Location where item is reported** |
| --- | --- | --- | --- | --- |
| 1 | Aim | State the research question the synthesis addresses. | yes | Last section of the background |
| 2 | Synthesis methodology | Identify the synthesis methodology or theoretical framework underpinning the synthesis. Describe the rationale for the choice of methodology (e.g., meta-ethnography, thematic synthesis, grounded theory synthesis, etc.). | yes | Subchapters Synthesis of quantitative studies, Synthesis of qualitative studies, Mixed-methods synthesis |
| 3 | Approach to searching | Indicate whether the search was pre-planned (comprehensive) or iterative to seek all available concepts until theoretical saturation is achieved. | yes | Subchapter Information sources and search strategy |
| 4 | Inclusion criteria | Specify the inclusion/exclusion criteria (e.g., population, language, publication year, study type). | yes | Subchapter Eligibility criteria; Suppl. Table S4 |
| 5 | Data sources | Describe the information sources used (e.g., electronic databases, gray literature, experts, etc.) and provide the rationale for using the sources. | yes | Subchapter Information sources and search strategy |
| 6 | Electronic Search strategy | Describe the literature search, including electronic search strategies, filters for qualitative research, and search limits. | yes | Suppl. Table S5 |
| 7 | Study screening methods | Describe the process of study screening and sifting (e.g., title, abstract, and full-text review, number of independent reviewers). | yes | Subchapter Screening process, Supp. Section S6 |
| 8 | Study characteristics | Present the characteristics of included studies (e.g., year of publication, country, population, data collection methods, analysis, etc.). | yes | Subchapter study characteristics & Suppl. Table S7 |
| 9 | Study selection results | Identify the number of studies screened and reasons for study exclusion. Provide details for modifications to the research question or theory development. | yes | Subchapter Study selection &  Figure 1 |
| 10 | Rationale for appraisal | Describe the rationale and approach used to appraise included studies or findings (e.g., validity, robustness, transparency). | yes | Subchapter Quality Assessment |
| 11 | Appraisal items | State the tools, frameworks, and criteria used to appraise studies (e.g., CASP, COREQ, reviewer-developed tools). | yes | Subchapter Quality Assessment & Suppl. Table S7 |
| 12 | Appraisal process | Indicate whether appraisal was conducted independently by multiple reviewers and if consensus was required. | yes | Subchapter Quality Assessment |
| 13 | Appraisal results | Present quality assessment results and indicate which articles were weighted or excluded based on the assessment. | yes | Subchapter Study quality & Suppl. Table S8 |
| 14 | Data extraction | Indicate which sections of primary studies were analyzed and how data were extracted (e.g., results entered into software). | yes | Subchapter Data extraction |
| 15 | Software | State the software used, if any. | Yes | Covidence |
| 16 | Number of reviewers | Identify who was involved in coding and analysis. | yes | Subchapter Synthesis of qualitative studies |
| 17 | Coding | Describe the process for coding data (e.g., line-by-line coding to search for concepts). | yes | Subchapter Synthesis of qualitative studies |
| 18 | Study comparison | Describe how comparisons were made within and across studies (e.g., coding into pre-existing concepts or creating new ones). | yes | Subchapters Synthesis of qualitative studies in the methods and results section |
| 19 | Derivation of themes | Explain whether the derivation of themes or constructs was inductive or deductive. | yes | Subchapters Synthesis of qualitative studies in the methods and results section |
| 20 | Quotations | Provide quotations from primary studies to illustrate themes or constructs and identify their origin (participant or author's interpretation). | no | *not conducted due to a mixed-methods review type and limited space* |
| 21 | Synthesis output | Present rich, compelling, and useful results that go beyond summarizing the primary studies (e.g., new interpretations, conceptual models, or theories). | yes | Subchapters Synthesis of qualitative studies & Mixed-methods meta-synthesis |

*From:* Tong, A., Flemming, K., McInnes, E., Oliver, S., & Craig, J. (2012). Enhancing transparency in reporting the synthesis of qualitative research: ENTREQ. *BMC Medical Research Methodology*, *12*, 1-8.

# Supplementary Section S3: Deviation of the protocol

Regarding the study protocol (PROSPERO York database, CRD42024550490), we identified during the review process three necessary deviations from the original protocol:

1) Title Modification: The original PROSPERO protocol included "transport-related physical activity" in the title. However, as no studies addressing this topic were identified, we excluded this term from the final title to reflect the actual scope of the review.

2) Risk of Bias Assessment: The original protocol specified the use of four different risk of bias tools (RoB 2.0, ROBINS-I, NIH Quality Assessment Tool for Observational Cohort and Cross-Sectional Studies, and the CASP Checklist for Qualitative Studies). Due to the heterogeneity in study designs among the included studies, the review team determined that using a single, more adaptable tool (MMAT) was more appropriate for consistent assessment of risk of bias across all studies.

3) For the synthesis, we were able to conduct a meta-analysis, which was not initially planned. Thus, the term “meta-analysis" was also included in the title.

# Supplementary Section S4: Decision tree for including and excluding relevant studies

DECISION TREE FOR SCREENING

| 1. Study design - basic  - Is this an original study?   *The results presented are original and not a secondary analysis of another study’s data.* | **No →** | Exclude  Reason: No original data |
| --- | --- | --- |
| **Yes ↓** |  |  |
| 1. Study design - advanced  - Does the study design fit to our scope?   Qua**l**itative studies to include:  - Interviews (focus group, single) and observations  *Search terms: Document Analysis or focus groups or interviews as topic or narration or qualitative research or "personal narratives as topic" or Surveys and Questionnaires or depth or face or group or guided or indepth or informal or semistructured or structured or unstructured or discussions or interview or questionnaire or ethnographic or ethnography or fieldwork or informants or studies or synthesis or mixed method*  *🡪 “cross-sectional” qualitative studies which ask at one measurement point about the relevant variables resp. content, but in a retrospective manner, can be included*  Quantitative studies to include:  *Search terms: cohort studies OR longitudinal studies OR follow-up studies OR prospective studies OR retrospective studies OR Non-Randomized Controlled Trials as Topic OR Observational Study OR quasi-experimental OR experimental OR before-and-after OR pre-post OR intervention OR follow-up OR exp randomized controlled trial OR controlled clinical trial OR placebo OR clinical trials as topic*  Mixed-methods studies to include: if the criteria for the qualitative and/or quantitative studies are met  Studies to exclude:  - Cross-sectional quantitative studies (measuring IV & DV at the same time)  - any types of reviews and not original empirical, peer-reviewed research (e.g., comments, editorials, congress contributions etc.)  - single-bout interventions (e.g., cross-over trial with each exercise condition 1x 20min) | **No →** | Exclude  Reason: Wrong study design |
| **Yes ↓** |  |  |
| 1. Sample  - Does the study sample fit to our scope?   Age:  Individuals between 10-19 years of age (WHO-Definition of adolescence)  🡪 decisive is the mean age, which has to be within this range (mean ages 9.9y and 19.1y will be excluded)  *Search terms: Adolescent or Young Adult or teen or youth or juvenile or early adulthood or young person/individual/people/population or girl or boy or student or secondary schooler or middle schooler or high schooler or highschooler*  Type of population:  - general population: no specific focus at the recruitment  - at-risk population: e.g., low SES, special living conditions (e.g., orphanage), mental health issues but no diagnosed illness/disorder  Excluding criteria:  - a targeted sample with a diagnosed(!) illness/disorder (e.g., depression, obesity, ADHD, Asthma etc.), i.e. the sample is recruited specifically to research people with a certain illness/disorder  *Search terms: anorexia OR arrhythmia OR cancer OR cardiovascular disease OR chemotherap* OR chronic OR concussion OR cystic fibrosis OR diabet* OR disorderer OR disabled OR disease* OR doping OR epilepsy OR illness* OR implant* OR injur* OR myocardial infarct* OR multiple sclerosis OR orthodont* OR orthopedic OR orthopaedic OR pain OR patient* OR postpartum OR pregnan* OR psychiatr* OR rehabilitat* OR screening OR sick OR sickness OR surg* OR syndrome* OR suicid* OR therap* OR transplant**  - studies which focus specific on covid (e.g., only at-home exercising due to lockdown, pre-post-lockdown differences) | **No →** | Exclude  Wrong Age or diagnose as not healthy |
| **Yes ↓** |  |  |
| 1. Dependent variable: (positive) mental health  - Does the study measure at least one positive-mental-health-related outcome?   That should include at least one aspect of psychological, emotional, physical or social well-being or aspects of the self-concept, body image etc.  Search terms: Self-Concept OR Mental Health OR Psychological Well-Being OR Body image OR Connectedness OR Emotional health OR Happiness OR Life quality OR Life satisfaction OR Mental health OR Optimism OR Perceived -appearance, - competence OR fitness OR mood or health status OR (Positive -affect, -mood* OR Psychological health OR Psychosocial health OR Quality of life OR Relaxation OR Satisfaction with life OR Self-confidence OR Self-concept OR Self-efficacy or Self-esteem OR Self-image OR Self-perception OR Sense of belonging OR Social health OR Vitality OR Well-being OR Wellbeing  Studies with the following instruments can be included too:  POMS, PANAS  Excluding criteria:  - Aspects of ill-being including the absence of ill-being (depressive symptoms, anxiety, stress etc.)  - aspects of mental health which are closely related to PA, e.g., self-efficacy for PA; motivation for PA  Studies with the following instruments can be excluded too:  SCL-90, GHQ-12, GHQ-28 | **No →** | Exclude  Reason: Wrong measures |
| **Yes ↓** |  |  |
| 1. Independent Variable: specific type of PA  - Does the study measure a specific type of PA in leisure?   Including criteria:  Specific type of PA during leisure-time  *Search terms: walking or running or jogging or swimming or exercise or exercising or strength training* or resistance training* or weight training* or aerobics or physical endurance* or physical fitness or physical strength* or physical conditioning* or physical training* or biking or bike? or bicycl* or recreation* - activit*, -sport or athletics or badminton or ballet or baseball or basketball or biathlon or bodybuilding or boxing or canoeing or cross-fit or dancing or dance or fencing or football or golf or golfing or gymnast* or gym or hiking or hockey or horse-riding or horseback-riding or judo or jiu-jitsu or ju-jitsu or karate or kayaking or kung-fu or marathon or martial-art* or orienteering or padel or pilates or rowing or rugby or skating or skateboarding or skiing or soccer or softball or surfing or squash or taekwondo or Tai Chi or Tai Ji or tennis or triathlon or volleyball or water-polo or waterpolo or wrestling or yoga or zumba* 🡪 or further specific LTPA including exergaming etc.  Excluding criteria:  - No specific type of PA (e.g., general PA, frequency, intensity, only the setting etc.) and other aspects of PA.  *Excluding terms:* *Athletic Performance or Return to Sport or Sports for Persons with Disabilities or Cool-Down Exercise or Muscle Stretching Exercises or Physical Conditioning, Animal or Post-Exercise Recovery or Preoperative Exercise or Stair Climbing or Warm-Up Exercise or biofeedback or muscle relaxation*  - PA within compulsory/mandatory school (physical education classes, active breaks in classroom etc.)  🡪 Aim: *leisure*-time physical activity  🡪 if there is a mixed IV or intervention (e.g., PA & educational aspects), we include studies if there is an included educational part within the PA-session (e.g., at the beginning or end of the PA-session with some inputs or reflections), and we exclude studies if there are separate educational/nutritional sessions)  🡪 if an already physically active sample is in focus, we have to distinguish if they are intervened by another LTPA (e.g., yoga for football players) 🡪 include;  or if given aspects of their own activity (e.g., additional small sided games for football players) 🡪 exclude | **No →** | Exclude  Reason: wrong kind/aspect of or no specific type of physical activity |
| **Include!** |  |  |

# Supplementary Table S5: Overview of databases & search results

**Overview of databases & results**

Date of 1^st^ search**: May 30^th^, 2024.** Date last searched: **June 16^th^, 2025**

|  | Before deduplication | After deduplication |  | Before deduplication | After deduplication |
| --- | --- | --- | --- | --- | --- |
| Medline ALL (Ovid) | 1873 |  |  | 2332 |  |
| Embase.com | 1819 |  |  | 2415 |  |
| PsycInfo (Ovid) | 1015 |  |  | 1244 |  |
| Cochrane CENTRAL | 2037 |  |  | 2198 |  |
| SPORTDiscus (via EBSCOhost) | 1208 |  |  | 1297 |  |
| CINAHL with Full Text (via EBSCOhost) | 1925 |  |  | 1630 |  |
| Web of Science | 2337 |  |  | 2694 |  |
| Total | **12114** | **7129** |  | **13810** | **8149** |

Totally **4985 duplicates removed** using Deduklick^1^ Totally **5661 duplicates removed** using Deduklick^1^

**1558 new hits found by the update search^2^**

^1^Duplicate records are removed using a fully automated AI-based deduplication solution, Deduklick [1].

^2^The discrepancy between the new hits found (1558) and the difference between the initial search and updated search after deduplication (initial search 7129, updated search 8149, difference 1020) is common by using the Bramer-Method [2]. In the PRISMA flowchart, the total hits of the updated search 8149 are reported, as suggested [2].

[1] Borissov, N.; Haas, Q.; Minder, B.; Kopp-Heim, D.; von Gernler, M.; Janka, H.; Teodoro, D.; Amini, P. Reducing systematic review burden using Deduklick: A novel, automated, reliable, and explainable deduplication algorithm to foster medical research. *Syst. Rev.* 2022, 11, 172.

[2] Bramer WM, Giustini D, De Jonge GB, Holland L, Bekhuis T. De-duplication of database search results for systematic reviews in EndNote. *J Med Libr Assoc*. 2016 Jul;104(3):240–3.

**Detailed search strategies for supplementary material**

Ovid MEDLINE(R) ALL <1946 to June 16, 2025>
<https://ovidsp.ovid.com/ovidweb.cgi?T=JS&NEWS=N&PAGE=main&SHAREDSEARCHID=24iRsgQHLJPtTknNOTQFpz1ug0j2nwGlCkoZIPInyN52qCNJiwu5z8KbfCSyfXWFd>

| **#** | **Query** | **Results from 16 Jun 2025** |
| --- | --- | --- |
| 1 | (exp *Exercise/ not (Cool-Down Exercise/ or Muscle Stretching Exercises/ or Physical Conditioning, Animal/ or Post-Exercise Recovery/ or Preoperative Exercise/ or Stair Climbing/ or Warm-Up Exercise/)) or (exp *Sports/ not (exp Athletic Performance/ or Return to Sport/ or Sports for Persons with Disabilities/)) or (physical activit* or sport? or walking or running or jogging or swimming or exercise? or exercising or strength training* or resistance training* or weight training* or aerobics or physical endurance* or physical fitness or physical strength* or physical conditioning* or physical training* or active transport* or commut* or travel mode* or biking or bike? or bicycl* or (recreation* adj2 (activit* or sport?)) or athletics or badminton or ballet or baseball or basketball or biathlon or bodybuilding or boxing or canoeing or cross-fit or dancing or dance? or fencing or football or golf? or golfing or gymnast* or gym or hiking or hockey or horse-riding or horseback-riding or judo or jiu-jitsu or ju-jitsu or karate or kayaking or kung-fu or marathon or martial-art* or orienteering or padel or pilates or rowing or rugby or skating or skateboarding or skiing or soccer or softball or surfing or squash or taekwondo or Tai Chi or Tai Ji or tennis or triathlon or volleyball or water-polo or waterpolo or wrestling or yoga or zumba).ti,kf. | 485,721 |
| 2 | exp *Self Concept/ or *Mental Health/ or *Psychological Well-Being/ or (Body image or Connectedness or Emotional health or Happiness or Health literacy or Life quality or Life satisfaction or Mental health or Optimism or (Perceived adj3 (appearance* or competence* or fitness or mood* or "health status")) or (Positive adj3 (affect* or mood*)) or Psychologic* health or Psychosocial health or Quality of life or Relaxation or Resilience or Satisfaction with life or Self-confidence or Self-concept or Self-efficacy or Self-esteem* or Self-image or Self-perception* or Sense of belonging or Social health or Vitality or Well-being or Wellbeing).ti,kf. | 470,615 |
| 3 | Adolescent/ or Young Adult/ or (adolescen* or teen* or youth* or juvenil* or young adult* or early adulthood or young person* or young individual* or young people* or young population* or girl or girls or boy or boys or student? or secondary schooler? or middle schooler? or high schooler? or highschooler? or secondary school? or middle school? or high school? or highschool?).ab,ti. | 3,596,033 |
| 4 | (("Document Analysis"/ or focus groups/ or interviews as topic/ or narration/ or qualitative research/ or exp "personal narratives as topic"/ or "Surveys and Questionnaires"/ or ((depth or face or group or guided or indepth or informal or semistructured or structured or unstructured) adj4 (discussion or discussions or interview or interviewed or interviews or questionnaire or questionnaires)).ti,ab. or (ethnographic or ethnography or mixed method* or (field adj1 work) or fieldwork or (focus adj1 (group or groups)) or (groups adj2 interviewed) or (key adj1 (informant or informants)) or (qualitative adj2 (research or studies or studies or synthesis))).ti,ab. or (cohort studies/ or longitudinal studies/ or follow-up studies/ or prospective studies/ or retrospective studies/ or Non-Randomized Controlled Trials as Topic/ or Observational Study/ or (cohort* or longitudinal or prospective or observation* or retrospective or quasi-experimental or experimental or before-and-after or pre-post or intervention* or follow-up or followup).ab,ti.) or (exp randomized controlled trial/ or controlled clinical trial.pt. or random*.ab. or placebo.ab. or clinical trials as topic/ or trial.ti.)) not (letter or news or comment or editorial or congress).pt. not (exp animals/ not humans/) not ((Review or Systematic Review or meta analysis).pt. or Cross-Sectional Studies/ or systematic review.ti. or literature review.ti. or umbrella review.ti. or review of reviews.ti. or cross-sectional.ti.) not (anorexia or arrhythmia* or cancer* or cardiovascular disease* or chemotherap* or chronic or concussion or cystic fibrosis or diabet* or disorder* or disable* or disabilit* or disease* or doping or epilepsy or illness* or implant* or injur* or myocardial infarct* or multiple sclerosis or orthodont* or orthopedic or orthopaedic or pain or patient* or postpartum or pregnan* or psychiatr* or rehabilitat* or screening or sick or sickness or surg* or syndrome* or suicid* or therap* or transplant*).ti.) and 2009:2026.(sa_year). | 2,910,781 |
| 5 | 1 and 2 and 3 and 4 | 2,332 |

**Embase.com (Elsevier)**

| **#** | **Query** | **Results from 16 Jun 2025** |
| --- | --- | --- |
| 1 | ('exercise'/exp/mj not ('cool down'/de or 'stretching exercise'/de or 'exercise recovery'/de or 'preoperative exercise'/de or 'stair climbing'/de or 'warm up'/de)) or 'physical activity'/mj or ('sport'/exp/mj not ('athletic performance'/de or athlete/exp/mj or 'cardiorespiratory fitness'/de or 'endurance'/de or 'return to sport'/de or 'disabled sport'/exp)) or ('physical activit*' or sport* or walking or running or jogging or swimming or exercise* or exercising or 'strength training*' or 'resistance training*' or 'weight training*' or aerobics or 'physical endurance*' or 'physical fitness' or 'physical strength*' or 'physical conditioning*' or 'physical training*' or 'active transport*' or commut* or 'travel mode*' or biking or bike or bicycl* or (recreation* NEAR/2 (activit* or sport*)) or athletics or badminton or ballet or baseball or basketball or biathlon or bodybuilding or boxing or canoeing or cross-fit or dancing or dance or fencing or football or golf or golfing or gymnast* or gym or hiking or hockey or horse-riding or horseback-riding or judo or jiu-jitsu or ju-jitsu or karate or kayaking or kung-fu or marathon or martial-art* or orienteering or padel or pilates or rowing or rugby or skating or skateboarding or skiing or soccer or softball or surfing or squash or taekwondo or Tai-Chi or Tai-Ji or tennis or triathlon or volleyball or water-polo or waterpolo or wrestling or yoga or zumba):ti,kw | 621,575 |
| 2 | 'self concept'/exp/mj or 'mental health'/exp/mj or 'psychological well-being'/mj or ('Body image' or Connectedness or 'Emotional health' or Happiness or 'Health literacy' or 'Life quality' or 'Life satisfaction' or 'Mental health' or Optimism or (Perceived NEAR/3 (appearance* or competence* or fitness or mood* or 'health status')) or (Positive NEAR/3 (affect* or mood*)) or 'Psychologic* health' or 'Psychosocial health' or 'Quality of life' or Relaxation or Resilience or 'Satisfaction with life' or Self-confidence or Self-concept or Self-efficacy or Self-esteem* or Self-image or Self-perception* or 'Sense of belonging' or 'Social health' or Vitality or Well-being or Wellbeing):ti,kw | 620,153 |
| 3 | 'adolescent'/de or 'adolescence'/exp or 'young adult'/de or 'juvenile'/de or (adolescen* or teen* or youth* or juvenil* or 'young adult*' or 'early adulthood' or 'young person*' or 'young individual*' or 'young people*' or 'young population*' or girl or girls or boy or boys or student or students or 'secondary schooler*' or 'middle schooler*' or 'high schooler*' or highschooler* or ((secondary or middle or high) NEXT/2 (school or schools)) or highschool or highschools):ab,ti | 3,594,440 |
| 4 | 'qualitative research'/exp OR 'interview'/exp OR 'narrative'/exp OR 'storytelling'/exp OR 'grounded theory'/exp OR 'observational study'/exp OR 'recording'/exp OR 'thematic analysis'/exp OR 'content analysis'/exp OR 'ethnographic research'/exp OR 'field study'/exp OR 'participant observation'/exp OR 'phenomenology'/exp OR 'qualitative analysis'/exp OR 'qualitative methods'/exp OR 'in depth interview'/exp OR 'face to face interview'/exp OR 'thematic analys*':ab,ti,kw OR 'content analys*':ab,ti,kw OR 'focus group*':ab,ti,kw OR ethnograph*:ab,ti,kw OR ethnograf*:ab,ti,kw OR etnograf*:ab,ti,kw OR 'field stud*':ab,ti,kw OR phenomenolog*:ab,ti,kw OR narration*:ab,ti,kw OR narrative:ab,ti,kw OR 'qualitative stud*':ab,ti,kw OR 'qualitative analys*':ab,ti,kw OR 'qualitative research*':ab,ti,kw OR 'qualitative method*':ab,ti,kw OR multimethodolog*:ab,ti,kw OR 'mixed method*':ab,ti,kw OR observation*:ab,ti,kw OR 'grounded theory':ab,ti,kw OR 'audio recording*':ab,ti,kw OR 'tape recording*':ab,ti,kw OR audiotape*:ab,ti,kw OR (('semi-structured':ab,ti,kw OR semistructured:ab,ti,kw OR unstructured:ab,ti,kw OR informal:ab,ti,kw OR 'in-depth':ab,ti,kw OR indepth:ab,ti,kw OR 'face-to-face':ab,ti,kw OR structured:ab,ti,kw OR guide*:ab,ti,kw) AND (interview*:ab,ti,kw OR discussion*:ab,ti,kw OR questionnaire*:ab,ti,kw)) OR ('clinical trial'/exp OR randomization/exp OR 'prospective study'/exp OR 'longitudinal study'/exp OR 'retrospective study'/de OR 'cohort analysis'/de OR 'follow up'/de OR 'major clinical study'/de OR (trial* OR random* OR rct OR prospective* OR retrospective* OR longitudinal* OR cohort* OR observation* OR quasi-experimental OR experimental OR before-and-after OR pre-post OR intervention* OR follow-up* OR followup):ab,ti) NOT ([Conference Abstract]/lim OR [Letter]/lim OR [Note]/lim OR [Editorial]/lim) NOT ([animals]/lim NOT [humans]/lim) NOT ('systematic review'/de OR 'meta analysis'/exp OR 'cross-sectional study'/de OR 'systematic review':ti OR 'literature review':ti OR 'umbrella review':ti OR 'review of reviews':ti OR meta-analys*:ti OR cross-sectional:ti) NOT (anorexia or arrhythmia* or cancer* or 'cardiovascular disease*' or chemotherap* or chronic or concussion or 'cystic fibrosis' or diabet* or disorder* or disable* or disabilit* or disease* or doping or epilepsy or illness* or implant* or injur* or 'myocardial infarct*' or 'multiple sclerosis' or orthodont* or orthopedic or orthopaedic or pain or patient* or postpartum or pregnan* or psychiatr* or rehabilitat* or screening or sick or sickness or surg* or syndrome* or suicid* or therap* or transplant*):ti AND [2009-2026]/py | 3,857,751 |
| 5 | 1 and 2 and 3 and 4 | 2,415 |

**APA PsycInfo** <1806 to June 2025 Week 2>
(via OvidSP)

<https://ovidsp.ovid.com/ovidweb.cgi?T=JS&NEWS=N&PAGE=main&SHAREDSEARCHID=5q4o07kH2KUbylynI6KbnmMCNjsuKibLYrADeRAAkBMXMUV8XZuzihQi7w5frx5Jy>

| **#** | **Query** | **Results from  16 Jun 2025** |
| --- | --- | --- |
| 1 | exp *Exercise/ or exp *physical activity/ or (exp *Sports/ not exp Athletic Performance/) or (physical activit* or sport? or walking or running or jogging or swimming or exercise? or exercising or strength training* or resistance training* or weight training* or aerobics or physical endurance* or physical fitness or physical strength* or physical conditioning* or physical training* or active transport* or commut* or travel mode* or biking or bike? or bicycl* or (recreation* adj2 (activit* or sport?)) or athletics or badminton or ballet or baseball or basketball or biathlon or bodybuilding or boxing or canoeing or cross-fit or dancing or dance? or fencing or football or golf? or golfing or gymnast* or gym or hiking or hockey or horse-riding or horseback-riding or judo or jiu-jitsu or ju-jitsu or karate or kayaking or kung-fu or marathon or martial-art* or orienteering or padel or pilates or rowing or rugby or skating or skateboarding or skiing or soccer or softball or surfing or squash or taekwondo or Tai Chi or Tai Ji or tennis or triathlon or volleyball or water-polo or waterpolo or wrestling or yoga or zumba).ti,id. | 129,704 |
| 2 | exp *Self-Concept/ or exp *mental health/ or exp *well being/ or (Body image or Connectedness or Emotional health or Happiness or Health literacy or Life quality or Life satisfaction or Mental health or Optimism or (Perceived adj3 (appearance* or competence* or fitness or mood* or "health status")) or (Positive adj3 (affect* or mood*)) or Psychologic* health or Psychosocial health or Quality of life or Relaxation or Resilience or Satisfaction with life or Self-confidence or Self-concept or Self-efficacy or Self-esteem* or Self-image or Self-perception* or Sense of belonging or Social health or Vitality or Well-being or Wellbeing).ti,id. | 453,094 |
| 3 | (adolescen* or teen* or youth* or juvenil* or young adult* or early adulthood or young person* or young individual* or young people* or young population* or girl or girls or boy or boys or student? or secondary schooler? or middle schooler? or high schooler? or highschooler? or secondary school? or middle school? or high school? or highschool?).ab,ti. | 1,109,839 |
| 4 | (((("semi-structured" or semistructured or unstructured or informal or "in-depth" or indepth or "face-to-face" or structured or guide or guides) adj3 (interview* or discussion* or questionnaire*)) or (focus group* or qualitative or ethnograph* or mixed method* or fieldwork or "field work" or "key informant")).ti,ab,id. or exp qualitative research/ or exp interviews/ or exp group discussion/ or qualitative study.md. or (cohort or longitudinal or prospective or retrospective or observation* or quasi-experimental or experimental or before-and-after or pre-post or intervention* or follow-up or followup).ti,ab,id. or longitudinal study.md. or prospective study.md. or retrospective study.md. or exp clinical trials/ or randomized clinical trials/ or exp randomized controlled trials/ or "treatment outcome clinical trial".md. or ((randomi?ed adj7 trial*) or ((single or doubl* or tripl* or treb*) and (blind* or mask*)) or (controlled adj3 trial*) or (clinical adj2 trial*)).ti,ab,id.) not ("Literature Review" or meta-analysis).md. not (anorexia or arrhythmia* or cancer* or cardiovascular disease* or chemotherap* or chronic or concussion or cystic fibrosis or diabet* or disorder* or disable* or disabilit* or disease* or doping or epilepsy or illness* or implant* or injur* or myocardial infarct* or multiple sclerosis or orthodont* or orthopedic or orthopaedic or pain or patient* or postpartum or pregnan* or psychiatr* or rehabilitat* or screening or sick or sickness or surg* or syndrome* or suicid* or therap* or transplant*).ti. not cross-section*.ti. not case-report*.ti. | 1,296,586 |
| 5 | 1 and 2 and 3 and 4 | 1,918 |
| 6 | limit 5 to yr="2009 -Current" | 1,590 |
| 7 | limit 6 to peer reviewed journal | 1,244 |

**Cochrane CENTRAL of Controlled Trials**Issue 6 of 12, June 2025

ID Search Hits

#1 ((physical NEXT activit*) or sport* or walking or running or jogging or swimming or exercise* or exercising or (strength NEXT training*) or (resistance NEXT training*) or (weight NEXT training*) or aerobics or (physical NEXT endurance*) or (physical NEXT fitness) or (physical NEXT strength*) or (physical NEXT conditioning*) or (physical NEXT training*) or (active NEXT transport*) or commut* or (travel NEXT mode*) or biking or bike or bicycl* or (recreation* NEAR/2 (activit* or sport*)) or athletics or badminton or ballet or baseball or basketball or biathlon or bodybuilding or boxing or canoeing or cross-fit or dancing or dance or fencing or football or golf or golfing or gymnast* or gym or hiking or hockey or horse-riding or horseback-riding or judo or jiu-jitsu or ju-jitsu or karate or kayaking or kung-fu or marathon or (martial NEXT art*) or orienteering or padel or pilates or rowing or rugby or skating or skateboarding or skiing or soccer or softball or surfing or squash or taekwondo or Tai-Chi or Tai-Ji or tennis or triathlon or volleyball or water-polo or waterpolo or wrestling or yoga or zumba):ti,kw 157389

#2 ("Body image" or Connectedness or "Emotional health" or Happiness or "Health literacy" or "Life quality" or "Life satisfaction" or "Mental health" or Optimism or (Perceived NEAR/3 (appearance* or competence* or fitness or mood* or health-status)) or (Positive NEAR/3 (affect* or mood*)) or (Psychologic* NEXT health) or "Psychosocial health" or "Quality of life" or Relaxation or Resilience or "Satisfaction with life" or Self-confidence or Self-concept or Self-efficacy or Self-esteem* or Self-image or Self-perception* or "Sense of belonging" or "Social health" or Vitality or Well-being or Wellbeing):ti,kw 146977

#3 (adolescen* or teen* or youth* or juvenil* or (young NEXT adult*) or "early adulthood" or (young NEXT person*) or (young NEXT individual*) or (young NEXT people*) or (young NEXT population*) or girl or girls or boy or boys or student or students or (secondary NEXT schooler*) or (middle NEXT schooler*) or (high NEXT schooler*) or highschooler* or ((secondary or middle or high) NEXT/2 (school or schools)) or highschool or highschools):ab,ti,kw 300567

#4 #1 AND #2 AND #3 4186

#5 (anorexia or arrhythmia* or cancer* or (cardiovascular NEXT disease*) or chemotherap* or chronic or concussion or "cystic fibrosis" or diabet* or disorder* or disable* or disabilit* or disease* or doping or epilepsy or illness* or implant* or injur* or (myocardial NEXT infarct*) or "multiple sclerosis" or orthodont* or orthopedic or orthopaedic or pain or patient* or postpartum or pregnan* or psychiatr* or rehabilitat* or screening or sick or sickness or surg* or syndrome* or suicid* or therap* or transplant*):ti 1082010

#6 #4 NOT #5 2425

#7 Year range: 2009 to 2025 2198 Trials

**SPORTDiscus with Full Text (via EBSCOhost)**

| # | **Query** Expanders - Apply equivalent subjects Search modes - Find all my search terms |
| --- | --- |

| S11 | S9 NOT S10 | **1,297** |
| --- | --- | --- |
| S10 | TI ((athlete* NOT non-athlet*)) | 19,439 |
| S9 | S5 NOT S6 **Limiters** - **Publication Date**: **20090101-20251231**; Peer Reviewed |  |
| S8 | S5 NOT S6 **Limiters** - **Peer Reviewed** |  |
| S7 | S5 NOT S6 | 1,834 |
| S6 | TI ( ("systematic review" or "literature review" or "umbrella review" or "review of reviews" or cross-sectional) ) OR TI ( (anorexia or arrhythmia* or cancer* or "cardiovascular disease*" or chemotherap* or chronic or concussion or cystic fibrosis or diabet* or disorder* or disable* or disabilit* or disease* or doping or epilepsy or illness* or implant* or injur* or "myocardial infarct*" or "multiple sclerosis" or orthodont* or orthopedic or orthopaedic or pain or patient* or postpartum or pregnan* or psychiatr* or rehabilitat* or screening or sick or sickness or surg* or syndrome* or suicid* or therap* or transplant*) ) | 262,027 |
| S5 | S1 AND S2 AND S3 AND S4 | 2,076 |
| S4 | TI ( (((depth or face or group or guided or indepth or informal or semistructured or structured or unstructured) N4 (discussion or discussions or interview or interviewed or interviews)) or questionnaire or questionnaires or survey or surveys or (ethnographic or ethnography or (field N1 work) or fieldwork or (focus N1 (group or groups)) or (groups N2 interviewed) or (key N1 (informant or informants)) or (qualitative N2 (research or studies or studies or synthesis)) or (cohort* or longitudinal or prospective or observation* or retrospective or quasi-experimental or experimental or before-and-after or pre-post or intervention* or follow-up or followup or random* or RCT or placebo or trial*)) ) OR AB ( (((depth or face or group or guided or indepth or informal or semistructured or structured or unstructured) N4 (discussion or discussions or interview or interviewed or interviews)) or questionnaire or questionnaires or survey or surveys or mixed method* or (ethnographic or ethnography or (field N1 work) or fieldwork or (focus N1 (group or groups)) or (groups N2 interviewed) or (key N1 (informant or informants)) or (qualitative N2 (research or studies or studies or synthesis)) or (cohort* or longitudinal or prospective or observation* or retrospective or quasi-experimental or experimental or before-and-after or pre-post or intervention* or follow-up or followup or random* or RCT or placebo or trial*)) ) OR KW ( (((depth or face or group or guided or indepth or informal or semistructured or structured or unstructured) N4 (discussion or discussions or interview or interviewed or interviews)) or questionnaire or questionnaires or survey or surveys or mixed method* or (ethnographic or ethnography or (field N1 work) or fieldwork or (focus N1 (group or groups)) or (groups N2 interviewed) or (key N1 (informant or informants)) or (qualitative N2 (research or studies or studies or synthesis)) or (cohort* or longitudinal or prospective or observation* or retrospective or quasi-experimental or experimental or before-and-after or pre-post or intervention* or follow-up or followup or random* or RCT or placebo or trial*)) ) | 392,767 |
| S3 | TI ( (adolescen* or teen* or youth* or juvenil* or "young adult*" or "early adulthood" or "young person*" or "young individual*" or "young people*" or "young population*" or girl or girls or boy or boys or student* or "secondary schooler*" or "middle schooler*" or "high schooler*" or highschooler* or "secondary school" or "middle school" or "high school" or highschool) ) OR AB ( (adolescen* or teen* or youth* or juvenil* or "young adult*" or "early adulthood" or "young person*" or "young individual*" or "young people*" or "young population*" or girl or girls or boy or boys or student* or "secondary schooler*" or "middle schooler*" or "high schooler*" or highschooler* or "secondary school" or "middle school" or "high school" or highschool) ) OR KW ( (adolescen* or teen* or youth* or juvenil* or "young adult*" or "early adulthood" or "young person*" or "young individual*" or "young people*" or "young population*" or girl or girls or boy or boys or student* or "secondary schooler*" or "middle schooler*" or "high schooler*" or highschooler* or "secondary school" or "middle school" or "high school" or highschool) ) | 209,752 |
| S2 | TI ( ((Body W1 image) or Connectedness or (Emotional W1 health) or Happiness or (Health W1 literacy) or (Life W1 quality) or (Life W1 satisfaction) or "Mental health" or Optimism or (Perceived N2 (appearance* or competence* or fitness or mood* or "health status")) or (Positive N2 (affect* or mood*)) or (Psychologic* W1 health) or "Psychosocial health" or "Quality of life" or Relaxation or Resilience or "Satisfaction with life" or Self-confidence or Self-concept or Self-efficacy or Self-esteem* or Self-image or Self-perception* or "Sense of belonging" or "Social health" or Vitality or Well-being or Wellbeing) ) OR KW ( ((Body W1 image) or Connectedness or (Emotional W1 health) or Happiness or (Health W1 literacy) or (Life W1 quality) or (Life W1 satisfaction) or "Mental health" or Optimism or (Perceived N2 (appearance* or competence* or fitness or mood* or "health status")) or (Positive N2 (affect* or mood*)) or (Psychologic* W1 health) or "Psychosocial health" or "Quality of life" or Relaxation or Resilience or "Satisfaction with life" or Self-confidence or Self-concept or Self-efficacy or Self-esteem* or Self-image or Self-perception* or "Sense of belonging" or "Social health" or Vitality or Well-being or Wellbeing) ) | 36,515 |
| S1 | TI ( ((physical W1 activit*) or sport or sports or walking or running or jogging or swimming or exercise or exercises or exercising or (strength W1 training*) or (resistance W1 training*) or (weight W1 training*) or aerobics or (physical W1 endurance*) or (physical W1 fitness) or (physical W1 strength*) or (physical W1 conditioning*) or (physical W1 training*) or (active W1 transport*) or commut* or (travel* W1 mode*) or biking or bike or bikes or bicycl* or (recreation* N2 (activit* or sport*)) or athletics or badminton or ballet or baseball or basketball or biathlon or bodybuilding or boxing or canoeing or cross-fit or dancing or dance or fencing or football or golf or golfing or gymnast* or gym or hiking or hockey or (horse W1riding) or (horseback W1 riding) or judo or jiu-jitsu or ju-jitsu or karate or kayaking or kung-fu or marathon or (martial W1 art*) or orienteering or padel or pilates or rowing or rugby or skating or skateboarding or skiing or soccer or softball or surfing or squash or taekwondo or Tai-Chi or Tai-Ji or tennis or triathlon or volleyball or water-polo or waterpolo or wrestling or yoga or zumba) ) OR KW ( ((physical W1 activit*) or sport or sports or walking or running or jogging or swimming or exercise or exercises or exercising or (strength W1 training*) or (resistance W1 training*) or (weight W1 training*) or aerobics or (physical W1 endurance*) or (physical W1 fitness) or (physical W1 strength*) or (physical W1 conditioning*) or (physical W1 training*) or (active W1 transport*) or commut* or (travel* W1 mode*) or biking or bike or bikes or bicycl* or (recreation* N2 (activit* or sport*)) or athletics or badminton or ballet or baseball or basketball or biathlon or bodybuilding or boxing or canoeing or cross-fit or dancing or dance or fencing or football or golf or golfing or gymnast* or gym or hiking or hockey or (horse W1riding) or (horseback W1 riding) or judo or jiu-jitsu or ju-jitsu or karate or kayaking or kung-fu or marathon or (martial W1 art*) or orienteering or padel or pilates or rowing or rugby or skating or skateboarding or skiing or soccer or softball or surfing or squash or taekwondo or Tai-Chi or Tai-Ji or tennis or triathlon or volleyball or water-polo or waterpolo or wrestling or yoga or zumba) ) | 851,698 |

**CINAHL (via EBSCOhost)**Cumulative Index to Nursing and Allied Health Literature CINAHL with Full Text

| # | **Query** Expanders - Apply equivalent subjects Search modes - Find all my search terms |
| --- | --- |

| S12 | S9 NOT S10 **Limiters** - **Publication Date: 20090101-20251231** | **1,630** |
| --- | --- | --- |
| S11 | S9 NOT S10 | 1,850 |
| S10 | TI ( ("systematic review" or "literature review" or "umbrella review" or "review of reviews" or cross-sectional) ) OR TI ( (anorexia or arrhythmia* or cancer* or "cardiovascular disease*" or chemotherap* or chronic or cerebrovascular or concussion or cystic fibrosis or diabet* or disorder* or disable* or disabilit* or disease* or doping or epilepsy or illness* or implant* or injur* or "myocardial infarct*" or "multiple sclerosis" or neuromuscular* or neoplas* or orthodont* or orthopedic or orthopaedic or pain or patient* or postpartum or pregnan* or psychiatr* or rehabilitat* or screening or sick or sickness or stroke or surg* or survivor* or syndrome* or suicid* or therap* or transplant*) ) | 2,991,405 |
| S9 | S4 AND S8 | 2,526 |
| S8 | S5 OR S6 OR S7 | 3,854,094 |
| S7 | ( MH "Prospective Studies+" OR MH "Case Control Studies" OR MH "Correlational Studies" ) OR TI ( (cohort* or longitudinal or prospective or observation* or retrospective or quasi-experimental or experimental or before-and-after or pre-post or intervention* or follow-up or followup) ) OR AB ( (cohort* or longitudinal or prospective or observation* or retrospective or quasi-experimental or experimental or before-and-after or pre-post or intervention* or follow-up or followup) ) | 1,893,798 |
| S6 | ((MH "Experimental Studies+") OR (MH "Multicenter Studies") OR (MH "Random Sample+") OR (MH "Placebos") OR (MH "Control (Research)+") OR (MH "Crossover Design") OR ((TI random* OR AB random*) OR (TI sham OR AB sham) OR (TI placebo* OR AB placebo*)) OR (((TI singl* OR AB singl*) OR (TI doubl* OR AB doubl*)) W1 ((TI blind* OR AB blind*) OR (TI dumm* OR AB dumm*) OR (TI mask* OR AB mask*))) OR (((TI tripl* OR AB tripl*) OR (TI trebl* OR AB trebl*)) W1 ((TI blind* OR AB blind*) OR (TI dumm* OR AB dumm*) OR (TI mask* OR AB mask*))) OR ((TI control* OR AB control*) N3 ((TI study OR AB study) OR (TI studies OR AB studies) OR (TI trial* OR AB trial*) OR (TI group* OR AB group*))) OR ((TI clinical OR AB clinical) N3 ((TI study OR AB study) OR (TI studies OR AB studies) OR (TI trial* OR AB trial*))) OR ((TI Nonrandom* OR AB Nonrandom*) OR (TI "non random*" OR AB "non random*") OR (TI "non-random*" OR AB "non-random*") OR (TI "quasi-random*" OR AB "quasi-random*") OR (TI quasirandom* OR AB quasirandom*)) OR ((TI phase OR AB phase) N6 ((TI study OR AB study) OR (TI studies OR AB studies) OR (TI trial* OR AB trial*))) OR (((TI crossover OR AB crossover) OR (TI "cross-over" OR AB "cross-over")) N3 ((TI study OR AB study) OR (TI studies OR AB studies) OR (TI trial* OR AB trial*))) OR (((TI multicent* OR AB multicent*) OR (TI "multi-cent*" OR AB "multi-cent*")) N3 ((TI study OR AB study) OR (TI studies OR AB studies) OR (TI trial* OR AB trial*))) OR (TI allocated OR AB allocated) OR (((TI "open label" OR AB "open label") OR (TI "open-label" OR AB "open-label")) N5 ((TI study OR AB study) OR (TI studies OR AB studies) OR (TI trial* OR AB trial*))) OR (((TI equivalence OR AB equivalence) OR (TI superiority OR AB superiority) OR (TI "non-inferiority" OR AB "non-inferiority") OR (TI noninferiority OR AB noninferiority)) N3 ((TI study OR AB study) OR (TI studies OR AB studies) OR (TI trial* OR AB trial*))) OR ((TI "pragmatic study" OR AB "pragmatic study") OR (TI "pragmatic studies" OR AB "pragmatic studies")) OR (((TI pragmatic OR AB pragmatic) OR (TI practical OR AB practical)) N3 (TI trial* OR AB trial*)) OR (((TI quasiexperimental OR AB quasiexperimental) OR (TI "quasi-experimental" OR AB "quasi-experimental")) N3 ((TI study OR AB study) OR (TI studies OR AB studies) OR (TI trial* OR AB trial*))) OR (TI trial)) | 1,312,949 |
| S5 | (MH “action research”) or (MH "Audiorecording") or (MH "cluster sample+") or (MH "constant comparative method") or (MH "content analysis") or (MH "discourse analysis") or (MH "ethnographic research") or (MH "ethnological research") or (MH "ethnography") or (MH "ethnonursing research") or (MH "field studies") or (MH "focus groups") or (MH "grounded theory") or (MH "Historical Records") or (MH "Interviews+") or (MH "Narratives") or (MH “naturalistic inquiry”) or (MH "observational methods+") or (MH "phenomenological research") or (MH "phenomenology") or (MH "purposive sample") or (MH "qualitative studies") or (MH "qualitative validity+") or (MH "questionnaires") or (MH "thematic analysis") or (MH "theoretical sample") or (MH "Videorecording+") or TX colaizzi* or TX constant comparative or TX constant comparison or TX cooperative inquir* or TX co-operative inquir* or TX co operative inquir* or TX Corbin* TX data saturat* or TX discourse* analysis or TX emic or TX etic or TX ethnon* or TX field research or TX field stud* or TX focus group* or TX Foucault* or TX giorgi* or TX Glaser* or TX grounded analysis or TX grounded research or TX grounded studies or TX grounded study or TX grounded theor* or TX heidegger* or TX hermeneutic* or TX heuristic or TX human science or TX husserl* or TX life experiences or TX life stor* or TX lived experience* or TX merleau ponty* or TX narrative analysis or TX qualitative or TX "mixed method*" or TX participant observ* or TX phenomenol* or TX purpos* sampl* or TX questionnaire* or TX semiotics or TX spiegelberg* or TX Strauss* TX van kaam* or TX van manen* | 2,407,857 |
| S4 | S1 AND S2 AND S3 | 2,889 |
| S3 | ( MH "Adolescence" or MH "Young Adult" ) OR TI ( (adolescen* or teen* or youth* or juvenil* or "young adult*" or "early adulthood" or "young person*" or "young individual*" or "young people*" or "young population*" or girl or girls or boy or boys or student* or "secondary schooler*" or "middle schooler*" or "high schooler*" or highschooler* or "secondary school" or "middle school" or "high school" or highschool) ) OR AB ( (adolescen* or teen* or youth* or juvenil* or "young adult*" or "early adulthood" or "young person*" or "young individual*" or "young people*" or "young population*" or girl or girls or boy or boys or student* or "secondary schooler*" or "middle schooler*" or "high schooler*" or highschooler* or "secondary school" or "middle school" or "high school" or highschool) ) | 1,074,977 |
| S2 | ( MM "Self Concept+" or MM "Mental Health" or MM "Psychological Well-Being" ) OR TI ( ((Body W1 image) or Connectedness or (Emotional W1 health) or Happiness or (Health W1 literacy) or (Life W1 quality) or (Life W1 satisfaction) or "Mental health" or Optimism or (Perceived N2 (appearance* or competence* or fitness or mood* or "health status")) or (Positive N2 (affect* or mood*)) or (Psychologic* W1 health) or "Psychosocial health" or "Quality of life" or Relaxation or Resilience or "Satisfaction with life" or Self-confidence or Self-concept or Self-efficacy or Self-esteem* or Self-image or Self-perception* or "Sense of belonging" or "Social health" or Vitality or Well-being or Wellbeing) ) | 236,031 |
| S1 | ( (MM "Exercise+" NOT (MH "Recovery, Exercise" OR MH "Stair Climbing" OR MH "Warm-Up Exercise+")) or (MM "Sports+" not (MH "Athletic Performance" or MH "Sports Re-Entry" or MH "Sports for Persons with Disabilities")) or MM "Physical Activity" ) OR TI ( ((physical W1 activit*) or sport or sports or walking or running or jogging or swimming or exercise or exercises or exercising or (strength W1 training*) or (resistance W1 training*) or (weight W1 training*) or aerobics or (physical W1 endurance*) or (physical W1 fitness) or (physical W1 strength*) or (physical W1 conditioning*) or (physical W1 training*) or (active W1 transport*) or commut* or (travel* W1 mode*) or biking or bike or bikes or bicycl* or (recreation* N2 (activit* or sport*)) or athletics or badminton or ballet or baseball or basketball or biathlon or bodybuilding or boxing or canoeing or cross-fit or dancing or dance or fencing or football or golf or golfing or gymnast* or gym or hiking or hockey or (horse W1riding) or (horseback W1 riding) or judo or jiu-jitsu or ju-jitsu or karate or kayaking or kung-fu or marathon or (martial W1 art*) or orienteering or padel or pilates or rowing or rugby or skating or skateboarding or skiing or soccer or softball or surfing or squash or taekwondo or Tai-Chi or Tai-Ji or tennis or triathlon or volleyball or water-polo or waterpolo or wrestling or yoga or zumba) ) | 251,550 |

**Web of Science** ***Core Collection** (via Clarivate)

*Science Citation Index Expanded (1900-present); Social Sciences Citation Index (1900-present); Arts & Humanities Citation Index (1975-present) ; Conference Proceedings Citation Index- Science (1990-present) ; Conference Proceedings Citation Index- Social Science & Humanities (1990-present) ; Emerging Sources Citation Index (2019-present)

<https://www.webofscience.com/wos/woscc/summary/3ce071e0-a7a1-4988-9ee6-cf97be7f70dd-0168cfb2c5/relevance/1>

ID Search Hits

#1 (TI=(("physical activit*" or sport* or walking or running or jogging or swimming or exercise* or exercising or "strength training*" or "resistance training*" or "weight training*" or aerobics or "physical endurance*" or "physical fitness" or "physical strength*" or "physical conditioning*" or "physical training*" or "active transport*" or commut* or "travel mode*" or biking or bike or bicycl* or (recreation* NEAR/2 (activit* or sport*)) or athletics or badminton or ballet or baseball or basketball or biathlon or bodybuilding or boxing or canoeing or cross-fit or dancing or dance or fencing or football or golf or golfing or gymnast* or gym or hiking or hockey or horse-riding or horseback-riding or judo or jiu-jitsu or ju-jitsu or karate or kayaking or kung-fu or marathon or "martial art*" or orienteering or padel or pilates or rowing or rugby or skating or skateboarding or skiing or soccer or softball or surfing or squash or taekwondo or Tai-Chi or Tai-Ji or tennis or triathlon or volleyball or water-polo or waterpolo or wrestling or yoga or zumba))) OR AK=(("physical activit*" or sport* or walking or running or jogging or swimming or exercise* or exercising or "strength training*" or "resistance training*" or "weight training*" or aerobics or "physical endurance*" or "physical fitness" or "physical strength*" or "physical conditioning*" or "physical training*" or "active transport*" or commut* or "travel mode*" or biking or bike or bicycl* or (recreation* NEAR/2 (activit* or sport*)) or athletics or badminton or ballet or baseball or basketball or biathlon or bodybuilding or boxing or canoeing or cross-fit or dancing or dance or fencing or football or golf or golfing or gymnast* or gym or hiking or hockey or horse-riding or horseback-riding or judo or jiu-jitsu or ju-jitsu or karate or kayaking or kung-fu or marathon or "martial art*" or orienteering or padel or pilates or rowing or rugby or skating or skateboarding or skiing or soccer or softball or surfing or squash or taekwondo or Tai-Chi or Tai-Ji or tennis or triathlon or volleyball or water-polo or waterpolo or wrestling or yoga or zumba)) 1,031,806

#2 (TI=(("Body image" or Connectedness or "Emotional health" or Happiness or "Health literacy" or "Life quality" or "Life satisfaction" or "Mental health" or Optimism or (Perceived NEAR/3 (appearance* or competence* or fitness or mood* or health-status)) or (Positive NEAR/3 (affect* or mood*)) or "Psychologic* health" or "Psychosocial health" or "Quality of life" or Relaxation or Resilience or "Satisfaction with life" or Self-confidence or Self-concept or Self-efficacy or Self-esteem* or Self-image or Self-perception* or "Sense of belonging" or "Social health" or Vitality or Well-being or Wellbeing))) OR AK=(("Body image" or Connectedness or "Emotional health" or Happiness or "Health literacy" or "Life quality" or "Life satisfaction" or "Mental health" or Optimism or (Perceived NEAR/3 (appearance* or competence* or fitness or mood* or health-status)) or (Positive NEAR/3 (affect* or mood*)) or "Psychologic* health" or "Psychosocial health" or "Quality of life" or Relaxation or Resilience or "Satisfaction with life" or Self-confidence or Self-concept or Self-efficacy or Self-esteem* or Self-image or Self-perception* or "Sense of belonging" or "Social health" or Vitality or Well-being or Wellbeing)) 835,246

#3 TS=(adolescen* or teen* or youth* or juvenil* or "young adult*" or "early adulthood" or "young person*" or "young individual*" or "young people*" or "young population*" or girl or girls or boy or boys or student or students or "secondary schooler*" or "middle schooler*" or "high schooler*" or highschooler* or ((secondary or middle or high) NEAR/1 (school or schools)) or highschool or highschools) 2,501,314

#4 TS=("thematic analys*" OR "content analys*" OR "focus group*" OR ethnograph* OR ethnograf* OR etnograf* OR "field stud*" OR phenomenolog* OR narration* OR narrative OR "qualitative stud*" OR "qualitative analys*" OR "qualitative research*" OR "qualitative method*" OR multimethodolog* OR "mixed method*" OR observation* OR "grounded theory" OR "audio recording*" OR "tape recording*" OR audiotape* OR ((semi-structured OR semistructured OR unstructured OR informal OR in-depth OR indepth OR face-to-face OR structured OR guide*) AND (interview* OR discussion* OR questionnaire*)) OR trial* OR random* OR rct OR prospective* OR retrospective* OR longitudinal* OR cohort* OR observation* OR quasi-experimental OR experimental OR before-and-after OR pre-post OR intervention* OR follow-up* OR followup) 15,608,670

#5 #1 AND #2 AND #3 AND #4 4,091

#6 TI=(anorexia or arrhythmia* or cancer* or "cardiovascular disease*" or chemotherap* or chronic or cerebrovascular or concussion or "cystic fibrosis" or diabet* or disorder* or disable* or disabilit* or disease* or doping or epilepsy or illness* or implant* or injur* or "myocardial infarct*" or "multiple sclerosis" or neuromuscular* or neoplas* or orthodont* or orthopedic or orthopaedic or pain or patient* or postpartum or pregnan* or psychiatr* or rehabilitat* or screening or sick or sickness or stroke or surg* or survivor* or syndrome* or suicid* or therap* or transplant*) or TI=(cross-section*) 12,409,788

#7 #5 NOT #6 3,236

#8 #7 AND DT=(article OR early access) 2,895

#9 #8 AND PY=(2009-2025) 2,694

**Citation searching**

Five additional articles, not identified through database searches, were retrieved via citation searching (Forward citation searching was conducted using the tool Citationchaser <https://estech.shinyapps.io/citationchaser>; backward citation searching was performed manually by reviewing the reference lists of included studies):

Rauscher, L., Kauer, K., & Wilson, B. D. (2013). The healthy body paradox: Organizational and interactional influences on preadolescent girls’ body image in Los Angeles. *Gender & Society*, *27*(2), 208-230.

Jago, R., Edwards, M. J., Sebire, S. J., Tomkinson, K., Bird, E. L., Banfield, K., ... & Blair, P. S. (2015). Effect and cost of an after-school dance programme on the physical activity of 11–12 year old girls: The Bristol Girls Dance Project, a school-based cluster randomised controlled trial. *International Journal of Behavioral Nutrition and Physical Activity*, *12*, 1-15.

Schaillée, H., Theeboom, M., & Skille, E. (2017). Adolescent girls’ experiences of urban dance programmes: A qualitative analysis of Flemish initiatives targeting disadvantaged youth. *European Journal for Sport and Society*, *14*(1), 26-44.

Ullrich-French, S., Cole, A. N., & Montgomery, A. K. (2016). Evaluation development for a physical activity positive youth development program for girls. *Evaluation and program planning*, *55*, 67-76.

Marshall, J., Kelly, P., & Niven, A. (2019). “When I go there, I feel like I can be myself.” Exploring programme theory within the wave project surf therapy intervention. *International Journal of Environmental Research and Public Health*, *16*(12), 2159.

# Supplementary Section S6: Detailed information about title-abstract-screening with ASReview

The SAFE Procedure in ASReview (Boetje & van de Schoot, 2024) is a four-step-process by providing “a practical and conservative set of stopping heuristics that offers a clear guideline for determining when to end the active learning process in screening software like ASReview” (Boetje & van de Schoot, 2024, p. 1). The application of this procedure for our title-abstract-screening is presented below. We used ASReview for the first search with a total of 7,129 records, but not for the update search with additional 1,558 records.

1. S = Screen random set

As suggested, we randomly screened 1% of the total numbers of records (total number of records in the training set, t = 72) and found 3 relevant records. Thus, the Fraction of Relevant Records in the training set (FRR_t) are 3 / 72 = 0.0417. Based on that, we can estimate number of Relevant Records in the total dataset (RR_T): 0.0417*7129 = 298.

2. A = Active Learning

With the labelled records from step 1, we started with the complete data set (T = 7129) and with a simple model (classifier: Naive Bayes; feature extractor: TF-IDF) to find as many relevant records as possible. The stopping rules encompass screening (1) and marking all previously marked key papers as relevant; (2) at least twice the number of relevant records (RR_T), estimated in step one (here: 2*298 = 696); (3) a minimum of 10% of the total data set (here 7129*0.1 = 713); (4) No relevant records have been identified in the last 50 records. Two screeners (LL, RH) conducted this step independently.

3. F = Find more using Deep Learning

In this step, another algorithm (deep learning) was used to find more additional records, which were might missed in step. The prior knowledge was the labelled records from steps 1 and 2. As proposed, we used the deep learning model (classifier: sentence bert; feature extractor: fully connected neural network). The stopping rule here is set by no extra relevant records are identified in the last 50 records. Two screeners (LL, RH) conducted this step independently.

4. E = Evaluate Quality

The aim here is to identify any wrongly excluded records from the previous steps. Therefore, the 10 lowest and highest ranked records were used as the prior knowledge. In doing so, one screener checked the previously excluded records from the other screener, and vice versa. Also here, the stopping rule is no extra relevant records are identified in the last 50 records.

After conducting these 4 steps, all the records are labelled as relevant from both screeners were compared. Discrepancies were discussed to reach consensus.

# Supplementary Table S7: Extended Mixed-Methods Appraisal Tool (MMAT)

As suggested by Hong et al. (2018), the criteria of the MMAT has to be discussed within the author group and can be adapted in relation to the specific investigation. This helps the assessers to grade the included studies precisely. In the following, the items, explanations by the others and our extensions are presented:

*For Qualitative studies*

| *Questions* | 1.1. Is the qualitative approach appropriate to answer the research question? | 1.2. Are the qualitative data collection methods adequate to address the research question? | 1.3. Are the findings adequately derived from the data? | 1.4. Is the interpretation of results sufficiently substantiated by data? | 1.5. Is there coherence between qualitative data sources, collection, analysis and interpretation? |
| --- | --- | --- | --- | --- | --- |
| *Explanations by Hong et al.* | MMAT Criteria Manual:  The qualitative approach used in a study (see non-exhaustive list on the left side of this table) should be appropriate for the research question and problem. For example, the use of a grounded theory approach should address the development of a theory and ethnography should study human cultures and societies | MMAT Criteria Manual:  This criterion is related to data collection method, including data sources (e.g., archives, documents), used to address the research question. To judge this criterion, consider whether the method of data collection (e.g., in depth interviews and/or group interviews, and/or observations) and the form of the data (e.g., tape recording, video material, diary, photo, and/or field notes) are adequate. Also, clear justifications are needed when data collection methods are modified during the study | MMAT Criteria Manual:  This criterion is related to the data analysis used. Several data analysis methods have been developed and their use depends on the research question and qualitative approach. For example, open, axial and selective coding is often associated with grounded theory, and within- and cross-case analysis is often seen in case study. | MMAT Criteria Manual:  The interpretation of results should be supported by the data collected. For example, the quotes provided to justify the themes should be adequate. | MMAT Criteria Manual:  There should be clear links between data sources, collection, analysis and interpretation. |
| *Extensions by the authors* | Does the chosen method (e.g., case study, grounded theory, qualitative description) align with the specific research question? Additionally, is an epistemological approach mentioned that clarifies the perspective or worldview from which the phenomenon is examined?  If there is no explanation for the chosen approach in the qualitative method (“qualitative research approach”) and no mentioning of the epistemological approach, the criterion is viewed as not fulfilled. | Expanding on 1.1, now focus on the concrete technique for investigation: Do the interviews, for example, provide information on the research question, or would other techniques (e.g., quantitative) be more suitable?  Qualitative research is about looking behind the scenes, understanding, delving deeper, etc.(“subject-method-fit”) | Does the evaluation method (thematic/content analysis) fit the procedure? Were the categories/statements checked by two people or is there no information on how this was done?  Was the analysis properly chosen and reported? | Also similar to 1.3 but less about the kind of analysis and more about whether the conclusions are logical and permissible. Are the conclusions drawn from the data, correct? Are the conclusions backed with quotes? And is referred to quality criteria (e.g., Lincoln & Guba)? Are the reported results comprehensible and properly elaborated? | Is the whole approach, analysis, reporting and interpretation coherent?  Would one come to the same conclusion with the data available? |

*For Quantitative randomized controlled trials*

| Questions | 2.1. Is randomization appropriately performed? | 2.2. Are the groups comparable at baseline? | 2.3. Are there complete outcome data? | 2.4. Are outcome assessors blinded to the intervention provided? | 2.5 Did the participants adhere to the assigned intervention? |
| --- | --- | --- | --- | --- | --- |
| *Explanations by Hong et al.* | In a randomized controlled trial, the allocation of a participant (or a data collection unit, e.g., a school) into the intervention or control group is based solely on chance. Researchers should describe how the randomization schedule was generated. A simple statement such as ‘we randomly allocated’ or ‘using a randomized design’ is insufficient to judge if randomization was appropriately performed. Also, assignment that is predictable such as using odd and even record numbers or dates is not appropriate. At minimum, a simple allocation (or unrestricted allocation) should be performed by following a predetermined plan/sequence. It is usually achieved by referring to a published list of random numbers, or to a list of random assignments generated by a computer. Also, restricted allocation can be performed such as blocked randomization (to ensure particular allocation ratios to the intervention groups), stratified randomization (randomization performed separately within strata), or minimization (to make small groups closely similar with respect to several characteristics). Another important characteristic to judge if randomization was appropriately performed is allocation concealment that protects assignment sequence until allocation. Researchers and participants should be unaware of the assignment sequence up to the point of allocation. Several strategies can be used to ensure allocation concealment such relying on a central randomization by a third party, or the use of sequentially numbered, opaque, sealed envelopes (Higgins et al., 2016) | Baseline imbalance between groups suggests that there are problems with the randomization. Indicators from baseline imbalance include: “(1) unusually large differences between intervention group sizes; (2) a substantial excess in statistically significant differences in baseline characteristics than would be expected by chance alone; (3) imbalance in key prognostic factors (or baseline measures of outcome variables) that are unlikely to be due to chance; (4) excessive similarity in baseline characteristics that is not compatible with chance; (5) surprising absence of one or more key characteristics that would be expected to be reported” (Higgins et al., 2016, p. 10). | Almost all the participants contributed to almost all measures. There is no absolute and standard cut-off value for acceptable complete outcome data. Agree among your team what is considered complete outcome data in your field and apply this uniformly across all the included studies. For instance, in the literature, acceptable complete data value ranged from 80% (Thomas et al., 2004; Zaza et al., 2000) to 95% (Higgins et al., 2016). Similarly, different acceptable withdrawal/dropout rates have been suggested: 5% (de Vet et al., 1997; MacLehose et al., 2000), 20% (Sindhu et al., 1997; Van Tulder et al., 2003) and 30% for a follow-up of more than one year (Viswanathan and Berkman, 2012). | Outcome assessors should be unaware of who is receiving which interventions. The assessors can be the participants if using participant reported outcome (e.g., pain), the intervention provider (e.g., clinical exam), or other persons not involved in the intervention (Higgins et al., 2016). | To judge this criterion, consider the proportion of participants who continued with their assigned intervention throughout follow-up. “Lack of adherence includes imperfect compliance, cessation of intervention, crossovers to the comparator intervention and switches to another active intervention.” (Higgins et al., 2016, p. 25). |
| *Extensions by the authors* | 1. The randomization has to be well justified and not just mentioned that it was randomized.  2. It should be blinded (in our context often difficult) – higher focus on criterion 1. | :  - Group size (EG & CG) should not vary to much  - some differences are expected by chance but not too much  🡪ideally no baseline imbalance in relevant variables and group sizes, if so:  1) if randomization was done well, no criticism  2) if big imbalance and no good randomization 🡪 not fulfilled | Especially about attrition: how many participants dropped out from pre- to post-test?  We use a benchmark of 20% (like Stern et al. (2019) and Higgins & Green (2011)) | For us only about Participants and not about investigators (cf. PEDRO-scale). Were the participants blinded?  Especially about the control group, were they aware of being the CG and not receiving an intervention?  For example, if there is nothing written about the CG receiving any kind of information material that led them to believe that they were receiving an intervention 🡪 criteria not fulfilled | For example, how often did participants take part in a 10-week intervention?  Derived from ROB 2, Box 7:  1. was there non-adherence to the assigned intervention regimen that could have affected participants' outcomes?  🡪Consider available information on the proportion of study participants who continued with their assigned intervention throughout follow up, and answer ‘Yes’ or ‘Probably yes’ if the proportion who did not adhere is high enough to raise concerns.  🡪 According to Jeffrey et al. (2000) we take a value of 70%, so if adherence > 70% a YES anyway  2. if there is less than 70% adherence, were statistical methods used to account for this deviation?  🡪 ROB 2 would suggest ‘inverse probability weighting’ (which probably does not occur in our case), in our case rather: were separate analyses according to adherence taken into account, e.g. was this variable included (as a moderator)? If yes, then a YES  🡪if nothing was mentioned about adherence, the criterion is not fulfilled anyway |

References:

Higgins, J. P. T., & Green, S. (Eds.). (2011). Cochrane handbook for systematic reviews of interventions (Version 5.1.0, updated March 2011). The Cochrane Collaboration.

Jeffery, R. W., Epstein, L. H., Wilson, G. T., Drewnowski, A., Stunkard, A. J., & Wing, R. R. (2000). Long-term maintenance of weight loss: current status. *Health psychology*, *19*(1S), 5.

Sterne, J. A. C., Savović, J., Page, M. J., Elbers, R. G., Blencowe, N. S., Boutron, I., Cates, C. J., Cheng, H. Y., Corbett, M. S., Eldridge, S. M., Emberson, J. R., Hernán, M. A., Hopewell, S., Hróbjartsson, A., Juni, P., Kirkham, J. J., Lasserson, T., Li, T., McAleenan, A., ... Higgins, J. P. T. (2019). RoB 2: A revised tool for assessing risk of bias in randomized trials. BMJ, 366, l4898. <https://doi.org/10.1136/bmj.l4898>

*For quantitative non-randomized*

| Questions | 3.1. Are the participants representative of the target population? | 3.2. Are measurements appropriate regarding both the outcome and intervention (or exposure)? | 3.3. Are there complete outcome data? | 3.4. Are the confounders accounted for in the design and analysis? | 3.5. During the study period, is the intervention administered (or exposure occurred) as intended? |
| --- | --- | --- | --- | --- | --- |
| *Explanations by Hong et al.* | MMAT Criteria Manual:  Indicators of representativeness include: clear description of the target population and of the sample (inclusion and exclusion criteria), reasons why certain eligible individuals chose not to participate, and any attempts to achieve a sample of participants that represents the target population | MMAT Criteria Manual:  Indicators of appropriate measurements include: the variables are clearly defined and accurately measured; the measurements are justified and appropriate for answering the research question; the measurements reflect what they are supposed to measure; validated and reliability tested measures of the intervention/exposure and outcome of interest are used, or variables are measured using ‘gold standard’. | MMAT Criteria Manual:  all the participants contributed to almost all measures. There is no absolute and standard cut-off value for acceptable complete outcome data. Agree among your team what is considered complete outcome data in your field (and based on the targeted journal) and apply this uniformly across all the included studies. For example, in the literature, acceptable complete data value ranged from 80% (Thomas et al., 2004; Zaza et al., 2000) to 95% (Higgins et al., 2016). Similarly, different acceptable withdrawal/dropouts rates have been suggested: 5% (de Vet et al., 1997; MacLehose et al., 2000), 20% (Sindhu et al., 1997; Van Tulder et al., 2003) and 30% for follow-up of more than one year (Viswanathan and Berkman, 2012). | MMAT Criteria Manual:  Confounders are factors that predict both the outcome of interest and the intervention received/exposure at baseline. They can distort the interpretation of findings and need to be considered in the design and analysis of a non-randomized study. Confounding bias is low if there is no confounding expected, or appropriate methods to control for confounders are used (such as stratification, regression, matching, standardization, and inverse probability weighting). | MMAT Criteria Manual:  For intervention studies, consider whether the participants were treated in a way that is consistent with the planned intervention. Since the intervention is assigned by researchers, consider whether there was a presence of contamination (e.g., the control group may be indirectly exposed to the intervention) or whether unplanned co-interventions were present in one group (Sterne et al., 2016). For observational studies, consider whether changes occurred in the exposure status among the participants. If yes, check if these changes are likely to influence the outcome of interest, were adjusted for, or whether unplanned co-exposures were present in one group (Morgan et al., 2017) |
| *Extensions by the authors* | 1) Is the target population described?  2) Are there inclusion and exclusion criteria?  3) Do the criteria match the target population?  4) Was an adequate attempt made to recruit a representative sample?  🡪 inclusion and exclusion criteria clearly the most important, if not 🡪 criteria not fulfilled, otherwise compensable | 1) Are the dependent variable defined and described?  2) Are the dependent variable adequate to answer the research question?  3) Are the dependent variables validated measures and if not are there information about reliability/variability?  🡪 If they didn’t use validated items/questionnaires and they were not explicitly analyzed 🡪 not fulfilled | Especially about attrition: How many participants dropped out from pre- to post-test?  We use a benchmark of 20% (like Stern et al. (2019) and Higgins & Green (2011)) | 1. Are there confounders  that can have an influence on the outcomes? For us, these are typically gender and, depending on the study, education level/SES. Also, the baseline level and, depending on this, also the attendance at interventions, if it scatters.  2. Are these confounders considered, either by the study design or by statistical methods (included in the model / separate analyses)?  🡪If nothing is taken into account, then a clear no  🡪If baseline imbalance exists and nothing is done about it, then also a no (logically only for studies that have control and experimental groups)  🡪yes, if there is no baseline imbalance or it is taken into account accordingly and if other confounders, at least gender, are taken into account | Did the Intervention go according to plan or were there Changes during the Interventions that could have impacted the Outcomes? (probably rarely a problem except changes or adaptions are explicitly reported)  In Intervention studies also note adherence to the intervention (how often did they participate in the intervention?)  If reported, benchmark of 70%.  🡪 If there is no mention of anything devious in the procedure or general study process, we give a YES. But if nothing at all is mentioned about the procedure (including the content of the intervention), then a NO |

References:

Higgins, J. P. T., & Green, S. (Eds.). (2011). Cochrane handbook for systematic reviews of interventions (Version 5.1.0, updated March 2011). The Cochrane Collaboration.

Sterne, J. A. C., Savović, J., Page, M. J., Elbers, R. G., Blencowe, N. S., Boutron, I., Cates, C. J., Cheng, H. Y., Corbett, M. S., Eldridge, S. M., Emberson, J. R., Hernán, M. A., Hopewell, S., Hróbjartsson, A., Juni, P., Kirkham, J. J., Lasserson, T., Li, T., McAleenan, A., ... Higgins, J. P. T. (2019). RoB 2: A revised tool for assessing risk of bias in randomized trials. BMJ, 366, l4898. https://doi.org/10.1136/bmj.l4898

# Supplementary Section S8: Information about the classification of the PMH outcomes

Based on the understanding of PMH (cf. Keyes, 2007; Lehnert et al., 2012; Park et al., 2023 ; Sudeck et al., 2023), two authors [LL, JS] classified every outcome from the original quantitative studies into to the following domains, and within the domains, into the following features:

| General, emotional & psychological well-being | | physical well-being | | social well-being | |
| --- | --- | --- | --- | --- | --- |
| Experiential features | Reflective features | Experiential features | Reflective features | Experiential features | Reflective features |

In doing so, the authors further developed and subdivided additional categories (see Figure 4 in the manuscript) based on the following reflections:

For general, emotional & psychological well-being: The well-being aspects were further differentiated in general or superordinated aspects, as well as experiential and reflective aspects. Within the reflective aspects, satisfaction and functioning in general, self-esteem/-concept (based on Craven & Marsh, 2008) and self-efficacy were further subdivided.

For physical well-being: Within the physical well-being domain, general, experiential, and reflective aspects were subdivided. However, there were no experiential aspects of well-being present in the studies included. And for the reflective category, based on the understanding of the physical self-concept (Dreiskaemper et al., 2022; Shavelson et al., 1976), we further differentiated aspects of general self-concept/-esteem, physical appearance and perceived capabilities.

For social well-being: Similar to the other domains of well-being, social well-being is divided into general, experiential and reflective aspects, and the latter is further subdivided into satisfaction with social relationships, social self-concept and social self-efficacy.

# Supplementary Table S9: Detailed information per study

Detailed study characteristics (abbreviations: NA = not available; RCT = randomized controlled trial; y = years; EG = Experimental group; CG = control group; E = Environment; QUAN = quantitative; QUAL = qualitative):

| Authors Year  Country | Title of Article | Research aim | Study design | Type of sample | Sample size (n)  Sex ratio  Age range (mean) | Contextual factors of LTPA (cf. Vella et al., 2023): | Quantitative factors of LTPA  (length of exposure/intervention; Frequency of sessions per week) | Detailed information about the intervention / exposure | Outcomes measured / qualitatively collected | Analysis strategy | Main findings relevant for this review |
| --- | --- | --- | --- | --- | --- | --- | --- | --- | --- | --- | --- |
| Abbott et al.  2014  Australia | Effects of Home Access to Active Videogames on Child Self-Esteem, Enjoyment of Physical Activity, and Anxiety Related to Electronic Games: Results from a Randomized Controlled Trial | Aiming to investigate the effects of Active Video Games (AVG) on overall perception of self, enjoyment of physical activity, and anxiety toward electronic game use in 10-12-year-old children and to compare them with the effects of home access to traditional games or no access to electronic games. | Crossover RCT | General population | n = 66;  Sex ratio: each 33 (50%) female and male;  age range 10-12y;  mean age 11.3y | Type: Active Videogames;  Setting: at home;  Physical E: Indoor at home;  Social E: NA;  Delivery: Self-organized | Length: 8 weeks per condition;  Frequency: Not controlled (equipment was available);  Intensity: NA;  Duration: different from <30min to 1-2h | 3 conditions of AVG per participants for 8 weeks: no games, traditional games, active games.  Participants selected 6 games per condition, played via Playstation2 with Eyetoy. | Self-esteem, measured by  Harter’s Self-Perception Profile for Chidlren (SPPC) with a general measure of self-estem and 5 subscales: scholastic competence, social acceptance, athletic competence, physical competence, behavioral competence | Mixed-model repeated-measures analyses contrasting two conditions | No significant changes or effects/differences for all scales of the SPPC and between the conditions (p < .05) |
| Bahram et al.  2014  Iran | Aerobics, Quality of Life, and Physiological Indicators of Inactive Male Students’ Cardiovascular Endurances, in Kashan | The study examined the effect of an eight-week aerobic exercise on the quality of life and physiological indicators of cardiovascular endurance in inactive adolescents. | RCT  (inactive control group) | At-risk (physically inactive) | n = 30;  only male;  age range: NA;  mean age: 17.5y | Type: indoor running (treadmill)  Setting: community-based;  physical E: indoor sports facility  Social E: NA;  Delivery: coach-led | Length: 8 weeks  Frequency: 3x;  Intensity: NA (aerobic);  Duration: 30-50min | Over 8 weeks students were exposed to aerobic exercise running intervention whilst the control group didn’t receive any kind of intervention.  Warm-up and cool-down included, principle of increasing overload for the main part. | Quality of Life (WHOQOL-26) with 4 dimensions: physical health, mental health, social relations, environmental health | Within group comparison with paired t-tests (p-level .05) | Age, weight, height and BMI don't differ between CG & EG (p > .05). Significant positive effects of the intervention group (pre-post) for all dimensions of quality of life (p < .001) while no pre-post effects were found in the control group. Analyses don't consider between-group and interaction effects, however, baseline values don’t seem to differ. |
| Beaulac et al.  2011  Canada | ‘Bigger than hip-hop?’ Impact of a community-based physical activity program on youth living in a disadvantaged neighbourhood in Canada | The research aim was to evaluate the perceived impact of a weekly hip-hop dance intervention on the psychological, social and physical well-being of adolescents. | Qualitative (single interviews with participants) | At-risk (socially disadvantaged) | N = 14;  Sex ratio: 11 (79%) female, 3 (21%) male;  Age range: 11-16y;  Mean age: NA | Type: Hip-Hop dancing;  Setting: community-based;  Physical E: indoor sport facility;  Social E: in a group with interactions;  Delivery: coach-led | Length: 13 weeks;  Frequency: 1x;  Intensity: NA;  Duration: 75min | The adolescents participated in a free 13-week program. Two formats were offered in response to community consultation, girls-only and co- ed, from each format were 7 participants interviewed. | Single interview within 2 weeks after the intervention. Open and non-leading questions (e.g., ‘Tell me how, if at all, the hip-hop dance program has affected you?’).  (Focus group interviews with parents and program personnel were not considered in this review) | Content and theme analysis according to Pope et al. (2000) | reported outcomes in relevant categories for this review:  1. physical well-being: improved physical fitness and sports competence.  2. psychological well-being: most common benefit was improved self-confidence, sense of accomplishment promoted self-confidence. Partly improved mood (happiness); partly improved self-discipline and goal setting  3. Relationships: increase in new relationships, partly improved child-parent-relationship. |
| Berger et al.  2009  USA | Effects of yoga on inner-city children's well-being: a pilot study | The objective was to examine the effect of yoga on well-being of inner-city children. | Quantitative non-randomized longitudinal studies with a control group | At-risk (disadvantaged urban youth) | N = 71  Sex ratio: 47 (66%) female, 24 (24%) male;  Age range: 9-11y;  Mean age: 10.3y | Type: yoga  Setting: school-based  Physical E: NA  Social E: in a group, but everybody for her-/himself  Delivery: coach-led | Length: 12 weeks  Frequency: 1x  Intensity: NA  Duration: 60min | The yoga group attended a 12 week after-school program that introduced yoga for all students instead of other physical activities, while the non-yoga group attended a program offering other, self-chosen physical activities. | self-perception: physical appearance & global self-esteem  via two subdimension of the Harter's Self-Perception Profile for Children (SPPC): physical appearance & global self-worth | T-tests and ANCOVA considering baseline values | No significant pre-post effects from t-tests and ANCOVA for the yoga group and control group (both groups did not change in values) |
| Budhraja et al.  2025  India | “Change needs to start at home”: A reflexive thematic analysis of girl athletes’ and coaches’ experiences of body image in New Delhi, India | The study aims to investigate the experiences of Indian athletes and coaches in relation to body image in sports context | Qualitative (focus groups interviews) | General population | N = 12;  Sex ratio: only female;  Age range: 11-17y;  Mean age: NA | Type: football and netball;  Setting: community-based;  Physical E: NA;  Social E.: in a group as a team;  Delivery: coach-led | Length: NA  Frequency: NA  Intensity: NA  Duration: NA | Girls and coaches from sports clubs participated  in the focus groups | Semi-structured focus groups focusing on body image | Thematic Analysis | Three themes identified, only one relevant for this review (Hold On To Your Power, Be You): Due to sports club participation social (made friends) and mood-related aspects (felt happier) were promoted. |
| Carlin et al. 2025  Ireland and Northern Ireland | A process evaluation of the walking in ScHools (WISH) study using the RE-AIM framework | The purpose of this process evaluation was to examine what influenced engagement, motivation, and enjoyment in the WISH intervention, to identify obstacles to walking, and to gain insight into the perspectives of students, walk leaders, and teachers. | Mixed-method study:  QUAN Cluster RCT;  QUAL: focus group interviews) | General population | N_QUAN_: 589,  N_QUAL_: 66;  Sex ratio: only female;  Age range 12-14y; mean age: NA | Type: brisk walking;  Setting: school-based;  Physical E: outdoor at the school area;  Social E.: together with somebody else;  Delivery: peer-led | Length: 20 weeks  Frequency: differs, mean walks ca. twice a week  Intensity: NA  Duration: 10-15min | Participants were encouraged to take brisk walks during the school day during one school year | QUAN: Health-related quality of life (hrQoL) with Kidscreen-10;  QUAL: Open questions following a semi-structured topic guide | QUAN: descriptive data (median and IQR) per measurement point;  QUAL: thematic analysis with an inductive approach | QUAN: no change in hrQoL;  QUAL: perceived effects on mental well-being (e.g., happier) and social connections (e.g., establishing and reinforcing relationships) |
| Case & Christophe  2019  USA | Strategies for Improving Self-Efficacy: A Qualitative Analysis of Detroit’s Downtown Boxing Gym | The goal was to examine how a community-based youth development program, the Downtown Boxing Gym (DBG), fosters self-efficacy development in lower-income youth of color. | Qualitative  (focus group interviews) | At-risk  (socially disadvantaged (low SES or from low SES area)) | n = 49;  Sex ratio: 21 (43%) female, 28 (57%) male;  Age range: 8-20y;  Mean age: NA (majority between 12-19y) | Type: Boxing  Setting: community-based  Physical E: indoor sport facility  Social E: NA (in a gym)  Delivery: coach-led but could also be self-organized | Length: on average 1.5 years  Frequency: NA  Intensity: NA  Duration: NA | On average the participant were involved 1.5years in the gym at the time though it varied from a few weeks to several years. The focus groups were held twice with 3 years apart with different participants. | The semi-structured focus groups included four main topics: gym services, perceived impacts on participants, specific aspects of the gym that contributed to these effects, and suggestions for improvement. Participants were also encouraged to introduce new topics related to their gym experiences. | Thematic analysis (Braun & Clarke, 2006),  focus in categorizing was self-efficacy , | Students' narratives reflected increased self-efficacy from gym participation through three key mechanisms: mastery experiences (gaining control over tasks both in and outside the gym, especially in school), verbal persuasion (encouragement from coaches and peers fostering belief in their abilities), and vicarious experiences (observing role models succeed, boosting their own confidence). The gym's supportive community, described as a "family" with shared life experiences, further reinforced self-efficacy and social well-being, leaving students more confident in their emotional and academic capabilities. |
| Choukse et al.  2018  India | Effect of residential yoga camp on psychological fitness of adolescents: A cohort study | The goal was to evaluate a short term residential yoga intervention on psychological constructs in adolescents. | Quantitative non-randomized longitudinal studies without a control group | general population | n = 510;  Sex ratio: 180 (35%) female, 330 (65%) male;  Age range: 9-16y;  Mean age:12.05y | Type: yoga  Setting: residential yoga camp  Physical E: NA  Social E: in a group, but everybody for her-/himself;  Delivery: coach-led | Length: 10 days  Frequency: daily  Intensity: NA  Duration: 8h per day | The adolescents participated in a 10-day Yoga Intervention where they enrolled in a 8 hour class room yoga session which consisted of different yoga practices. Three different cohorts (batches) took part in the intervention with the same conditions at different points of time. | Self-concept with a general score (Hadley et al., 2008). | Paired sample t-tests for pre-post measurement (p-level .05) | No significant effects over time in self-concept. |
| Das et al.  2016  India | Influence of Yoga-Based Personality Development Program on Psychomotor Performance and Self-efficacy in School Children | This study analyses the changes in psychomotor performance and self-efficacy after a specially designed personality development yoga camp for school children. | Quantitative non-randomized longitudinal studies with a control group | general population | n = 420;  Sex ratio: 156 (37.14%) female,  264 (62.86%) male;  Age range: 11-16y;  Mean age: 13.4y | Type: yoga;  Setting: community-based;  Physical E: NA  (yoga camp);  Social E: in a group, but everybody for her-/himself;  Delivery: coach-led | Length: 10 days  Frequency: daily  Intensity: NA  Duration: 10h per day | The 10-day-intervention is called "Personality Development Camp" with 10h activities per day  consisting of mainly different forms of yoga (physical postures, voluntary regulated breathing, meditation, relaxation techniques, internal cleansing practices and reciting hymns). | Self-efficacy measured with the  self-efficacy questionnaire for children (SEQ-C) including three domains: academic, social, and emotional self-efficacy. | Repeated measures ANOVA (comparing values on day 1 and 10 for experimental and control group), p-level < .05 | Significant between group effect and significant interaction effect, as well as pairwise comparison with Bonferroni correction with an significant increase in the yoga group in all three self-efficacy scales, while not for the control group. However, the results need to be interpreted with caution due to different baseline values which were much lower in the yoga-group. |
| DeBate et al.  2009  USA | Changes in psychosocial factors and physical activity frequency among third- to eighth-grade girls who participated in a developmentally focused youth sport program: a preliminary study | This study evaluates changes in self-esteem, body image, commitment to PA and PA behaviors in girls who participated in Girls on the Run or Girls on Track programs. | Quantitative non-randomized longitudinal studies without a control group | general population | n = 432;  only female:  Age range: 11-15y  Mean age: NA *(Only the analysis for the 11-15-years-old was considered in this review, and not the analysis for the participants for 10 years of age and younger)* | Type: running;  Setting: after-school and community-based;  Physical E: NA;  Social E: in a group with interactions;  Delivery: coach-led | Length: 12 weeks  Frequency: 2x  Intensity: high  Duration: 90min | The curriculum of the program included integrated psycho-educational aspects, discussions etc. Every session was combined with a session topic which was discussed during warm-up and cool-down. | Self-esteem via Rosenberg Self-Esteem Scale (Rosenberg, 1965); body image via body size satisfaction (Schematic Figural Scale, child/adolescent version; Collins, 1994) | Paired t-test for pre-post measurement | For the age group 11-15 years they found significant positive effects for self-esteem and body size satisfaction with p < .001. For the whole sample which consisted of children aged 8-15 years, the effects remain significant if children participated once or twice in the program and became non-significant when participating three or more times. |
| Decarpentrie 2024  Madagascar | Extracurricular activities as a way to foster development for youth living in an extreme context: a basic psychological needs perspective in Madagascar | Testing if the participation in extracurricular activities of youth developing in an extreme context had an effect over time on their psychological well-being and the satisfaction and frustration of their basic psychological needs | Quantitative non-randomized study with inactive control group | At-risk population | N= 96;  Sex ratio: 41.7% female, 58.3% male;  Age range: 14-18y; mean age; 15.07y; | Type: football or basketball;  Setting: community-based;  Physical E: NA;  Social E: in a group as a team;  Delivery: coach-led | Length: 64 weeks  Frequency: twice a week  Intensity: NA  Duration: 90-120min | Besides a control group two experimental group (artistic and sports group), focusing on positive development | Self-esteem: self-esteem scale (Rosenberg, 1965)  life satisfaction: Satisfaction with Life Scale (Diener, 1984); vitality: Subjective Vitality Scale (Ryan & Frederick, 1997) | Mixed factorial ANOVA considering age, gender, and socio-economic status | Non significant pre-post-changes in any of the constructs in focus (only for basic psychological need variables) |
| Diaz-Hidalgo et al.  2024  Spain | Improvements in Jump Height, Speed, and Quality of Life through an 8-Week Strength Program in Male Adolescents Soccer Players | This study examines the improvement in physical fitness and quality of life of an 8-week strength training, specifically designed for 12 and 13 year old male adolescent soccer player. | Quantitative non-randomized longitudinal studies with a control group | General population  (physically active) | n = 32;  only male;  age range: 12-13y;  mean age: 12.7y | Type: resistance training;  Setting: community-based;  Physical E: NA  Social E: NA  Delivery: coach-led | Length: 12 weeks  Frequency: 2x  Intensity: high  Duration: 90min | EG: soccer practice + strength training 2x/week¨  CG: soccer practice | Health-related quality of Life by the Kiddo-Kinddl with six dimensions (Rajmil et al., 2004): emotional well-being, self-esteem, school welfare and interest, fun with peers, family well-being, and physical well-being. | General linear repeated measures model analysis with  pre-post measurement of control and experimental group | An additional strength training in youth soccer player impacts overall quality of life and the subdimension QoL self-esteem, however, with no consistent effects for the QoL-subdimensions emotional well-being, school, friends, family well-being and physical well-being. |
| Duberg et al.  2016  Sweden | "I feel free": Experiences of a dance intervention for adolescent girls with internalizing problems | The goal was to assess the experiences of girls with internalizing problems participating in an 8-month dance intervention | Qualitative - retrospective | At-risk population (described as mentally vulnerable/problematic (but no diagnosed disorder) | n = 24;  only female;  age range 14-19y;  mean age 16.5y | Type: dancing (dance styles varied);  Setting: rather community-based (but described as after school program);  Physical E: indoor sport facilities Social E: in a group with interactions;  Delivery: coach-led | Length: 8 months;  Frequency: 2x;  Intensity: moderate;  Duration: 75min | Content of the sessions: warm-up, dance practice and relaxation including a short massage in pairs and a short reflection. The internalizing problems were not discussed. | open questions/ approach and no concrete questions for specific types of mental health (e.g., “tell me about your experiences of participating in the dance intervention”) | Inductive content analysis | Dancing for enriched personal ressources in a non-jugdemental atmosphere and supportive togetherness leading to increased self-trust.  categories: main category “Finding embodied self-trust that opens new doors”. 5 generic categories: “An oasis from stress”, “Supportive togetherness”, “Enjoyment and Empowerment”, “Finding Acceptance and Trust in Own Ability”,  “Dance as Emotional Expression”. |
| Dubey  2011  India | Impact of yogic practices on some psychological variables among adolescents | Investigating the effect of Yoga Practice on self-concept, aggression, tolerance, Ahimsa, truthfulness, faith and fidelity of adolescents | non-randomized study with a control group | general population (no specific focus at the recruiting) | n = 50;  sex ratio: 20 (40%) females, 30 (60%) males;  age range 16-18y, mean NA | Type: Yoga;  Setting: NA;  Physical E: NA;  Social E: in a group, but everybody for her-/himself;  Delivery: coach-led | Length: 15 days  Frequency: 7x  Intensity: NA  Duration: 90min | EG: Yogic practice for 15days daily in the morning from 5:30 to 7:00 am; CG: no information | self-concept questionnaire (Saraswat, 1992) with 6 dimensions: physical, social, temperamental, educational, moral, intellectual | t-test with pre and post measurement | In the EG, all dimensions of the self-concept were significant: physical: p < .05 social: p < .01 temperamental: p < .05 educational: p < .01 moral: p < .01 intellectual: p < .01 In the control group (CG), no sign. differences were found (p > .05). With similar baseline levels and no effects for CG, we can assume that there is an intervention effect for yoga on self-concept, resp. all subcategories. |
| Duncan et al.  2009  UK | Effects of a 6-week circuit training intervention on body esteem and body mass index in British primary school children | Examining the effect of a 6-week circuit training intervention on body esteem and body mass index in primary school children. | RCT | general population (no specific focus at the recruiting) | n = 68;  sex ratio: 34 (50%) female and male; age range: 10-11y,  mean age NA | Type: circuit training (plyometrics);  Setting: school-based (after-/before-school);  Physical E: indoor sport facilities;  Social E: group-based;  Delivery: coach-led | Length: 6 weeks  Frequency: 2x  Intensity: high  Duration: 40min | EG: 6 week plyometric type circuit training (hops, skips, jumps);  CG: did not receive any training and wasn’t allowed to participate in any extracurricular sport/exercise during these 6 weeks. | Body Esteem Scale for Children (BES-C; Mendelson & White, 1982), a general score is reported | RM-ANOVA  Pre-post-retention measurement (6-week follow-up) | Compared to the CG, the body esteem of the EG improved significantly from pre to post-intervention. This effect did not sustain 6 weeks post-intervention. |
| Gerguz & Bayram  2023  Turkey | Effects of Yoga Training Applied with Telerehabilitation on Core Stabilization and Physical Fitness in Junior Tennis Players: A Randomized Controlled Trial | Investigating the effect yoga training applied with tele-rehabilitation on core stability strength, balance, flexibility, upper extremity stability, body awareness, and quality of life on 6-18 years old tennis players | RCT | General population (physically very active) | n = 40;  sex ratio: 20 (50%) female and male;  age range 6-18y,  mean age 12y | Type: yoga;  Setting: via zoom;  Physical E: at home;  Social E: NA;  Delivery: coach-led | Length: 8 weeks;  Frequency: 2x;  Intensity: NA;  Duration: 50min | The EG received tennis as usual plus the yoga intervention whilst the CG only played tennis as usual. | Body Awareness Questionnaire;  SF-36 for health-related quality of life (8 Dimensions: physical functioning, role physical, bodily pain, general health, vitality, social functioning, role emotional, and mental health | t-tests between and within groups | Significant positive effect for body awareness; conflicting reporting for vitality and mental health of the SF-36, ceiling effects for multiple dimensions, positive effects for bodily pain and social functioning, no effects/change in physical functioning |
| Godfrey et al.  2015  UK | The positive impact of structured surfing courses on the wellbeing of vulnerable young people | assessing the wellbeing outcomes and the influence of the wave project, to demonstrate any issues around delivery and follow-up and to apply validated, practical measures for data collection that are robust enough to offer valuable insights for commissioners and practitioners | Mixed methods design:  QUAN Quantitative non-randomized longitudinal study without control group;  QUAL retrospective (comments at the end of the post-intervention-survey | At-risk population (described as mentally vulnerable/ problematic, but no diagnosed disorder) | n = 84;  sex ratio: NA;  age range 8-18,  mean age NA | Type: surfing;  Setting: community-based;  Physical E: outdoor in nature;  Social E: group-based;  Delivery: coach-led | Length: 6 weeks;  Frequency: 1x;  Intensity: NA;  Duration: NA | Group of 10 clients and led by paid surf instructors and enough volunteers to provide one-to-one support; focus on bringing out of the comfort zone and positive experiences. | QUAN: 7 well-being aspects, partly validated items (e.g., from the Stirling Children’s Wellbeing Scale (SCWBS): positive outlook/functioning, resilience/self-esteem, emotional well-being, vitality, social well-being, social trust, physical health;  QUAL: open questions/ approach and no concrete questions for specific types of mental health (the participants were asked what made them feel different) | QUAN: pre-post-comparisons with paired t-tests;  QUAL: Thematic analysis | QUAN: significant pos. effects in all outcomes;  QUAL: 5 themes were identifiied: Resilience and achievement, Confidence, Happiness, fun and excitement, Friends, group support and feeling safe. |
| Gómez-Paniagua 2025  Spain | A Preliminary Study on the Effect of an Intervention Based on Green Exercise on Mental Health and Physical Fitness of Adolescents | Aiming to inverstigate the effects of a physical activity intervention in the natural environment on life satisfaction and self-perceived physical condition in adolescents | Quantitative non-randomized longitudinal study without a control group | General population | n = 420;  Sex ratio: 204 (48.6%) female,  216 (51.4%) male;  Age range: NA,  Mean age 12.96y | Setting: community-based;  Physical E: outdoor in nature;  Social E: in a group with interactions, but no clear team;  Delivery: coach-led | Length: 2 weeks;  Frequency: 2x per day;  Intensity: NA;  Duration: 210-240min | The adolescents who attended camps in the region  took part in a 12 day intervention consisting of nature activities | Life satisfaction with Satisfaction with life scale (SWLS) | Nonpara-metric  Wilcoxon test | Small but significant increase in life satisfaction in pre-post comparison |
| Greco et al.  2019  Italy | Karate as anti-bullying strategy by improvement resilience and self-efficacy in school-age youth | Examining the effect of a 12-week karate-based intervention on adolescents resilience and self-efficacy | RCT | General population (no specific focus at the recruiting) | n = 100;  sex ratio: 50 (50%) female and male,  age range 14-16y,  mean age 14.6y | Type: Karate;  Setting: school-based (after-/before-school);  Physical E: indoor;  Social E: group-based;  Delivery: coach-led | Length: 12 weeks;  Frequency: 1x;  Intensity: NA;  Duration: 90min | EG: Start with psycho-educational activities and physical warm-up, main part with karate; CG: wait-list control group | Self-efficacy: Self-Efficacy Questionnaire for Children (SEQ-C, with 3 subscales and a total score);  *Resilience: Child and Youth Resilience Measure (CYRM-28, with 3 subscales and a total score); | ANOVA including post-hoc analyses  Pre-post measurement | Social self-efficacy sign. positive effect (p < .001); emotional self-efficacy sign. positive effect (p < .001; *academic self-efficacy sign. positive effect (p < .001; total self-efficacy sign. positive effect (p < .05);  *Resilience: Overall sign. positive effect on total score and in every subscale (p < .05) |
| Hignett et al.  2018  UK | Evaluation of a surfing programme designed to increase personal well-being and connectedness to the natural environment among 'at risk' young people | They aimed to investigate if a 12-week surfing programme shows benefits in physical fitness, self-reported life and domain satisfaction, connectedness to nature and other life domains for children and young people excluded or at risk of being excluded from mainstream school. | Mixed methods, but only the quantitative part fits this review (quantitative non-randomized longitudinal study without a control group) | At-risk population  (described as mentally vulnerable/problematic, but no diagnosed disorder) | n = 58;  Sex ratio: 10 (17.2%) female, 48 (82.8%) male;  Age range 13-16y, mean age 14.25y  All sexes  (82.8%) males, (17.8) females | Type: Surfing;  Setting: School-based (after-/before-school);  Physical E: outdoor;  Social E: group-based;  Delivery: coach-led | Once a week  Intensity NA  Length of a session NA  Length: 12 weeks;  Frequency: 1;  Intensity: NA;  Duration:NA | Surfing lessons at the beach in groups of 12-20 children with two coaches including environmental awareness and sustainability inputs to foster the connected-ness to the nature. | Self-reported well-being: well-being section from the Youth version of the British Panel Household Survey (BPHS-Y).  Connectedness: Adaptation of rhe  ‘Inclusion of Nature in the Self’ (INS, Schultz, 2002) to other domains of connectedness. | Paired t-test  Pre-post measurement | Self-reported well-being:  No significant (p < .05) pre-post-differences in life satisfaction overall and in the specific domains, except for physical appearance with a positive change.  ConnectednessNo significant (p < .05) pre-post-differences in the connectedness domains family, world, beach, local nature, except for school with a positive change. |
| Jago et al.  2015  UK | Effect and cost of an after-school dance programme on the physical activity of 11–12 year old girls: The Bristol Girls Dance Project, a school-based cluster randomised controlled trial | Aiming to investigate the effectiveness and cost of an after-school dance intervention at increasing physical activity levels of girls aged 11-12. | RCT | General population | n = 571;  Female only;  Age range 11-12y,  mean age NA | Type: dancing;  Setting: after-school;  Physical E: indoor;  Social E: group-based;  Delivery: coach-led | Length: 20 weeks;  Frequency: 2x;  Intensity: NA;  Duration: 75min | EG: Up to 40 dance sessions with experienced instructors including principles of the self-determination theory;  CG: only provided data but did not participate in the intervention. | Self-esteem: Self Description Questionnaire (SDQ) II from Marsh (1992) with one 1 general measure;  Health-related Quality of Life (hrQoL): European Quality of Life-5 Dimensions (Wille et al., 2010) with one general measure used | Multi-variable mixed effects linear models: EG vs. CG at t1 & t2, controlled for baseline values (t0) for Self-esteem; Mann-Whitney-U test for hrQoL | Self-esteem: at t1 a sign. (< .001) negative between-group effect for EG, at t2 no sign. differences;  hrQoL: no sign. between-group effect at each time point. |
| Kernebone et al.  2022  Australia | Kicking goals: Exploring the experiences of girls who play Australian Rules football | Aiming to understand the experience which adolescent girls have who play Australian football | Qualitative - retrospective | General population | n = 6;  female only:  age range 11-17y, mean age 14y | Type: Australian rules football;  Setting: community-based;  Physical E: outdoor sport facilities;  Social E: team activity;  Delivery: coach-led | Frequency NA Intensity NA  Length of session NA  Length: NA;  Frequency: NA;  Intensity: NA;  Duration: NA | No information was provided for how long the girls were already playing. They were just questioned about their experiences. | open questions/ approach and no concrete questions for specific types of mental health. They designed the questions to be broad, open-ended and non-directive. | Thematic analysis | Playing football contributed to positive self-perception and self-esteem through general, social, and physical dimensions, supported by a nurturing environment of teammates, coaches, and family. A supportive team environment nurtured confidence and a sense of safety and belonging. Role models further strengthened self-esteem and resilience against gender stereotypes, turning adversity into empowerment and motivation. Thus, football became a context for personal growth, confidence, and self-identity development within a supportive and inspiring community. |
| Kwasky et al. (2018) USA | Yoga to Enhance Self Efficacy: An Intervention for At-risk Youth | Examining whether yoga can improve the self-efficacy and body core tone in at-risk adolescent female participants | Quantitative non-randomized longitudinal studies without a control group | At-risk population | n = 15 ;  female only;  age range 11-14y, mean age 12.21y | Type: yoga;  Setting: after-school;  Physical E: indoor;  Social E: group-based;  Delivery: coach-led | Length: 8 weeks;  Frequency: 2x;  Intensity: NA;  Duration: NA | The participants practiced yoga twice a week during 8-weeks with a certified yoga instructor | Self-Efficacy Questionnaire for Children (SEQ-C; Muris, 2001) with total score and the academic, social, and emotional domain. | Pre-post-retention (4 weeks) measures, with  Friedman's ANOVA | Sign. (p < .05) positive change only for social self-efficacy, but not for the total score and the other domains. |
| La’l-Kheirkhah et al.  2019  Iran | The Effect of a Yoga Program on "Health-Related Quality of Life" of Children in Residential Care Centers | Aiming to determine the effects of a 8-week yoga exercise program on health-related quality of life and all subscales in parentless and mal-parented children at residential care centers. | Quantitative non-randomized longitudinal studies without a control group | At-risk population  (Children in residential care centers) | n = 25;  sex ratio: 14 (56%) female, 11 (44%) male;  age range 8-13y,  mean age 11.36y | Type: yoga;  Setting: community-based;  Physical E: indoor (sports center);  Social E: group-based;  Delivery: coach-led | Length: 8 weeks;  Frequency: 5x;  Intensity: NA;  Duration: 45min | The 8-week intervention contained separate sessions for boys and girls and included among other exercises aerobic and positioning exercises. | Kids Screen Health-related Quality of Life Questionnaire (KS-HRQOLQ) with five subscales physical well-being, mental well-being, parental relations and self-autonomy, social supports and peers, school environment | Statistical method not directly reported (“Repeated measurement analysis”),  Pre-post-retention (+1 week & +4 weeks) | No significant (p < .05) changes over time except for the physical well-being dimension. However, under consideration of demographic variables the change for physical wellbeing is no longer significant. |
| Lai et al.  2021  China | Social resources for positive psychosocial health: Youths' narratives of a street dance performing arts program | Aiming to explore and identify the social processes that underlie a 12-month youth street dance performing arts program and its contribution to psychosocial health improvements | Qualitative – longitudinal | General population | n = 22;  sex ratio: 15 (68.2%) females,  7 (31.8%) males;  age range 13-18y, mean age 16y | Type: Street-dance;  Setting: community-based;  Physical E: NA;  Social E: group-based;  Delivery: coach-led | Length: 12 months;  Frequency: at least 1x;  Intensity: NA;  Duration: NA | The One-Year program consisted of sets of sequenced activities with weekly rehearsals and included seven public performances | One-to-one in-depth interviews; open, non-suggestive questions. Key questions included themes like personal changes, feelings towards the program, socialization experiences with instructors and contribution of the instructors to personal changes. | Thematic analysis | The program improved youth psychosocial health through mechanisms of social interaction and support. Key factors were happiness and increased sense of social competence. The shared goal of performance, common goals and trust as well as the engagement of the instructors facilitated prosocial attitudes and heightened self-efficacy. |
| Li et al.  2025  China | Effects of a 16-week dance intervention on loneliness and self-esteem in left behind children: a randomised controlled trial | Investigating the effectiveness of a dance intervention on loneliness and self-esteem in left-behind children through a 16-week randomised controlled trial | RCT | At-risk population (left-behind children) | n = 1263;  sex ratio: 627 (49.6%) female, 636 (50.4%) male;  Age range NA, mean age 10.03y | Type: dance;  Setting: school-based;  Physical E: indoor sports facility;  Social E: in a group with interactions, but no clear team  Delivery: coach-led | Length: 16 weeks;  Frequency: 5x;  Intensity: moderate;  Duration: 45 min | Latin dance intervention after mandatory school with two professional dance instructors | Self-esteem  Rosenberg’s Self Esteem (RSES) | Two-factor RM-ANOVA | No concrete values reported, in the manuscript is a positive pre-post-change in the experimental group reported |
| Lubans et al.  2010  Australia | The effects of free weights and elastic tubing resistance training on physical self-perception in adolescents | Aiming to investigate free weights and elastic tubing resistance training on physical self-perception in adolescents. | RCT | General population | n = 108;  sex ratio: 52 (48.1 %) female, 56 (51.9 %) male;  Age range NA, mean age 14.96y | Type: resistance training (free weights and elastic tubing);  Setting: school-based;  Physical E: indoor sports facility;  Social E: not clear if exercised alone or in pairs, but in a group;  Delivery: coach-led | Length: 8 weeks;  Frequency: 2x;  Intensity: NA;  Duration: 40-50min | 3 conditions free weights, elastic tube and control group which did not participate in any form of resistance training. The sessions were during lunch time but not during school hours with 2 sets of 10-12 repetitions on 10 exercises | physical self-perception: Adolescent version of the Physical Self-Perception Profile (PSPP: Fox & Corbin, 1989, 1990) with 5 subscales: sports competence, physical condition, strength, body attractiveness, and overall physical self-worth. | Paired t-test,  Pre-post measurement, separate analysis for females and males. | There were no significant (p < .05) effects found for boys. For girls there was only for the subdimension body attractiveness a sign. effect (p < .01) with an pre-post-test increase of d = 0.76 in the free weight group. |
| Luttenberger et al.  2024  Lebanon | A psychosocial bouldering intervention improves the well-being of young refugees and adolescents from the host community in Lebanon: results from a pragmatic controlled trial | Aiming to investigate the effects of bouldering intervention on psychosocial outcomes among refugee adolescents | Non-RCT | At-risk population (refugee experiences) | n = 156;  Sex ratio: 91 (58.3%) female, 65 (41.7%) male;  Age range: 14-19y; Mean age 16.05y | Type: Bouldering;  Setting: community-based;  Physical E: artifcial bouldering wall;  Social E: in a group with interactions, but no clear team;  Delivery: coach-led | Length: 8 weeks;  Frequency: 1x;  Intensity: NA;  Duration: 120 min | An 8-week psychosocial bouldering program with session-specific topics, led by trained climbing instructor and social worker. | Levantine Arabic version of the Warwick-Edinburgh Mental Well-Being Scale (WEMWBS); Arabic version of the General Self-Efficacy Scale (GSE) | Lnear mixed models | Sign. positive effect for WEMWBS, no or marginal effect on GSE |
| Marshall et al.  2019  UK | "When I Go There, I Feel Like I Can Be Myself." Exploring Programme Theory within the Wave Project Surf Therapy Intervention | Aiming to adopt a rigorous grounded theory approach to investigate the program theory underlying the Wave Project surf therapy intervention | Qualitative – retrospective | At-risk population (described as mentally vulnerable/problematic, but no diagnosed disorder) | N = 22;  Sex ratio: 14 (64%) females, (36%) males;  Age range 8-23y, mean age 14y | Type: surfing;  Setting: community-based;  Physical E: outdoor in nature;  Social E: group-based;  Delivery: coach-led (incl. peer-mentoring) | Length: 6 weeks + further club membership;  Frequency: 1x;  Intensity: NA;  Duration: 120-180min | 6-week course, attending weekly for two- to three-hour sessions of surfing. After that, participants joined a regular opt-in surf club that provides a continuation of surfing as a member. | semi-structured and opened interview with a very broad question about participant experiences and further open-ended and non-leading questions that would allow for exploration of participants’ experiences. | grounded theory approach with constant comparative analysis and memo writing | 3 types of categories were identified with the following subcategories which describe the surfing experience: antecedent categories (multiple challenge levels provided by waves, removing perceptions of failure/pressure within the group, physical and emotional support from peer mentors); core categories (self-selected pacing and progression while surfing, creation of emotional and physical safe space at the beach); and consequent categories (sense of mastery and accomplishment at learning a new skill, enjoying a sense of respite/escape at beach, and social connections with “surfer” peer group) |
| Mona et al. 2024  India | Safe Use of Screen Time Among Adolescents: A Randomized Controlled Study of the Efficacy of Yoga | Aiming to evaluate the impact of yoga to address excessive screen time use and health-related outcomes (depressive symptoms, anxiety, physical health problems, and well-being) among students | RCT | General population | n = 94;  Sex ratio; each 47 (50%);  Age range 13-17y; Mean age 13.5y | Type: Yoga;  Setting: school-based;  Physical E: indoor sport facilities;  Social E: in a group, but everybody for her-/himself;  Delivery: coach-led | Length: 12 weeks;  Frequency: 3x;  Intensity: NA;  Duration:45 min | Participants either took part in a 12-week yoga trial or an educational program on ditigal hygiene | Subjective well-being with the  WHO-5 Well-Being Index (WHO, 1998) | RM-ANOVA | Non-significant pre-post-change in the experimental group |
| Pereira et al.  2017  Portugal | Including educational dance in an after-school socio-emotional learning program significantly improves pupils’ self-management and relationship skills? A quasi experimental study. | Aiming to evaluate the efficacy of the Experiencing Emotions program on the socio-emotional skills of middle school pupils, and to determine the effects of the intervention on emotional, psychological and social well-being, and on the behavioral dimension of school engagement. | Quantitative non-randomized longitudinal studies with a control group | General population (but from low to medium socio-economic areas) | n = 83;  no information on sex ratio;  age range 9-13y, mean age 10.7y | Type: (educational) dancing ;  Setting: school-based;  Physical E: indoor;  Social E: group-based;  Delivery: coach-led | Length: 12 weeks;  Frequency: 1x;  Intensity: NA;  Duration:60min | Participant could choose edu cational dance class (EG) or handcraft activities (CG). EG: dancing with “moments of reflection”. | Mental Health Continuum-Short Form (MHC-SF; Keyes, 2002) with the three dimensions: emotional, psychological and social well-being. | ANCOVA  Pre-post measurement | There were no sign. (p < .05) effects for all three well-being domains in the ANCOVA for the group-time-interaction. Pre-post-increases in the EG occurred for psychological and emotional well-being, however, also due to the increase in the CG no significant change occurred. |
| Philipsson et al.  2013  Sweden | Cost-utility analysis of a dance intervention for adolescent girls with internalizing problems | Aiming to evaluate the cost-effective-ness of a additional dance program supporting usual school health services for adolescent girls with internalizing problems comparing to only usual school health services (health outcomes necessary to calculate health costs) | RCT | At-risk population (described as mentally vulnerable/problematic, but no diagnosed disorder) | n = 112;  Only female;  Age range 13-18, Mean age NA | Type: dancing;  Setting: NA;  Physical E: indoor;  Social E: group-based;  Delivery: coach-led | Length: 8 months;  Frequency: 2x;  Intensity: moderate;  Duration: 75min | EG: dance sessions consisted of different dance themes and in every session, there was a warm-up, creative group practice, dance routine and stretch. The session always ended with relaxation.  CG: continue living as usual | Quality of Life (QoL)  Health Utilities Index Mark 3 (HUI3), one score was used | Paired t-test  Pre-post-retention measurement  baseline, 4 month (during intervention), 8 month (end of the intervention), 12 month and 20 month ( = so many months after baseline). | QoL improved in the EG but never exceeded the values of the CG, as the EG baseline values already were lower, they only surpassed them 12 months post-intervention. The relative gain of QoL in the EG was significant in comparison to the CG but the effects overall are inclusive. |
| Rauscher et al.  2013  USA | The Healthy Body Paradox Organizational and Interactional Influences on Preadolescent Girls’ Body Image in Los Angeles | Aiming to explore how the girls on the run (GOTR) program influences the way girls understand and feel about their bodies by analyzing their perception in the context of institutionalized obsession with thinness and anti-fat bias. | Quantitative non-randomized longitudinal studies without a control group  (Mixed methods study, for our review only quantitative part fits to the inclusion criteria) | General population (no specific focus at the recruiting) | n = 138;  Only female;  Age range 8-14y, mean age 10.5y | Type: Running;  Setting: community-based;  Physical E: outdoor;  Social E: group-based;  Delivery: coach-led | Length: 12 weeks;  Frequency: 2y;  Intensity: vigorous;  Duration: 90min | The curriculum of the program included integrated psycho-educational aspects, discussions etc. Every session was combined with a session topic which was discussed during warm-up and cool-down. | Objectified Body Consciousness: Fredrickson et al.’s (1998) four-item Objectified Body Consciousness Scale;  Body Esteem:  Mendelson and White’s (1982) 20-item Body Esteem Scale | t-tests  pre-post measurement | The program had a significant (p < .001) but small positive effect on objectified body (d = 0.22) consciousness and body esteem (d = 0.28). |
| Rinaldo et al.  2016  Italy | Soccer training programme improved the body composition of pre-adolescent boys and increased their satisfaction with their body image. | Aiming to understand the body composition and body image perception of preadolescents boys by evaluating their anthropometric characteristics and body image perception after a 12-week soccer-training program. | Quantitative non-randomized longitudinal studies without a control group | General population | N = 36;  Only male;  Age 10y  (the 9-year-old boys were not considered in this summary because it is out of the scope for this review) | Type: football;  Setting: community-based;  Physical E: NA;  Social E: team activity;  Delivery: coach-led | Length: 12 weeks;  Frequency: NA;  Intensity: NA;  Duration: NA  4h per weeks | No detailed information about intervention but soccer practice only. | body image perception (Collins, 1991) | paired t-test  pre-post measurement | The programme had a significant (p < .05) positive effect on body image perception (feel-ideal-difference decreased). |
| Ryan et al. 2024  UK | A Community Perspective on Boxing, Well-being and Young People, | Aiming to explore what benefits, if any, that young people, and others in their community, associate with community boxing gyms | Qualitative – retrospective | At-risk population  (socially disadvantaged) | N = 9;  Only male;  Age range: 11-17y  Mean age: 13.25y | Type: boxing;  Setting: community-based;  Physical E: indoor sport facilities;  Social E: in a group with interactions, but no clear team sports;  Delivery: coach-led | Length: NA;  Frequency: NA;  Intensity: NA;  Duration: NA | Adolescents who were enrolled in a boxing gym were observed in the ethnographic study and interviewed. | No specific information about he interviews, they were interested in adolescents’ experiences in the community boxing gym | Thematic analysis | Three main themes identified: shift in how I see myself (e.g., more control of own actions, more agency); recursive learning process (e.g., self-efficacy through mastery experiences, embodied learning); social essence of a community gym (e.g., new family, sense of safety) |
| Schaillée et al.  2017  Belgium | Adolescent girls’ experiences of urban dance programmes: a qualitative analysis of Flemish initiatives targeting disadvantaged youth | Aiming to examine the perceived developmental benefits of a urban dance program by disadvantaged girls and to discover the social mechanisms under which these outcomes are generated. | Qualitative – longitudinal | At-risk population  (socially disadvantaged) | n = 25;  Only female;  Age range 11-19y, mean age 13.8y | Type: urban dancing;  Setting: community-based;  Physical E: NA;  Social E: group-based;  Delivery: coach-led | Length: at least 1 year;  Frequency: at least 1x;  Intensity: NA;  Duration: NA | Exposure was at least one year with one interview and 5 observations. The dance program consisted of three different but similar urban dance programs. There were additional activities and support provided by a social worker. | Interviews: open and non-directive questions;  the two part interview consisted of questions about the girls experience with the program and about personal background characteristics.  Observation: field notes after informal discussions and during and after observations. | Content analysis | The urban dance program boosted self-confidence through competence experiences, such as having a sense of accomplishment in dancing and helping others. Support from peers and coaches also strengthens confidence. Together, a sense of accomplishment and supportive relationships lead to higher self-confidence. Additionally, these programs provide opportunities to build supportive social connections, improving participants' personal growth and social bonds. |
| Scrantom & McLaughlin  2019  Northern Ireland | Heroes on the hill: A qualitative study of the psychosocial benefits of an intercultural arts programme for youth in Northern Ireland | Aiming to assess the psychosocial benefits for young people who participate in a cross-community dance program in a post-conflict society | Qualitative – longitudinal | General population  (from various background with e.g.,  refugee experiences) | n = 10;  sex ratio: 9 (90%) female, 1 (10%) male;  Age range 11-15y, mean age NA | Type: (intercultural) dancing;  Setting: community-based;  Physical E: indoor;  Social E: group-based;  Delivery: coach-led | Length: 6 weeks;  Frequency: 2x;  Intensity: NA;  Duration: 90min | The program was implemented by a professional dance company. At the end, they had a performance in front of people. | Two interviews with each participants – pre-post: first interview about expectations, aims, and friendship experiences; second interview about the overall experience in the program, the skills gained, improvement suggestions and newly formed friendships. | Thematic analysis | 3 relevant themes were identified:  Building self-confidence (via sense of mastery in the rehearsals and performances, self-confidence and self-efficacy was promoted);  Forming cross‐community friendships (sense of community with their peers, maybe because they are all “the others”); Developing intercultural awareness and pride (conscientious of different cultures and therefore “increased sense of efficacy and agency within their families and the community”). |
| Sorsdahl et al.  2024  South Africa | Experiences and Perceived Benefits of a Youth Skateboarding Program in South Africa: From the Physical to Emotional and Beyond | Aiming to assess the mental health needs and risk behaviors of adolescents who attended an after-school life-skills skateboarding program and to evaluate the perceived benefits and identify potential modifications that are required to meet the needs. | Qualitative – retrospective  (mixed-methods study, qualitative part only met the inclusion criteria of our review) | At-risk population  (socially disadvantaged) | n = 24 adolescents (+ 19 key influencers);  sex ratio: 6 (25%) female, 18 (75%) male;  Age range 11-18,  mean age NA | Type: skateboarding;  Setting: after-school;  Physical E: NA;  Social E: rather group-based;  Delivery: mixed (coach-led and self-organized) | Length: NA;  Frequency: offered 5 days a week, attendance not reported;  Intensity: NA;  Duration: NA | The sessions included 30minutes circle time at the beginning followed by skateboarding and a pulse check with a specific topic. | Semi-structured interviews with opening questions and follow-up probes. Questions were about the involvement in, opinion and impact of the program. | Framework approach by Pope et al. (2000) | The program brought a sense of belonging to the skateboard subculture, protection from gang recruitment and community violence, physical and emotional benefits like enhanced well-being and mentors played a role as positive role models. |
| Tadesse et al.  2016  Ethiopia | Benefits and challenges of practicing taekwondo to adolescents in Addis Ababa City, Ethiopia | Aiming to describe taekwondo training, to explore its benefits for well-being, and to identify challenges. | Qualitative – retrospective | General population | N = 8;  Sex ratio: 5 (63%) female, 3 (37%) male;  Age range 12-18y, mean age 14.75y | Type: Taekwondo (TKD);  Setting: community-based;  Physical E: indoor sports facility;  Social E: group-based;  Delivery: coach-led | Length: NA (retrospective);  Frequency: 3x;  Intensity: NA;  Duration: 90min | The sessions included warming up exercises, fitness/strength exercises, techniques of TKD, sparring, form practices, breaking; meditation and breathing; philosophy and ethics of TKD; self‐defense; and trainings for demonstration/graduation ceremonies | The In-depth interviews with guiding questions focused on issues like perception of benefits of TKD and problems of practicing TKD | No clear statement (analyzed following the steps suggested by Creswell (2007)) | The general finding indicates that taekwondo can improve behavioral level by more self-discipline and self-management. From the 6 emerging themes the reported social, mental and physical benefits were relevant for well-being. |
| Tejvani et al.  2016  India | Effect of Yoga on anxiety, depression and self-esteem in orphanage residents: A pilot study | Aiming to assess the effect of a two week yoga intervention on anxiety, depression and self-esteem in adolescents and young adults living in an orphanage. | Quantitative non-randomized longitudinal studies without a control group | At-risk population  (participants living in orphanage residents) | n = 34;  sex ratio: 7 (20.6%) females, 27 (79.4 %) males;  Age range 12-20y, mean age 12.27y | Type: Yoga;  Setting: NA (assuming rather in an orphanage setting;  Physical E: NA;  Social E: group-based;  Delivery: coach-led | Length: 2 weeks;  Frequency: 6x;  Intensity: NA;  Duration: 60min | The sessions included different types of yoga techniques and meditation, no more information are available | Rosenberg self‑esteem scale (10 items, general score was used) | Wilcoxon rank test  Pre-post measurement | Significant positive significant changes in self-esteem (p < .001) |
| Ullrich-French et al.  2016  USA | Evaluation development for a physical activity positive youth development program for girls | Aiming to describe the development process and pilot testing of a quantitative outcome evaluation instrument for GOTR and to develop a practical and psychometrically sound quantitative instrument to assess effectiveness using key elements of CBPR. | Qualitative longitudinal  (qualitative part only met the inclusion part for our review) | General population | n = 4;  Only female;  Age range 10-11y, mean age 10.75y | Type: running ;  Setting: community-based;  Physical E: outdoor;  Social E: group-based;  Delivery:coach-led | Length: 12 weeks;  Frequency: 2x;  Intensity: vigorous;  Duration: 90min | The curriculum of the program included integrated psycho-educational aspects, discussions etc. Every session was combined with a session topic which was discussed during warm-up and cool-down. | focus group discussions with 4 topics about the program:  What they liked most, if they would change anything, what they learned and if anything changed because of participating in the program. | Inductive generating of themes (Patton, 2015) | 3 themes were identified:  1. learning strategies for managing emotions and peer conflict, learning new skills such as making friends, speaking out and valuing different opinions. 2. Improved confidence. 3. Encouragement from running buddies also provided fundamental support, emphasizing the importance of social environment in their experience. The reported themes overall include building self-confidence, managing peer interactions and emotions, and receiving encouragement within a positive social setting. |
| Vallejos et al. 2016  UK | Kundalini yoga as mutual recovery: a feasibility study including children in care and their carers | Aiming to the test the incorporation of a 20-week Kundalini yoga programme on well-being outcomes in children in residential homes | Mixed methods  (qualitative & quantitative non-randomized longitudinal studies without a control group) | At-risk population (children in residential care) | N = 9  Sex ratio NA;  Age range 13-17y, mean age 14.78y | Type: yoga;  Setting: NA;  Physical E: indoor;  Social E: group-based;  Delivery: coach-led | Length: 20 weeks;  Frequency: 1x;  Intensity: NA;  Duration: 44-60min | Yoga style with static postures but also some high-intensive parts. | QUAN: Short Warwick-Edinburgh Mental Well-being Scale (SWEMWBS) & questionnaire about social inclusion (Secker et al., 2009); QUAL: qualitative semi-structured interviews | QUAN: NA  QUAL: thematic analysis | QUAN: No significant (p-level .05) changes in well-being and social inclusion.  QUAL (not clear whether or to what extent the answers relate to staff oder adolescents): Increased bodily awareness, improved mental health, increased ability to observe and control emotions, increase in ability to witness own negative feelings with detachment and greater sense of self-awareness. Social benefits were also reported. |
| Wildeman et al.  2025  Canada | Dance for Wellness: Indigenous adolescents’ Perspectives on Mental Health, Wellness, and Dance, | The study's aim was to gain insight into the experiences of mental health and wellness in Indigenous adolescents and identify if and how a four-week dance program affected their mental health and wellness. | Qualitative longitudinal  with two single group interviews | At-risk population  (socially disadvan-taged and/or trauma experiences) | N = 8;  Sex ratio: NA: Age range 11-16y, mean age NA | Type: dancing;  Setting: school-based;  Physical E: indoor;  Social E: in a group with interactions, but no clear team  Delivery: coach-le | Length: 4 weeks;  Frequency: 3x;  Intensity: NA;  Duration: NA | Afterschool contemporary dance program in a indigenous community | First interview about understanding of mental health and the experience of the dance program; second interview about an self-brought item and experiences in the dance program | Thematic analysis | Participants experiences dancing as: a sense of respite/escapism, improved mood, gave confidence, feeling comfortable while dancing |
| Jalivand et al. 2023  Iran | The Effectiveness of the Tactical Games Model on Self-efficacy, Physical Activity Enjoyment, and Learning of Badminton Serve | Aiming to investigate the effectiveness the tactical games model had on self-efficacy, physical activity enjoyment, and learning of badminton long serve in adolescent female students | RCT  (for our review, we took the EG & CG together because in both groups they practised badminton, thus we use it as “quantitative non-randomized longitudinal studies without a control group”) | General population | N = 30;  Only female, Age range 9-12y, mean age 10.5y | Type: badminton;  Setting: school-based (after-/before-school);  Physical E: NA;  Social E: group-based;  Delivery: coach-led | 3x a week  Intensity NA  60 minutes  Length: 8 weeks;  Frequency: 3x;  Intensity: NA;  Duration: 60min | Girls were randomized into 2 groups: Badminton with active game model or with a traditional training approach. | Self-efficacy measured with the  General Self-Efficacy Scale (Sherer, 1982) | ANCOVA and t-tests | There were significant effects in both groups, so there was an between and within effect (p < .05)  That means badminton in both forms can promote self-efficacy in girls with a superior effect in the active game model group. |

# Supplementary Table S10: Quality Assessment of included studies

--> UPDATED VERSION KOMMT NOCH

Graded with the Mixed-Methods Appraisal Method (Hong et al., 2018):

| **Study** | **Screening questions** | **Qualitative** | **Quantitative randomized** | **Quantitative non-randomized** | **Comments** |
| --- | --- | --- | --- | --- | --- |
| Beaulac et al. (2011) | ✓✓ | – ✓ ✓ ✓ ✓ |  |  | Good quality. |
| Budhraja et al. (2025) | ✓✓ | ✓✓✓✓✓ |  |  | High methodological rigor, even though secondary analysis of the data |
| ^1^Carlin et al. (2025) | ✓✓ | -- ✓✓✓✓ | ✓ ✓ ✓ – – |  | Mixed-methods study, not a lot of information on the qualitative part, but overall good |
| Case et al. (2019) | ✓✓ | – ✓ ✓ – – |  |  | The analysis and conclusions are not very coherent. |
| Duberg et al. (2016) | ✓✓ | – ✓ ✓ ✓ ✓ |  |  | This study appears to be of very high quality. |
| 1Godfrey et al. (2015) | ✓✓ | – – ✓ – ✓ |  |  | Qualitative part only used as a supplement, almost no information on the procedure and results, this reduces the quality. |
| 1Hignett et al. (2018) | ✓– |  |  |  | The qualitative part of this mixed methods study was very sparse and not related to the RQ. For this reason, the qualitative part was excluded from the synthesis. |
| Kernebone et al. (2022) | ✓✓ | ✓ ✓ ✓ ✓ ✓ |  |  | Thoroughly conducted study |
| Lai et al. (2021) | ✓✓ | – ✓ ✓ ✓ ✓ |  |  | Overall coherent study, deduction only due to unclear qualitative approach |
| Ryan et al. (2025) | ✓✓ | ✓ ✓ ✓ ✓ ✓ |  |  | Overall high methodological quality and rigor |
| Scrantom et al. (2019) | ✓✓ | ✓ ✓ ✓ ✓ ✓ |  |  | Overall coherent and high quality |
| Sorsdahl et al. (2024) | ✓✓ | – ✓ ✓ ✓ ✓ |  |  | Overall, quite appropriate, but probably because mixed methods study, qualitative justification not quite perfect |
| Tadesse et al. (2016) | ✓✓ | – ✓ ✓ – ✓ |  |  | A mixed-methods study from which only the qualitative part fits to our inclusion criteria. Methodologically not thoroughly explained. |
| 1,2Vallejos et al. (2016) | ✓✓ | – ✓ ✓ ✓ – |  | – – – – – | A mixed-methods study and regarding inclusion criteria, both the quantitative and qualitative part seems to fit to our review which are assessed separately:  QUAN: very sparse information on the sample and overall unclear approach, no criterion fulfilled.  QUAL: Again here sparse information on participants, and regarding inclusion criteria not clear, whether or which findings relate to the participants which fulfills the inclusion criteria. |
| Wildeman et al. (2025) | ✓✓ | – ✓ ✓ ✓ ✓ |  |  | Overall, this is a well-conducted and coherent study. The only notable shortcoming is the absence of a clear philosophical and/or epistemological standpoint |
| Marshal et al. (2019) | ✓✓ | ✓ ✓ ✓ ✓ ✓ |  |  | Overall methodically very thoroughly conducted. |
| Ullrich-French et al. (2015) | ✓✓ | – ✓ ✓ ✓ – |  |  | Evaluation study with a rather small part on qualitative aspects, which will then be used to develop a quantitative evaluation instrument.  Many aspects were narrowly met, methodological aspects were not very extensively described. |
| Abbott et al. (2014) | ✓✓ |  | ✓ ✓ ✓ – – |  | A lot of aspects with high quality, but manipulation check and no information whether/how often participants have performed the sessions in the respective experimental condition. |
| Bahram et al. (2014) | ✓✓ |  | – ✓ – – – |  | Study is not cited as an RCT by authors, though it meets the criteria for this, but certain things are not reported very comprehensively, which gives ‘deduction’. |
| Duncan et al. (2009) | ✓✓ |  | – – ✓ ✓ ✓ |  | Methodological process not very comprehensively reported, but most criteria narrowly met. |
| ^2^Gerguz & Aras Bayram (2023) | ✓✓ |  | ✓ ✓ ✓ – – |  | Large problem: contradictory findings (text vs. tables) and no clarification after inquiring the authors (no response). Thus, study was excluded. |
| Greco et al. (2019) | ✓✓ |  | ✓ ✓ ✓ – – |  | Not all information available (e.g., adherence), but overall good quality |
| ^3^Li et al. (2025) | ✓✓ |  | ✓ ✓ ✓ – ✓ |  | Overall, the methodology appears to be good; however, there is limited information on the control group, and there is a lack of descriptive statistics (no meta-analysis possible). |
| Lubans et al. (2010) | ✓✓ |  | – – ✓ – ✓ |  | Control group appears to be recruited post-hoc and no information about their randomization. A lot of measures and conditions included, thus, an elaborate design. Some inconsistencies. |
| Mona et al. (2025) | ✓✓ |  | ✓✓✓✓– |  | Based on the reported information, the study was conducted with methodological rigor, although information on adherence is lacking |
| Philipsson et al. (2013) | ✓✓ |  | ✓ ✓ ✓ – – |  | No blinding for the CG and rather low adherence, other aspects good. |
| Jalilvand et al. (2023) | ✓✓ |  | –✓✓✓✓ |  | Overall good quality |
| Jago et al. (2015) | ✓✓ |  | ✓ ✓ ✓ – – |  | A very large study, but the relevant outcomes for our review were not the primary outcomes, also problems with adherence, and blinding for CG not reported. The other aspects conducted thoroughly. |
| Berger et al. (2009) | ✓✓ |  | – ✓ ✓ ✓ – |  | Confounders handled extensively - good, but low adherence and no clear in- & exclusion criteria. |
| Das et al. (2016) | ✓✓ |  | ✓ ✓ ✓ – ✓ |  | Overall good quality, but confounders not extensively accounted for. |
| Diaz-Hidalgo (2024) | ✓✓ |  | ✓ ✓ ✓ – ✓ |  | Overall good quality, but internal validity questionable with this sample and many possible confounders. |
| Dubey et al. (2011) | ✓✓ |  | – ✓ – – ✓ |  | Some relevant information of the intervention is missing, thus high quality cannot be ensured. |
| Pereira et al. (2017) | ✓✓ |  | – ✓ – ✓ ✓ |  | Non specific in- & exclusion criteria and relatively high attrition, the others parts were conducted. |
| Choukse et al. (2018) | ✓✓ |  |  | ✓ ✓ ✓ – – | Methodologically not comprehensively described, grading of the study was difficult and confounders appears to be possible. |
| DeBate et al.  (2009) | ✓✓ |  |  | ✓ ✓ – – ✓ | Large sample size but also high attrition, multiple possible confounders not considered. |
| Décarpentrie et al. (2024) | ✓✓ |  |  | – – – ✓– | This study has an extremely high attrition rate—likely due to COVID-19, as noted in the discussion. The exact content of the intervention remains unclear, and the measurement tools used exhibit very poor reliability, indicating several methodological ambiguities. |
| 1Godfrey et al. (2015) | ✓✓ |  |  | – – – ✓ ✓ | A mixed-methods evaluation study and some scientific relevant information were not comprehensively reported. Confounder effects accounted for, but e.g. no clear in- & exclusion criteria. |
| ^[[1]](#footnote-2)^Hignett et al. (2018) | ✓✓ |  |  | ✓ ✓ – – ✓ | Study not always thoroughly described, but also contains many good aspects |
| Kwasky et al. (2018) | ✓✓ |  |  | ✓ ✓ ✓ – – | Overall appears to be a good quality, but, scares reporting on the procedure. |
| La ‘I-Kjeirkhah et al. (2019) | ✓✓ |  |  | ✓ ✓ ✓ – ✓ | Methodologically good study overall |
| Luttenberger et al. (2024) | ✓✓ |  |  | ✓ ✓ – ✓ ✓ | The study was fundamentally well conducted, with the use of an intention-to-treat (ITT) analysis being a particular strength; however, the attrition rate was high. |
| Teijvani et al. (2016) | ✓✓ |  |  | ✓ ✓ ✓ – ✓ | Appears to be good quality but not considering possible confounders. |
| Rinaldo et al. (2016) | ✓✓ |  |  | – ✓ – – – | A short communication report, almost no information about the sample and procedure, thus, quality seems to be limited. |
| Rauscher et al. (2013) | ✓✓ |  |  | – ✓ – – ✓ | Lack of information for the study’s methodologically (e.g., sample, confounders), indicating a limited quality |

^1^Mixed-methods study, the quantitative and qualitative part were estimated separately only due to the separate usage in this review.

^2^Study excluded for the synthesis.

^3^Study excluded for the meta-analysis because no descriptive values were available, also not upon request

# Supplementary Section S11: Detailed information about the meta-analysis

The multivariate meta-analyses were calculated per LTPA type with the rma.mv-command from the R-package meta (Viechtbauer, 2010) because multiple outcomes in the same study occurred. Thus, a variance-covariance-matrix between the respective outcomes within the same study has to be created. The following rules for estimating the correlations between the two outcomes of the same study were applied:

- If the same PMH domain AND the same PMH feature: 0.7
- If the same PMH domain OR the same PMH feature: 0.6
- If different PMH domain and PMH feature: 0.5

After setting up all the variance-covariance-matrices, the meta-analyses were calculated with the following example code:

*MA_LTPAtype_X <- rma.mv(yi = SMD,*

*V = V_LTPAtypeX,*

*random = ~ 1 | id,*

*data = data_LTPAtypeX,*

*method = "REML")*

We conducted no sensitivity analysis due to the fact that multiple types of interventions were already splitted up (LTPA types), and sensitivity analyses within LTPA types were due to the small numbers of studies not possible.

As Borenstein (2019) describes in his chapter, in the case of very low number of studies, heterogeneity metrics (e.g., I^2^, prediction interval) are unreliable and misleading for the interpretation. Thus, we reported no heterogeneity measures and emphasised the carefulness when interpreting the findings in the manuscript.

The confidence of each meta-analysis is indicated with the 95% confidence interval of the mean effect.

# Supplementary Figure S12: Detailed synthesis of the qualitative studies with all themes


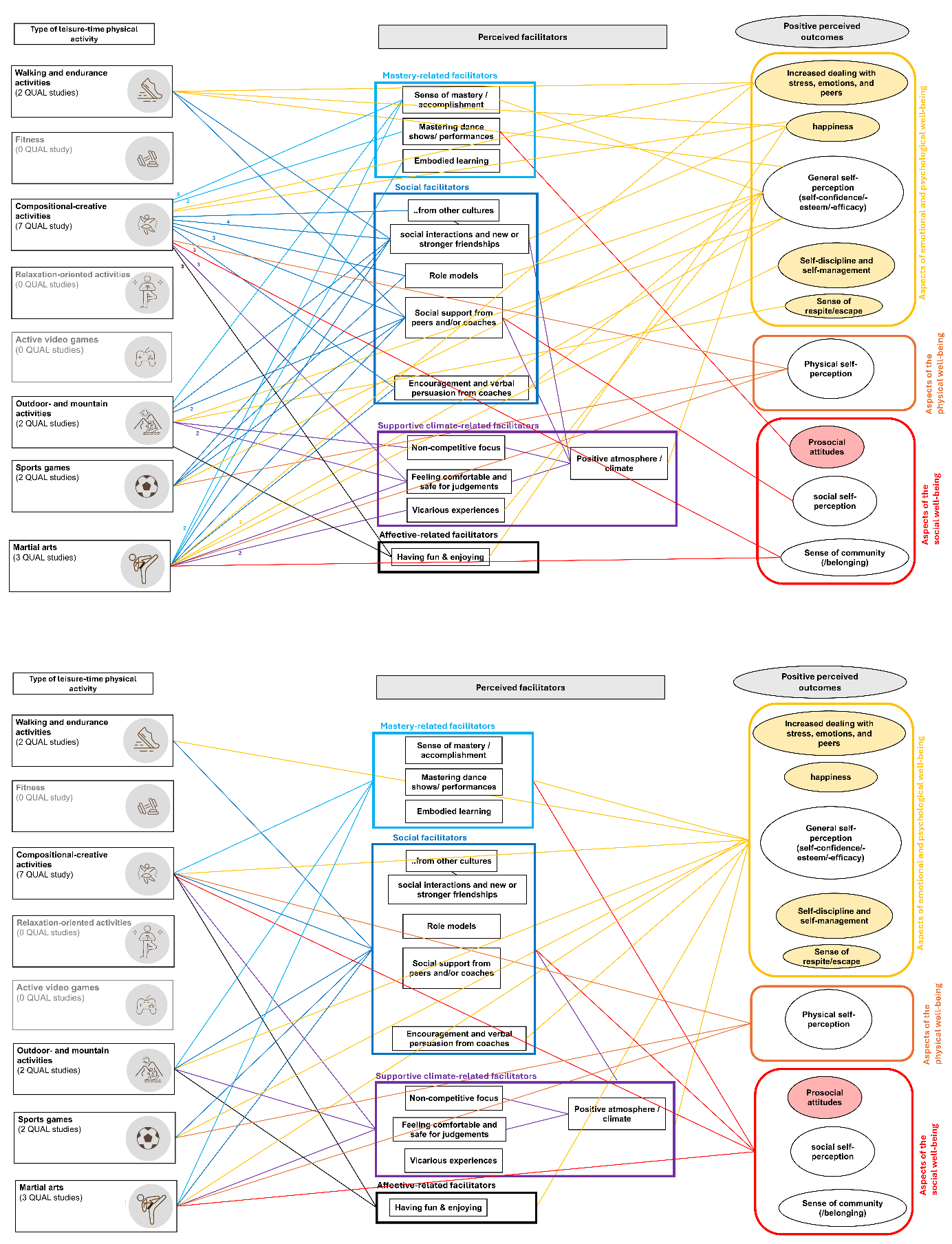


*Upper part: every single relationship is displayed, indicating the number of studies describe the respective relationship (if more than one); lower part: relationships between every organizing themes; Legend: the basic themes within each well-being domain were coloured if these perceived outcomes did not occur in the quantitative studies*

1. [↑](#footnote-ref-2)
